# Change in lipids before onset of dementia, coronary heart disease, and mortality: A 28‐year follow‐up Whitehall II prospective cohort study

**DOI:** 10.1002/alz.13140

**Published:** 2023-05-27

**Authors:** Céline Ben Hassen, Marcos D Machado‐Fragua, Benjamin Landré, Aurore Fayosse, Julien Dumurgier, Mika Kivimaki, Séverine Sabia, Archana Singh‐Manoux

**Affiliations:** ^1^ Université Paris Cité, Inserm U1153, Epidemiology of Ageing and Neurodegenerative diseases Paris France; ^2^ Cognitive Neurology Center, Lariboisière – Fernand Widal Hospital, AP‐HP Université Paris Cité Paris France; ^3^ Department of Mental Health of Older People, Faculty of Brain Sciences University College London London UK

**Keywords:** dementia, lipids

## Abstract

**INTRODUCTION:**

The association of lipids with dementia remains a subject of debate. Using data from 7,672 participants of the Whitehall II prospective cohort study, we examined whether timing of exposure, length of follow‐up, or sex modifies this association.

**METHODS:**

Twelve markers of lipid levels were measured from fasting blood and eight among them a further five times. We performed time‐to‐event as well as trajectory analyses.

**RESULTS:**

No associations were observed in men; in women most lipids were associated with the risk of dementia, but only for events occurring after the first 20 years of follow‐up. Differences in lipid trajectories in men emerged only in the years immediately before diagnosis whereas in women total cholesterol (TC), LDL‐cholesterol (LDL‐C), non‐HDL‐cholesterol (non‐HDL‐C), TC/HDL‐C, and LDL‐C/HDL‐C were higher in midlife among dementia cases before declining progressively.

**DISCUSSION:**

Abnormal lipid levels in midlife seem to be associated with a higher risk of dementia in women.

## 1 BACKGROUND

Dysfunction of the blood‐brain barrier is thought to be involved in several neurological diseases, including Alzheimer's disease (AD) and related dementias.^1^, ^2^, ^3^ This has led to the suggestion that systemic and peripheral processes rather than only those originating in the central nervous system may be involved in shaping the risk of dementia. Serum lipids have attracted considerable attention as they are modifiable; the brain is a lipid‐rich organ and lipids play an important role in vascular outcomes, themselves deemed to be important for dementia.^4^

The association of lipids with coronary heart disease^5^, ^6^ and mortality^7^ in longitudinal studies and trials of statin (a lipid‐lowering drug reducing low‐density lipoprotein cholesterol [LDL‐C] and total cholesterol [TC] concentrations) is well established. The association with dementia remains the subject of debate,^8^ with some evidence showing lipids measured in midlife but not in late life to be associated with dementia or cognitive decline.^9^, ^10^, ^11^, ^12^, ^13^, ^14^, ^15^ It is possible that inconsistent findings reflect the fact that the pathophysiological processes underlying dementia, AD in particular, unfold over a long period, perhaps as long as 15 to 20 years.^16^ Accordingly, some studies show a decline in total cholesterol from midlife to older ages to be associated with dementia.^17^, ^18^

Besides the length of follow‐up in studies, a further source of heterogeneity in results on the association between lipids and dementia is sex. Rates of dementia are higher in women than men; the longer life expectancy in women contributes to some of these differences but differences in the effects of cerebrovascular, metabolic, and socioeconomic factors may also play a role.^19^ A recent study found that the combined impact of eight risk factors (education, hearing loss, traumatic brain injury, alcohol/substance abuse, hypertension, smoking, diabetes, and depression) on cognitive decline was greater in women than in men.^20^ In women but not men a better lipid profile has been shown to predict maintenance of cognitive abilities at older ages.^21^ There is also some evidence to suggest that women are more vulnerable to the deleterious effects of the apolipoprotein E (*APOE*) *ε*4 allele for cognitive outcomes, decline in episodic memory^22^ and AD.^23^, ^24^, ^25^

The most commonly examined lipids are TC, LDL‐C, high‐density lipoprotein cholesterol (HDL‐C), and triglycerides. In prospective observational studies, higher levels of TC^10^, ^14^, ^26^ and LDL‐C^14^, ^26^, ^27^ have been shown to be associated with higher risk of dementia. There is also some evidence for a lower risk of dementia in individuals with higher levels of HDL‐C.^28^ Use of statins was associated with a decreased risk of dementia in observational studies,^29^, ^30^ although a systematic review found no clear association with cognitive function in observational studies or in clinical trials;^31^ the follow‐up was less than 6 years in the trials and 3 to 15 years in observational studies. Few prospective studies have examined the role of triglycerides, and no associations were found with dementia when triglycerides were measured in midlife^14^ or at older ages.^26^ Recent research highlights the importance of apolipoproteins, proteins that mediate carriage of cholesterol and other lipids in the serum, for cardiovascular disease,^32^, ^33^ but a 10‐year follow‐up in the FINRISK study did not find an association between apolipoproteins and dementia.^34^

Our aim was to examine the association of 12 markers of lipid levels (including lipoprotein and apolipoproteins) with the risk of dementia in time‐to‐event analyses using a median follow‐up of 27 years, with a special focus on the role of length of follow‐up and sex in these analyses. A second aim was to use repeated data on lipids to determine whether changes over 28 years leading to dementia diagnosis differ from changes in lipids among those who remain dementia‐free. Besides dementia, we examined coronary heart disease (CHD) and mortality as secondary outcomes to allow comparison with results on dementia and findings from other studies on these outcomes.

**RESEARCH IN CONTEXT**

1. **Systematic Review**: The authors reviewed the literature using PubMed and reported key publications. The association of several lipids with coronary heart disease and mortality is well established, but results regarding dementia remain inconsistent. Potential reasons for inconsistency in the findings include lack of consideration for sex differences, and lack of consideration to the long preclinical period of dementia involving pathophysiological changes over 15 to 20 years before diagnosis. Whether peripheral lipid levels have any association with dementia remains unclear.
2. **Interpretation**: Using data spanning 28 years for 7,672 participants, mean age 50 years at baseline, several lipid markers were found to be associated with dementia in time‐to‐event analyses in women, but only when the follow‐up was longer than 20 years. Analyses of lipid trajectories showed greater differences between dementia cases and non‐cases in women in midlife, differences that were attenuated in late life. Results were consistent with other studies and similar in men and women when it came to association with mortality and coronary heart disease.
3. **Implications of all the available evidence**: Findings of the present study suggest that abnormal lipid levels in midlife are associated with a higher risk of dementia in women.

## 2 METHODS

### 2.1 Study population

The Whitehall II study is an ongoing cohort study of 10,308 persons (6,895 men and 3,413 women, aged 35 to 55 years) employed in the London‐based British civil service at recruitment to the study in 1985 to 1988.^35^ Since baseline, follow‐up clinical examinations have taken place every 4 to 5 years (1991 to 1993, 1997 to 1999, 2002 to 2004, 2007 to 2009, 2012 to 2013, 2015 to 2016, and an ongoing wave), with each wave taking 2 years to complete. Lipids were first measured in the study in the 1991 to 1993 wave of data collection.

Participant data are linked to UK National Health Service (NHS) electronic health records for all but ten participants (99.9%). The NHS provides most of the health care in the UK, including in‐ and outpatient care, and record linkage is undertaken using a unique NHS identifier held by all UK residents. Data from linked records were updated on an annual basis, until March 31, 2019.

Written informed consent from participants and research ethics approvals were renewed at each contact; the most recent approval was granted by the University College London Hospital Committee on the Ethics of Human Research (reference number 85/0938).

### 2.2 Lipid panel

A total of 12 markers of lipids were measured in 1991 to 1993; of these eight were measured a further 5 times between 1997 and 2016 (1997 to 1999, 2002 to 2004, 2007 to 2009, 2012 to 2013, 2015 to 2016).

For the lipids measured at baseline (1991 to 1993), blood samples using a standardized protocol were collected after fasting either overnight or 4 h after a light fat‐free breakfast. Part of the sample, refrigerated at −4°C, was assayed within 72 h. *TC* and *HDL‐C* were measured using the CHOD‐PAP method; *triglycerides* were determined by the enzymatic colorimetric method (GPO‐PAP). The concentration of *LDL‐C* was calculated using the Friedewald formula.^36^ Technical error was estimated by assaying blinded duplicate samples for 5% of the subjects; coefficients of variation were 2.0% to 6.6%. From these were derived the following: *non‐HDL‐C* (TC minus HDL‐C), *TC/HDL‐C ratio*, *LDL‐C/HDL‐C ratio*, and the *atherogenic index of plasma* (AIP) as Lg_10_(triglycerides/HDL‐C).^37^ Serum stored at −70°C was used for the measure of *apolipoprotein A1* (ApoA1), *apolipoprotein B* (ApoB), and *lipoprotein A* (Lp[a]) using the immunoturbidimetric method.^38^ In the analyses we also used the *ApoB/ApoA1 ratio*.

The repeated measures of lipids in 1991 to 1993 and 2015 to 2016 included all markers apart from Lp(a), apolipoproteins B and A1, and their ratio.

### 2.3 Primary outcome: Dementia

Dementia cases were ascertained by linkage to the Hospital Episode Statistics (HES) database, the Mental Health Services Data Set, and the mortality register up to March 31, 2019. All‐cause dementia was identified based on ICD‐10 codes F00 to F03, F05.1, G30, and G31. The sensitivity and specificity of dementia ascertainment based on HES data are 78.0% and 92.0%, respectively.^39^ The sensitivity in our study is likely to be further improved due to use of Mental Health Services Data Set, a national database that contains information on dementia for persons in contact with mental health services in hospitals, outpatient clinics, and the community.^40^ Date of dementia was set at the first record of dementia diagnosis using all three databases.

### 2.4 Secondary outcomes

#### 2.4.1 All‐cause mortality

Death records until March 31, 2019 were drawn from the British national mortality register (NHS Central Registry) using the NHS identification number of each participant.

#### 2.4.2 Coronary heart disease (CHD)

Data were drawn from clinical examinations in the study (12‐lead resting ECG recording) and linkage to electronic health records from HES using NHS identification number (ICD10: I20 to I25), up to March 31, 2019.

### 2.5 Covariates

Covariates included sociodemographic characteristics (age, sex, ethnicity [white or non‐white], marital status [married/cohabiting, other], education [lower secondary school or lower, secondary school, university]); socioeconomic position (using a 6‐level measure of employment grade that reflects education, salary, and status at work; modelled as a continuous variable); use of lipids‐lowering drugs; obesity (defined as Body Mass Index ≥ 30 kg/m^2^); and various health behaviors (smoking [never smoker, former smoker, current smoker], alcohol consumption [none in the previous week, 1 to 14 units/week, >14 units/week], time spent in moderate and vigorous physical activity [hours per week], and frequency of fruit and vegetable consumption [less than daily, once per day, twice or more per day]). In additional analyses, we also used measures of hypertension (systolic/diastolic ≥ 140/90 mmHg or use of antihypertensive medication), diabetes (ICD10: E10 to E14, reported doctor‐diagnosed diabetes, use of diabetes medication, or fasting glucose ≥ 7.0 mmol/L), and self‐reported age at menopause.

### 2.6 Statistical analysis

Participants with prevalent dementia and CHD at the start of follow‐up (1991 to 1993) and participants with missing data on exposures or covariates at baseline were excluded from the analyses. LP(a) and triglyceride data were normalized (log scale) and all lipids were standardized to z‐scores (mean 0, standard deviation 1).

### 2.7 Time‐to‐event analyses

The association of each lipid (separate models), measured in 1991 to 1993, with incident dementia was examined using Cox proportional‐hazards regression with age as the time scale. Participants were followed up until the date of record of dementia, death, or March 31, 2019, whichever came first. Censoring at date of death was undertaken to account for competing risk of death, reflecting cause‐specific hazard models.^41^ Preliminary analyses showed a statistically significant interaction between a number of lipid measures and sex, leading to analyses separately in men and women.

The proportional hazards assumption was examined by plotting Schoenfeld residuals, and violation of this assumption led us to stratify the analyses on the length of the follow‐up (<20 years or ≥20 years, to reflect the long preclinical phase of dementia^16^) using data on lipids at baseline (1991 to 1993) in both cases. For the analyses with follow‐up < 20 years, the end of follow‐up was within the first 20 years on the date of dementia or death, or at the end of 20 years from baseline, whichever came first. The analyses using a follow‐up of ≥20 years were based on participants who were alive and free from dementia 20 years after the baseline lipids measurement in 1991 to 1993; the start of the follow‐up in these analyses was the baseline date + 20 years and the end was date of dementia, death, or March 31, 2019, whichever came first. The analyses were first adjusted for age (time‐scale), education, socioeconomic position, ethnicity, marital status, and use of lipids‐lowering drugs, allowing the baseline hazard to differ by birth‐cohort (5‐year groups) as Model 1; next, this was also adjusted for obesity and health‐related behaviors (smoking status, alcohol consumption, fruit and vegetable consumption, physical activity) as Model 2. In these analyses all lipids and covariates were drawn from the 1991 to 1993 clinical examination. The analyses were repeated for the secondary outcomes, mortality and CHD.

### 2.8 Trajectories of lipids using a backward time‐scale

These analyses were undertaken using repeated data on lipids to examine differences in lipid trajectories between those who developed dementia during the follow‐up period (1991 to 1993 and March 31, 2019) and all others. A backward timescale was used such that time t = 0 was the date of dementia diagnosis for dementia cases and end of the follow‐up (March 31, 2019) or date of death for non‐cases.^42^ The trajectories of lipids over a maximum of 28 years were examined separately in men and women using linear mixed effects models with random intercept and slope. For each lipid, the model included dementia status at t = 0, ethnicity, education, age at t = 0, time‐varying covariates (use of lipids‐lowering drugs, marital status, socioeconomic position, obesity, health‐related behaviors, an indicator of the baseline lipid measure [0 = baseline measure, 1 = other follow‐up measures] to account for period effects [pre‐statin era]), time terms (time, time sqared; or time, time sqared, time cubed, based on Akaike Information Criteria), and the interactions of time terms with dementia status at t = 0 and with age at t = 0. From the fitted model we used marginal means to plot trajectories of lipids in the 28 years before t = 0 in men and women; *P*‐values for differences in overall lipid trajectories over 28 years between cases and non‐cases were estimated using the interaction between time terms and dementia status. Difference in lipid levels between cases and non‐cases were also extracted every 5 years from t = −25 to t = 0 and compared using paired *t*‐tests. The analyses of trajectories were repeated for CHD and mortality.

Additional analyses were undertaken in order to investigate the potential role of lipid‐lowering drugs, *APOE* genotype, cardiovascular risk factors, and menopausal status in women. This consisted of the following: (1) repeating the Cox regression using lipid‐lowering drugs treated as a time‐varying measure; (2) repeating the analyses on individuals without the *APOE ε*4 allele to examine whether the association of lipids with dementia (and the secondary outcomes) remained unchanged in this group, and then similar analyses were undertaken on individuals without the *APOE ε*2 allele; (3) repeating the Cox regression analyses with further adjustment for hypertension and diabetes at baseline; and (4) using Cox regression in women with further adjustment for menopausal status at baseline, and then using this measure as a time‐varying covariate in the trajectory analyses.

For all analyses two‐sided *P*‐values were used with an α = 0.05 threshold for statistical significance. We then used the false discovery rate procedure to account for multiple testing. All analyses were undertaken using R version 4.0.3 (R Core Team) and packages *survival*, *lme4*, *lmerTest*, and *emmeans*.

## 3 RESULTS

### 3.1 Sample characteristics

Of the 8,815 participants at the 1991 to 1993 wave, the baseline of the present study, nine were excluded as they did not consent to follow‐up, 988 due to missing data on lipids (*N* = 952) or covariates (*N* = 37), and 145 because of prevalent CHD at baseline, leading to a final analytic sample of 7,672 participants (see the flow‐chart in Figure S1). Over a median follow‐up of 26.8 (interquartile range 26.2, 27.1) years, 462 participants were diagnosed with dementia, 1,513 with CHD, and 1,390 died. Sample characteristics at baseline of participants as a function of sex and dementia status at the end of follow‐up are shown in Table 1, and as a function of sex and mortality status and CHD status in Table S1 and Table S2, respectively. Compared to dementia‐free men, those with a dementia diagnosis at follow‐up were older, more likely to be non‐white, less educated, to have a lower socioeconomic position, and to use lipids‐lowering drugs at baseline (all *P <* 0.05). Women with a dementia diagnosis at follow‐up were older, more likely to be less educated and not to drink alcohol (all *P <* 0.001). TC, LDL‐C, non‐HDL‐C, TC/HDL‐C ratio, LDL‐C/HDL‐C ratio, ApoB, Lp(a), triglycerides, ApoB/ApoA1 ratio, and AIP ratio at baseline were higher in women diagnosed with dementia over the follow‐up compared to women who remained dementia‐free (all *P <* 0.05, Table 2); no differences were found in men. All lipid levels except ApoA1 at baseline differed as a function of mortality status at follow‐up in men and women (all *P <* 0.01, Table S3); this was the case for all lipids when examined as a function of CHD at follow‐up in men and women (all *P <* 0.001, Table S4).

**FIGURE 1:**
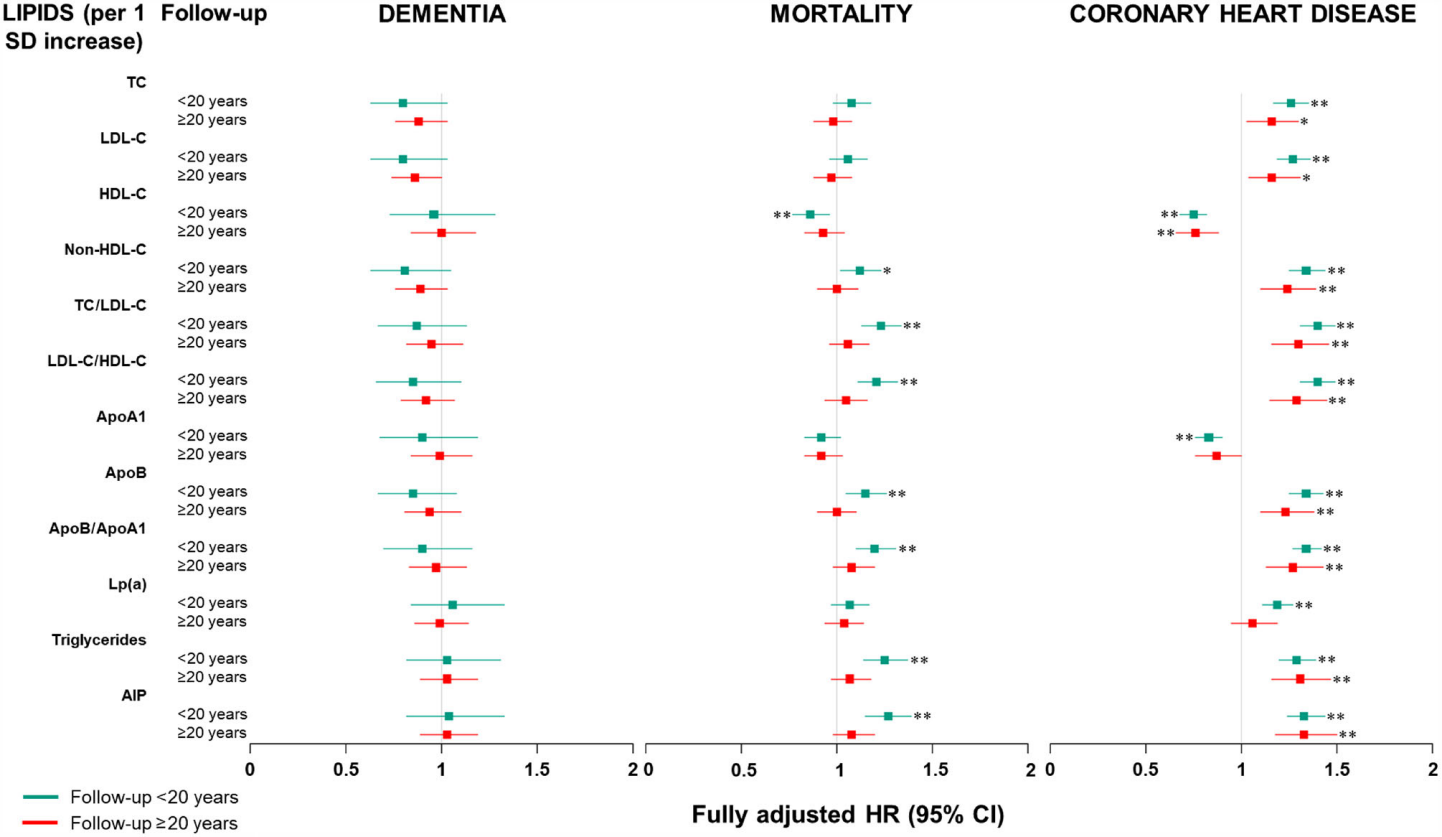
Association of lipids (1991 to 1993) with dementia, mortality, and coronary heart disease (CHD) over the follow‐up (until March 2019) stratified by the length of follow‐up in men. For follow‐up of <20 years; *N* of cases/total = 77/5,306 for dementia, 504/5,306 for mortality, and 824/5,306 for CHD. In the follow‐up of ≥20 years the corresponding numbers were 205/4,758 for dementia, 448/4,802 for mortality, and 307/4,088 for CHD. Model adjusted for age (as time scale), ethnicity, marital status, education, socioeconomic position, use of lipids‐lowering drugs, and health‐related behaviors (smoking, alcohol consumption, physical activity, diet, and obesity), allowing the baseline hazard to differ by birth‐cohort (5‐year groups). Adjustment of the *P*‐values after correction for multiple testing with false discovery rate analysis did not change the conclusions. Abbreviations: AIP, atherogenic index of plasma; ApoA1, apolipoprotein A1; ApoB: apolipoprotein B; CI, confidence interval; HDL‐C, high‐density lipoprotein cholesterol; HR, hazard ratio; LDL‐C, low‐density lipoprotein cholesterol; Lp(a), lipoprotein A; TC, total cholesterol. *P < 0.05, **P < 0.01.

**TABLE 1:** Characteristics of participants at baseline (1991 to 1993) according to dementia status at the end of the follow‐up

	Men			Women		
Participant characteristics	Dementia status (March 2019)			Dementia status (March 2019)		
	Dementia	No dementia		Dementia	No dementia	
***N***	***N* = 282**	***N* = 5,024**	*P‐*value	***N* = 180**	***N*** = **2,186**	*P‐* value
Age, M (SD)	54.9 (4.9)	49.4 (5.9)	<0.0001	55.7 (4.8)	50.2 (6.0)	<0.0001
Education						
Lower secondary school or lower	131 (46.5)	1,937 (38.6)	0.0271	139 (77.2)	1,283 (58.7)	<0.0001
Secondary school	69 (24.5)	1,474 (29.3)		24 (13.3)	478 (21.9)	
University degree or higher	82 (29.1)	1,613 (32.1)		17 (9.4)	425 (19.4)	
Socioeconomic position						
1 (high)	59 (20.9)	1,128 (22.5)	0.0340	6 (3.3)	142 (6.5)	<0.0001
2	76 (27.0)	1,310 (26.1)		9 (5.0)	229 (10.5)	
3	34 (12.1)	868 (17.3)		3 (1.7)	182 (8.3)	
4	49 (17.4)	903 (18.0)		22 (12.2)	332 (15.2)	
5	37 (13.1)	506 (10.1)		39 (21.7)	494 (22.6)	
6 (low)	27 (9.6)	309 (6.2)		101 (56.1)	807 (36.9)	
Ethnicity						
White	250 (88.7)	4,679 (93.1)	0.0063	150 (83.3)	1,877 (85.9)	0.4116
Non‐white	32 (11.3)	345 (6.9)		30 (16.7)	309 (14.1)	
Marital status						
Married/Cohabiting	237 (84.0)	4,141 (82.4)	0.5382	108 (60.0)	1,377 (63.0)	0.4728
Single/divorced/widowed	45 (16.0)	883 (17.6)		72 (40.0)	809 (37.0)	
Use of lipid‐lowering drugs	7 (2.5)	30 (0.6)	0.0009	2 (1.1)	11 (0.5)	0.5919
**Health behaviors**						
Smoking						
Never smoker	143 (50.7)	2,423 (48.2)	0.5405	90 (50.0)	1,209 (55.3)	0.2051
Former smoker	110 (39.0)	1,984 (39.5)		53 (29.4)	631 (28.9)	
Current smoker	29 (10.3)	617 (10.3)		37 (20.6)	346 (15.8)	
Hours of MVPA per week, M (SD)	4.09 (4.40)	3.90 (3.87)	0.4674	2.67 (4.04)	2.57 (4.05)	0.7421
Alcohol consumption						
0 unit/week	51 (18.1)	713 (14.2)	0.1679	83 (46.1)	633 (29.0)	<0.0001
1‐14 units/week	145 (51.4)	2,782 (55.4)		86 (47.8)	1,339 (61.3)	
> 14 units/week	86 (30.5)	1,529 (30.4)		11 (6.1)	214 (9.8)	
Fruit/vegetable consumption						
< Once/day	126 (44.7)	2,040 (40.6)	0.3965	62 (34.4)	739 (33.8)	0.2284
≥ Once/day	156 (55.3)	2,984 (59.4)		118 (65.6)	1,447 (66.2)	
Obesity	22 (7.8)	337 (6.7)	0.5554	36 (20.0)	326 (14.9)	0.0864

Abbreviations: M, mean; MVPA, moderate and vigorous physical activity; SD, standard deviation.

Data are N (%) unless stated otherwise.

The non‐white group was composed of 244 South Asian, 98 black, and 33 others among men; and 141 South Asian, 163 black, and 25 others among women.

**TABLE 2:** Lipid levels at baseline (1991 to 1993) according to dementia status at the end of the follow‐up

	Men			Women		
Participant characteristics	Dementia status (March 2019)			Dementia status (March 2019)		
	Dementia	No dementia		Dementia	No dementia	
*N*	*N* = 282	*N* = 5024	*P‐*value	*N* = 180	*N* = 2186	*P*‐value
**Lipids, M (SD)**						
Total cholesterol, mmol/L	6.48 (1.13)	6.44 (1.10)	0.5307	7.06 (1.31)	6.45 (1.19)	<0.0001
LDL‐cholesterol, mmol/L	4.44 (1.00)	4.44 (1.00)	0.9989	4.80 (1.18)	4.23 (1.09)	<0.0001
HDL‐cholesterol, mmol/L	1.34 (0.37)	1.33 (0.35)	0.5697	1.63 (0.43)	1.69 (0.43)	0.0722
Non‐HDL‐cholesterol, mmol/L	5.14 (1.12)	5.11 (1.13)	0.6575	5.43 (1.30)	4.76 (1.22)	<0.0001
Total cholesterol/HDL‐cholesterol	5.13 (1.52)	5.15 (1.57)	0.8234	4.61 (1.44)	4.07 (1.46)	<0.0001
LDL‐cholesterol/HDL‐cholesterol	3.53 (1.20)	3.57 (1.26)	0.5568	3.17 (1.19)	2.72 (1.24)	<0.0001
Apolipoprotein A1 (ApoA1), mmol/L	2.08 (0.34)	2.06 (0.32)	0.3473	2.31 (0.38)	2.33 (0.38)	0.5657
Apolipoprotein B (ApoB), mmol/L	1.32 (0.29)	1.30 (0.29)	0.3145	1.37 (0.33)	1.20 (0.30)	<0.0001
ApoB/ApoA1	0.65 (0.18)	0.65 (0.18)	0.8861	0.61 (0.18)	0.53 (0.18)	<0.0001
Lipoprotein(a) (log)	3.14 (0.89)	3.08 (0.88)	0.2385	3.30 (0.92)	3.15 (0.91)	0.0407
Triglycerides (log)	0.31 (0.50)	0.26 (0.51)	0.1199	0.21 (0.49)	0.03 (0.48)	<0.0001
Atherogenic index of plasma (AIP)	0.05 (0.68)	0.01 (0.68)	0.3219	‐0.25 (0.66)	‐0.47 (0.64)	<0.0001

Abbreviations: HDL, high‐density lipoprotein; LDL, low‐density lipoprotein.

### 3.2 Time‐to‐event analyses

Preliminary analyses, not stratified for sex or length of follow‐up, showed none of the lipids to be associated with dementia in analyses adjusted for sociodemographic factors, including sex (data not tabulated). The proportional hazards assumption was not supported in these analyses, but splitting the follow‐up at year 20 resolved this issue for all lipids apart from triglycerides (Table S5). As there were sex differences in the association of several lipids with all three outcomes (Table S6), subsequent analyses were split according to length of follow‐up and stratified by sex.

Figure 1 and Table S7 show the association of lipids with all three outcomes in men. None of the lipids were associated with the risk of dementia, irrespective of the length of follow‐up. The association of lipids with mortality was confined to analyses with a follow‐up of less than 20 years for HDL‐C, non‐HDL‐C, TC/HDL‐C ratio, LDL‐C/HDL‐C ratio, ApoB, ApoB/ApoA1 ratio, triglycerides, and AIP. All lipids were associated with CHD irrespective of the length of follow‐up, apart from ApoA1 and Lp(a), for which the association was seen only when the follow‐up was less than 20 years.

**FIGURE 2:**
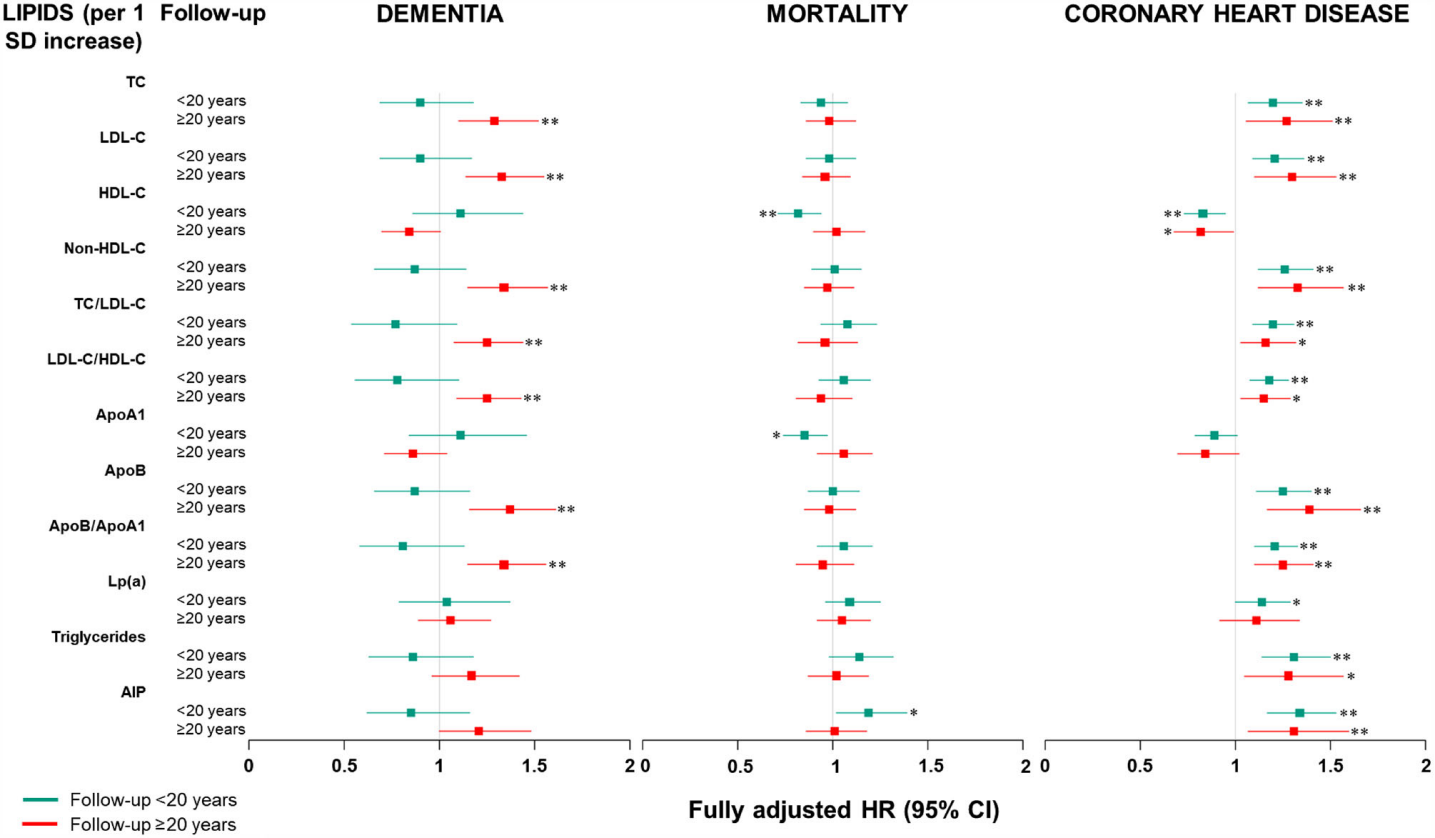
Association of lipids (1991 to 1993) with dementia, mortality, and coronary heart disease (CHD) over the follow‐up (until March 2019) stratified by the length of follow‐up in women. For follow‐up of <20 years; *N* of cases/total = 54/2,366 for dementia, 222/2,366 for mortality, and 263/2,366 for CHD. In the follow‐up of ≥20 years the corresponding numbers for dementia were 126/2,109, for mortality 216/2,144, and for CHD 119/1,891. Model adjusted for age (as time scale), ethnicity, marital status, education, socioeconomic position, use of lipids‐lowering drugs, and health‐related behaviors (smoking, alcohol consumption, physical activity, diet, and obesity), allowing the baseline hazard to differ by birth‐cohort (5‐year groups). Adjustment of the *P*‐values after correction for multiple testing with false discovery rate analysis did not change the conclusions. Abbreviations: AIP, atherogenic index of plasma; ApoA1, apolipoprotein A1; ApoB: apolipoprotein B; CI, confidence interval; HDL‐C, high‐density lipoprotein cholesterol; HR, hazard ratio; LDL‐C, low‐density lipoprotein cholesterol; Lp(a), lipoprotein A; TC, total cholesterol. *P < 0.05, **P < 0.01.

Figure 2 and Table S8 show the corresponding analyses in women. In the multivariable adjusted analyses with a follow‐up of ≥20 years, TC (hazard ratio [HR] = 1.29; 95% confidence interval [CI] 1.10 to 1.52), LDL‐C (HR = 1.33; 95% CI 1.14 to 1.55), non‐HDL‐C (HR = 1.34; 95% CI 1.15 to 1.57), TC/HDL‐C ratio (HR = 1.25; 95% CI 1.08 to 1.44), LDL‐C/HDL‐C ratio (HR = 1.25; 95% CI 1.09 to 1.43), ApoB (HR = 1.37; 95% CI 1.16 to 1.61), and ApoB/ApoA1 ratio (HR = 1.34; 95% CI 1.15 to 1.56) were associated with a higher risk of dementia. In the multivariable adjusted analyses with mortality as the outcome, HDL‐C, ApoA1, and AIP were associated with mortality. Besides ApoA1 and Lp(a) in the ≥20‐year follow‐up, all lipids were associated with risk of CHD in women.

Adjusting for cardiovascular risk factors (hypertension and diabetes) did not substantially change the results in men (Table S9) or women (Table S20). In analyses of participants without *APOE ε*4 alleles, the results were similar to those in the main analyses for men (Table S11) and women (Table S12), apart from an unexpected lower dementia risk found for higher TC, LDL‐C, and non‐HDL‐C for ≥20 year follow‐up in men. Similarly, in further analyses of participants without *APOE ε*2 alleles the associations were contrary to expectations with high levels of TC, LDL‐C, non‐HDL‐C, and ApoB associated with lower risk of dementia in men in the < 20 year follow‐up (Table S13), but results in women were similar to those in the main analyses (Table S14). In analysis with lipids‐lowering drugs treated as a time‐varying covariate, the results did not significantly differ from those in the main analyses in men (Table S15) or women (Table S16). Adjustment for menopausal status in analyses of women did not modify the main findings (Table S17).

### 3.3 Trajectories of lipids using a backward time‐scale

Backward lipid trajectories over 28 years before dementia, mortality, and CHD for men and women are shown in Figure 3; the accompanying differences in lipids between cases and non‐cases at time 0 and 5, 10, 15, 20, and 25 years prior to the date of the event are shown for each outcome in Table S18. For dementia in men (Figure 3), TC (*P =* 0.0267), HDL‐C (*P <* 0.0001), TC/HDL‐C (*P =* 0.0036), LDL‐C/HDL‐C (*P =* 0.0341), triglycerides (*P =* 0.0267), and AIP (*P <* 0.0001) trajectories differed between cases and non‐cases. This reflects increases in HDL‐C and declines in triglycerides and AIP in dementia cases in the 1 to 5 years before dementia diagnosis. In women, trajectories of LDL‐C (*P =* 0.0025), non‐HDL‐C (*P =* 0.0041), and TC/HDL‐C (*P =* 0.0350) differed between dementia cases and non‐cases with higher levels in midlife and progressive decline among dementia cases resulting in similar levels between both groups at dementia diagnosis. Additional adjustment for menopausal status in women did not change the findings (Figure S2).

FIGURE 3Trajectories of lipids over 28 years before dementia, mortality, and coronary heart disease in men and women using a backward timescale. Estimated marginal means based on linear mixed models with a backward scale of time (time before occurrence of the event, in years, with t = 0 defined as the time of the event or March 31, 2019, whichever came first). The analyses were adjusted for sex, use of lipids‐lowering drugs (time‐varying), ethnicity, marital status (time‐varying), education, socioeconomic position (time‐varying), health‐related behaviors (smoking status, alcohol consumption, fruit and vegetable consumption, physical activity, all time‐varying), obesity (time‐varying), a binary indicator of data from 1991 to 1993 (pre‐statin era), age at t = 0, and event status at t = 0. These models included an interaction term between time and event status at t = 0 as well as between time and age at t = 0. Estimations are reported for an average individual aged 75 years old at time t = 0. Italics indicate that *p*‐values were no longer significant after correction for multiple testing with False Discovery Rate analyses. Segments of dotted line superimposed over the time ranges marked for *P* < 0.05 indicate portions for which the *P*‐values were no longer significant after correction for multiple testing with false discovery rate (FDR) analysis. Abbreviations: AIP, atherogenic index of plasma; HDL‐C, high‐density lipoprotein cholesterol; LDL‐C: low‐density lipoprotein cholesterol; TC: total cholesterol.

**Figure:**
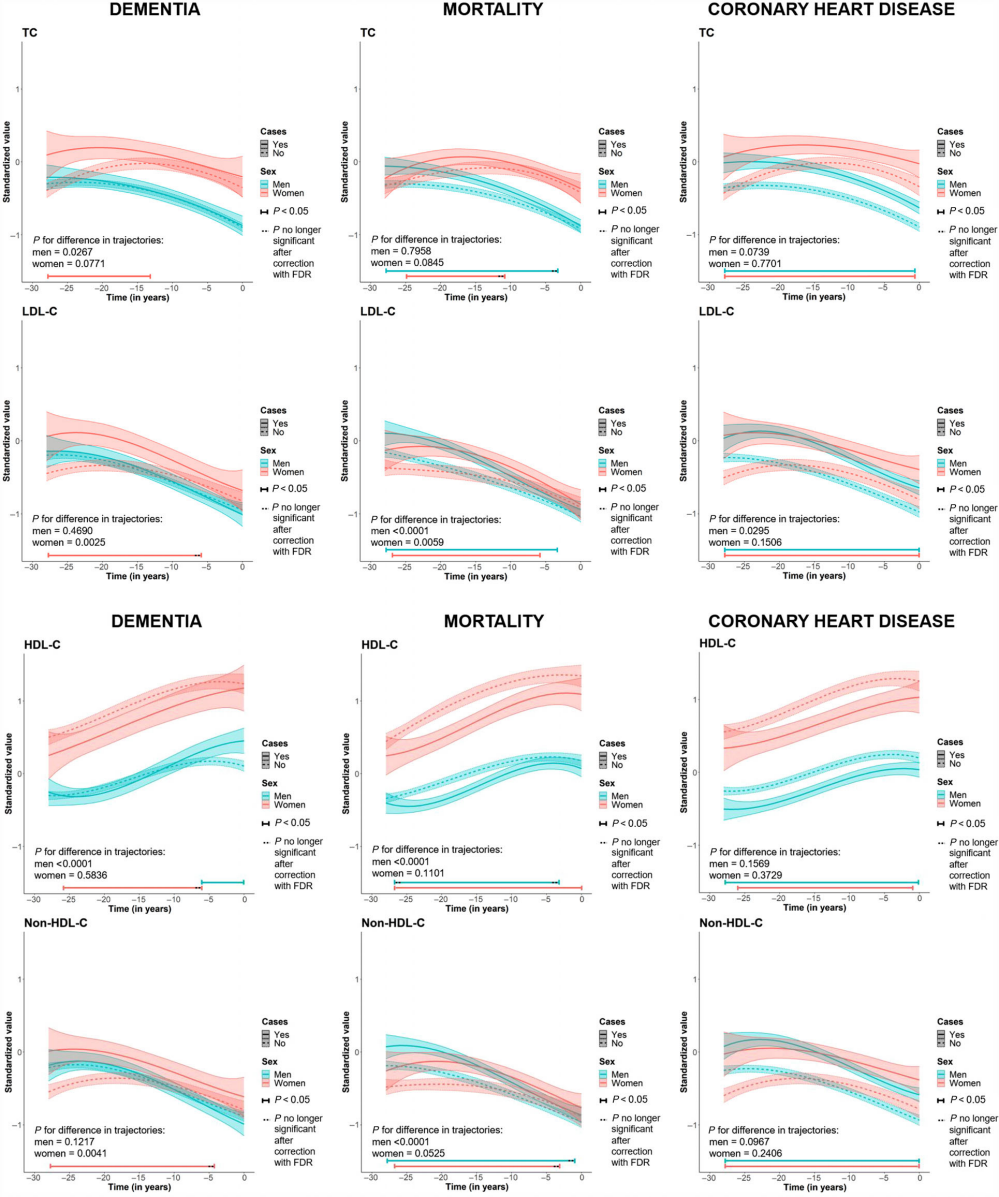

**Figure:**
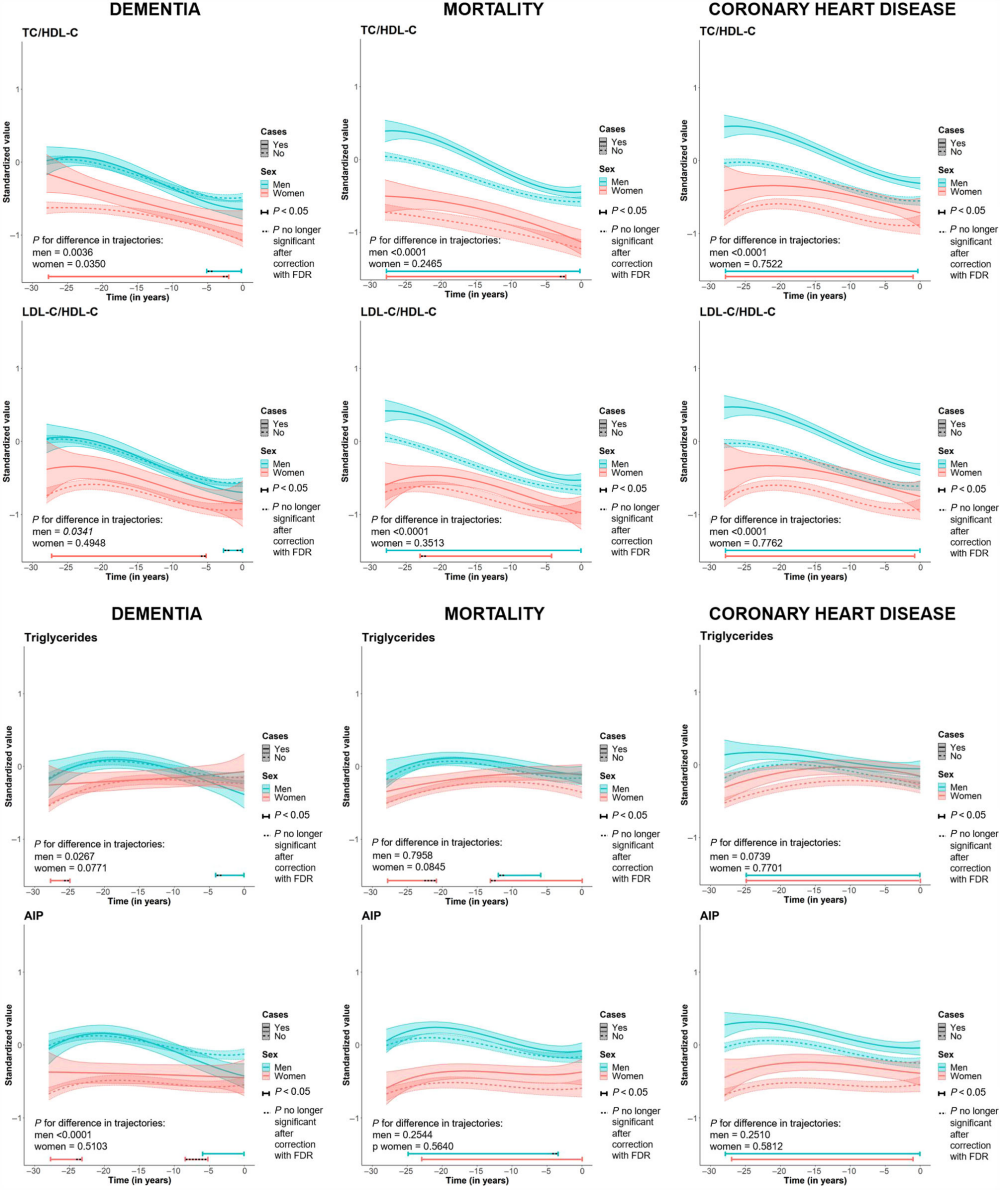

Differences in lipid levels between cases and non‐cases for mortality and CHD were observed in both men and women (Figure 3 and Table S18), and were characterized by an adverse profile in midlife. The decline in lipid levels before the event was more marked for mortality than CHD as is evident in the *P*‐value for differences in trajectories between cases and non‐cases in Figure 3.

## 4 DISCUSSION

Our results show the complex relationship between serum lipids and dementia, with sex and length of follow‐up important modifiers of this association. Lipids were consistently associated with dementia only in women, with the associations in the time‐to‐event analyses being restricted to a follow‐up longer than 20 years. Backward‐timescale trajectories anchored to date of diagnosis confirmed this pattern of results. For the eight lipid markers with repeated data spanning 28 years, the differences between dementia cases and non‐cases in women were greatest long before diagnosis, diminished over time, and were completely attenuated at diagnosis. Such a pattern was not observed in men. The present study adds to current evidence by using both time‐to‐event and trajectories analyses. Confidence in our findings on dementia stems from the use of two other outcomes where the associations of lipids with mortality and CHD in men and women were similar to those reported in other studies, for example the robust association of lipids with acute myocardial infarction^6^ and mortality^43^ in the multinational INTERHEART study.

Identification of risk factors for diseases with a long preclinical phase such as dementia is challenging as disease onset cannot be pinpointed with precision, and results from studies with a short follow‐up are likely to be subject to bias, primarily due to reverse causation. The association of serum lipids (primarily TC, LDL‐C, HDL‐C, and triglycerides) with AD/dementia has been examined in several studies with inconsistent results. TC measured in midlife but not at older ages has been reported to be associated with risk of dementia in several studies.^9^, ^10^, ^12^ However, it remains unclear if the timing of exposure (before or after 65 years) is more relevant than the length of follow‐up and the pathophysiological phase. In the 3‐C Study (mean age at baseline: 75), TC and LDL‐C were not associated with dementia at the 7‐year follow‐up^44^ but there was an association at the 13‐year follow‐up.^26^ A recent study on 1.8 million individuals from a primary care database found TC and LDL‐C but not HDL‐C and triglycerides to be associated with dementia when lipids were measured before 65 years and the follow‐up was longer than 10 years, in analyses with sex as a covariate.^14^ A recent study of 469,466 participants (median follow‐up 11.8 years) showed ApoA1, ApoB, their ratio, and triglycerides to be associated with the risk of dementia in analyses adjusted for sex, with association being stronger before age 60.^45^ Studies that have examined the prospective association of HDL‐C did not find an association with dementia.^14^, ^34^

Our panel of lipids is based on that used in the INTERHEART study^6^ where the ApoB/ApoA1 ratio was a stronger risk factor than other cholesterol ratios for acute myocardial infarction. In our analyses the ApoB/ApoA1 ratio was associated with higher risk of CHD, irrespective of the length of follow‐up. In the FINRISK study, ApoA1, ApoB, and their ratio were not associated with dementia in a follow‐up of 10 years,^34^ again in analyses not stratified by sex. We found both ApoB and the ApoB/ApoA1 ratio to be associated with higher risk of dementia only in women, and only when the follow‐up was over 20 years. Findings on the role of LDL‐C for risk in AD from Mendelian randomization analyses using sex as a covariate are inconsistent; some studies report a non‐significant beneficial impact,^27^ no association,^46^ or a significant adverse effect,^47^ but this approach has not yet been used for other lipids with AD or dementia as the outcome. Systematic reviews of trials reported some evidence that statin use in older adults at risk of vascular disease does not prevent dementia, but these trials have a short follow‐up.^31^, ^48^

For cognitive function there is some evidence of greater vulnerability in women to the effect of vascular risk factors^20^, ^21^ and *APOE ε*4^22^; for *APOE ε*4 this also extends to risk of AD.^23^, ^24^, ^25^ Few studies have tested sex differences in the association of lipids with dementia, although most studies include sex as a covariate. Our data show striking differences between men and women in the association between lipids and dementia. These differences were seen in the survival analyses using baseline data on lipids but also in change in levels over the course of 28 years prior to diagnosis. In the Honolulu‐Asia Aging Study on men where TC was measured five times over 26 years, midlife levels in men who later developed dementia were no different from those who remained dementia‐free,^17^ as was the case in our study. The Swedish Prospective Population Study of Women on 161 dementia cases in 1,462 women, followed for 32 years, reported no association between baseline high cholesterol (>6.5 mmol/L) and dementia (HR 1.27; 95% CI 0.89 to 1.89),^18^ although the effect size was similar to that in our study. In both these studies declining cholesterol between midlife and older ages was associated with a higher risk of dementia, although the time between the measure of the exposure (decline in lipids) and diagnosis varies across individuals in this approach as the follow‐up is not anchored to date of event. A recent systematic review of studies on the association of trajectory of change in cholesterol with incident dementia found only three studies^17^, ^18^, ^49^ with more than two measures over time and concluded that the results were inconsistent although there was a suggestion of greater decline in cholesterol levels in later life in those who developed dementia.^50^ The role of sex was not considered in the review.

The pathways through which lipids affect dementia and the explanation for sex differences remain to be elucidated. Much of the research on AD and related dementias focuses on central nervous system (CNS) biomarkers but several peripheral and systemic abnormalities are also thought to play a role, primarily due to dysfunction of the blood‐brain barrier.^1^, ^2^, ^3^ A recent study did not find associations between midlife lipids (TC, HDL‐C, LDL‐C, and triglycerides) or change in lipids from mid‐ to late life and elevated amyloid burden in late life, suggesting that pathways other than amyloid deposition may be involved in the association between lipids and dementia.^51^ The higher risk of dementia in women is likely to be due to hormonal, genetic, and lifestyle‐induced sex‐differences.^52^, ^53^ The drop in estrogen levels after menopause is thought to be involved through an estrogen receptor and apoE‐mediated pathway^52^, ^54^ and increase in cardiovascular diseases post‐menopause.^52^, ^53^ Our analyses do not show menopausal status to alter results. These explanations need to be substantiated in future studies.

Serum cholesterol and its lipoprotein carriers are known to be related to atherosclerotic cardiovascular disease, with ApoB thought to be a stronger indicator of atherogenicity than LDL‐C alone^55^ and it is promoted as the primary marker to assess cardiovascular risk.^32^ Thus, it is possible that cerebrovascular disease explains part of the association between lipids and dementia. Mendelian randomization studies suggest LDL‐C, triglycerides, ApoB, HDL‐C, and ApoA1 have a causal association with CHD.^33^ A similar approach has been used to show ApoB, LDL‐C, and triglycerides to be associated with ischemic stroke, large artery stroke, and small vessel stroke.^56^ Genome‐wide association studies show several genes, the most important of them being *APOE*, to be involved in lipid metabolism associated with AD.^57^ *APOE* is thought to be involved in other processes such as amyloid beta metabolism, CNS lipid homeostasis, synaptic activity, response to cellular injury, and neuroinflammation.^11^, ^58^, ^59^ However, confining analyses to non‐carriers of the *APOE ε*4 allele did not alter conclusions in women in our study. Rather surprisingly, analysis in men restricted to non‐*APOE ε*4 or non‐*APOE ε*2 carriers suggested a protective association of elevated TC, LDL‐C, non‐HDL‐C (and ApoB for non‐*APOE ε*2 carriers) with dementia but this was not the case for mortality or CHD, where results were in the expected direction.

The primary strength of the present study Is repeated data on lipids spanning a median of 26.8 years. The study was sufficiently large to allow the examination of sex differences. The fact that data on outcomes were available on all participants through linkage to electronic health records is a further strength as selection over the course of the study can bias results. Results from both time‐to‐event as well as trajectory analyses, along with three outcomes (dementia, mortality, and CHD) strengthen the robustness of our findings. Our results must also be considered in light of some limitations. As with all longitudinal cohort studies the participants of the Whitehall II study are healthier than the general population but we have previously shown this not to affect the association between risk factors and disease.^60^ A further limitation is that the study population has more men than women, and this is likely to lead to smaller differences being statistically significant in men. In addition, incomplete data on dementia subtype made it impossible to examine whether the results differed as a function of this factor.

In conclusion, our study on the association of lipids with dementia using time‐to‐event analyses and trajectories up to diagnosis suggests sex differences that were not seen to the same extent for mortality and CHD. The specificity of the association between lipids and dementia requires replication and further research to elucidate underlying mechanisms.

## AUTHOR CONTRIBUTIONS

Céline Ben Hassen, Séverine Sabia, and Archana Singh‐Manoux generated the hypothesis and designed the study. Céline Ben Hassen, Aurore Fayosse, Séverine Sabia, and Archana Singh‐Manoux developed the study methods. Aurore Fayosse and Céline Ben Hassen curated the data. Céline Ben Hassen did the formal analyses. Céline Ben Hassen, Séverine Sabia, Benjamin Landré, and Archana Singh‐Manoux visualized and interpreted the data. Céline Ben Hassen and Archana Singh‐Manoux wrote the original draft of the manuscript. All authors discussed the results and commented on the manuscript. Séverine Sabia and Archana Singh‐Manoux supervised the project. Archana Singh‐Manoux acquired funding. All authors had full access to all the data in the study and accept responsibility in submitting for publication. Céline Ben Hassen is the guarantor. The corresponding author attests that all listed authors meet authorship criteria and that no others meeting the criteria have been omitted.

## CONFLICT OF INTEREST STATEMENT

We have no conflict of interests to declare. Author disclosures are available in the supporting information

## CONSENT STATEMENTS

All participants provided informed consent.

## Supporting information

Supplementary information

Supplementary information
