# Supplementary material for: Change in lipids before onset of dementia, coronary heart disease, and mortality: A 28‐year follow‐up Whitehall II prospective cohort study
Source: Alzheimers Dement. 2023 May 27;19(12):5518–30. doi: 10.1002/alz.13140 (PMC10679471; doi:10.1002/alz.13140)
Supplement: Supplementary file 1 — Supplementary information [file ALZ-19-5518-s001.docx]

**Change in lipids before onset of dementia, coronary heart disease, and mortality: 28-year follow-up Whitehall II prospective cohort study**

Céline Ben Hassen (0000-0001-7884-2986), PhD^1^*, Marcos D Machado-Fragua, PhD^1^, Benjamin Landré, PhD^1^, Aurore Fayosse, MSc^1^; Julien Dumurgier, PhD^1,2^; Mika Kivimaki, PhD^3^; Severine Sabia, PhD^,3^; Archana Singh-Manoux, PhD^1,3^

^1^Université Paris Cité, Inserm U1153, Epidemiology of Ageing and Neurodegenerative diseases, 75010 Paris, France

^2^Cognitive Neurology Center, Lariboisière – Fernand Widal Hospital, AP-HP, Université Paris Cité, 75010 Paris, France

^3^Department of Mental Health of Older People, Faculty of Brain Sciences, University College London, London W1T 7NF, UK

**Supplemental data**

eTable 1. Characteristics of participants at baseline (1991-1993) according to mortality status at the end of the follow-up.

eTable 2. Characteristics of participants at baseline (1991-1993) according to coronary heart disease status at the end of the follow-up.

eTable 3. Lipid levels at baseline (1991-1993) according to mortality status at the end of the follow-up.

eTable 4. Lipids levels at baseline (1991-1993) according to coronary heart disease status at the end of the follow-up.

eTable 5. Association of blood lipids (1991-1993) with incidence of dementia, mortality, and coronary heart disease stratified by the length of follow-up.

eTable 6. The *P* value for interaction between sex and lipids in the association of lipids with dementia, mortality, and coronary heart disease in analysis stratified by the length of follow-up.

eTable 7. Association of lipids (1991-1993) with dementia, mortality, and coronary heart disease over the follow-up (until March 2019) stratified by the length of follow-up in men.

eTable 8. Association of lipids (1991-1993) with dementia, mortality, and coronary heart disease over the follow-up (until March 2019) stratified by the length of follow-up in women.

eTable 9. Association of lipids (1991-1993) with dementia, mortality, and coronary heart disease over the follow-up (until March 2019) stratified by the length of follow-up in men and adjusting for cardiovascular risk factors.^a^

eTable 10. Association of lipids (1991-1993) with dementia, mortality, and coronary heart disease over the follow-up (until March 2019) stratified by the length of follow-up in women and adjusting for cardiovascular risk factors.^a,b^

eTable 11. Association of lipids (1991-1993) with dementia, mortality, and coronary heart disease over the follow-up (until March 2019) in men without APOE ε4 allele(s).

eTable 12. Association of lipids (1991-1993) with dementia, mortality, and coronary heart disease over the follow-up (until March 2019) in women without APOE ε4 allele(s).

eTable 13. Association of lipids (1991-1993) with dementia, mortality, and coronary heart disease over the follow-up (until March 2019) in men without APOE ε2 allele(s).

eTable 14. Association of lipids (1991-1993) with dementia, mortality, and coronary heart disease over the follow-up (until March 2019) in women without APOE ε2 allele(s).

eTable 15. Association of lipids (1991-1993) with dementia, mortality, and coronary heart disease over the follow-up (until March 2019) in men, adjusting for use of lipids-lowering drugs as a time-varying measure.

eTable 16. Association of lipids (1991-1993) with dementia, mortality, and coronary heart disease over the follow-up (until March 2019) in women, adjusting for use of lipids-lowering drugs as a time-varying measure.

eTable 17. Estimated marginal mean (cases – non-cases) trajectories of blood lipids over 28 years before dementia, mortality, and coronary heart disease in men and women using a backward timescale.

eTable 18. Association of lipids (1991-1993) with dementia, mortality, and coronary heart disease over the follow-up (until March 2019) stratified by the length of follow-up in women adjusting for menopausal status.

eFigure 1. Flowchart

eFigure 2. Trajectories of blood lipids over 28 years before dementia, mortality, and coronary heart disease women using a backward timescale and adjusting for menopausal status.

STROBE checklist statement

**eTable 1. Characteristics of participants at baseline (1991-1993) according to mortality status at the end of the follow-up.**

|  | **Men** | | | | **Women** | | | |
| --- | --- | --- | --- | --- | --- | --- | --- | --- |
| **Participant characteristics** | **Mortality status (March 2019)** | | | | **Mortality status (March 2019)** | | | |
|  | **Deceased** | **Alive** | ***p* value** | **Deceased** | | **Alive** | ***p* value** |  |
| **N** | **N=952** | **N=4354** |  | **N=438** | | **N=1928** |  |  |
| Age, M (SD) | 53.5 (5.9) | 48.9 (5.7) | <0.0001 | 54.0 (5.5) | | 49.8 (5.9) | <0.0001 |  |
| Education Lower secondary school or lower | 432 (45.4) | 1636 (37.6) | <0.0001 | 299 (0.68) | | 1123 (58.2) | <0.0001 |  |
| Secondary school | 272 (28.6) | 1271 (29.2) |  | 91 (20.8) | | 411 (21.3) |  |  |
| University degree or higher | 248 (26.1) | 1447 (33.2) |  | 48 (11.0) | | 394 (20.4) |  |  |
| Socioeconomic position 1 (high) | 224 (23.5) | 963 (22.1) | <0.0001 | 15 (3.4) | | 133 (6.9) | <0.0001 |  |
| 2 | 216 (22.7) | 1170 (26.9) |  | 38 (8.7) | | 200 (10.4) |  |  |
| 3 | 138 (14.5) | 764 (17.5) |  | 17 (3.9) | | 168 (8.7) |  |  |
| 4 | 164 (17.2) | 788 (18.1) |  | 58 (13.2) | | 296 (15.4) |  |  |
| 5 | 111 (11.7) | 432 (9.9) |  | 113 (25.8) | | 420 (21.8) |  |  |
| 6 (low) | 99 (10.4) | 237 (5.4) |  | 197 (45.0) | | 711 (36.9) |  |  |
| Ethnicity White | 862 (90.5) | 4067 (93.4) | 0.0023 | 372 (84.9) | | 1655 (85.8) | 0.6785 |  |
| Non-White | 90 (9.5) | 287 (6.7) |  | 66 (15.1) | | 273 (14.2) |  |  |
| Marital status Married/Cohabiting | 765 (80.4) | 3613 (83.0) | 0.0596 | 258 (58.9) | | 1227 (63.6) | 0.0724 |  |
| Single/Divorced/Widowed | 187 (19.6) | 741 (17.0) |  | 180 (41.1) | | 701 (36.4) |  |  |
| Use of lipid-lowering drugs | 28 (0.6) | 9 (0.9) | 0.4235 | 4 (0.9) | | 9 (0.5) | 0.4337 |  |
| **Health behaviours** |  |  |  |  | |  |  |  |
| Smoking Never smoker | 393 (41.3) | 2173 (49.9) | <0.0001 | 192 (43.8) | | 1107 (57.4) | <0.0001 |  |
| Former smoker | 385 (40.4) | 1709 (39.3) |  | 135 (30.8) | | 549 (28.5) |  |  |
| Current smoker | 174 (18.3) | 472 (10.8) |  | 111 (25.3) | | 272 (14.1) |  |  |
| Hours of MVPA per week, M (SD) | 3.96 (4.22) | 3.90 (3.83) | 0.6903 | 2.38 (4.14) | | 2.62 (4.04) | 0.2682 |  |
| Alcohol consumption 0 unit/week | 168 (17.6) | 596 (13.7) | 0.0006 | 149 (34.0) | | 567 (29.4) | 0.1588 |  |
| 1-14 units/week | 478 (50.2) | 2449 (56.2) |  | 248 (56.6) | | 1177 (61.0) |  |  |
| > 14 units/week | 306 (32.1) | 1309 (30.1) |  | 41 (9.4) | | 184 (9.5) |  |  |
| Fruit/vegetable consumption < Once/day | 424 (44.5) | 1742 (40.0) | 0.0111 | 162 (37.0) | | 639 (33.1) | 0.1393 |  |
| ≥ Once/day | 528 (39.6) | 2612 (60.0) |  | 276 (63.0) | | 1289 (66.9) |  |  |
| Obesity | 95 (10.0) | 264 (6.1) | <0.0001 | 103 (23.5) | | 259 (13.4) | <0.0001 |  |

Abbreviations: M, Mean; MVPA, Moderate and Vigorous Physical Activity; SD, Standard Deviation.

Data are N (%) unless stated otherwise.

Non-white group was composed of 244 South Asian, 98 Black, and 33 Others among men and 141 South Asian, 163 Black, and 25 Others among women.

**eTable 2. Characteristics of participants at baseline (1991-1993) according to coronary heart disease status at the end of the follow-up.**

|  | **Men** | | | | **Women** | | | |
| --- | --- | --- | --- | --- | --- | --- | --- | --- |
| **Participant characteristics** | **Coronary heart disease status (March 2019)** | | | | **Coronary heart disease status (March 2019)** | | | |
|  | **CHD** | **No CHD** | ***p* value** | **CHD** | | **No CHD** | ***p* value** |  |
| **N** | **N=1,131** | **N=4175** |  | **N=382** | | **N=1,984** |  |  |
| Age, M (SD) | 51.8 (5.9) | 49.1 (5.9) | <0.0001 | 55.3 (5.7) | | 50.1 (6.0) | <0.0001 |  |
| Education Lower secondary school or lower | 509 (45.0) | 1559 (37.3) | <0.0001 | 277 (72.5) | | 1145 (57.7) | <0.0001 |  |
| Secondary school | 333 (29.4) | 1210 (29.0) |  | 60 (15.7) | | 442 (22.3) |  |  |
| University degree or higher | 289 (25.6) | 1406 (33.7) |  | 45 (11.8) | | 397 (20.0) |  |  |
| Socioeconomic position 1 (high) | 237 (21.0) | 950 (22.8) | 0.0010 | 7 (1.8) | | 141 (7.1) | <0.0001 |  |
| 2 | 278 (24.6) | 1108 (26.5) |  | 21 (5.5) | | 217 (10.9) |  |  |
| 3 | 172 (15.2) | 730 (17.5) |  | 22 (5.8) | | 163 (8.2) |  |  |
| 4 | 225 (19.9) | 727 (17.4) |  | 45 (11.8) | | 309 (15.6) |  |  |
| 5 | 134 (11.8) | 409 (9.8) |  | 105 (27.5) | | 428 (21.6) |  |  |
| 6 (low) | 85 (7.5) | 251 (6.0) |  | 182 (47.6) | | 726 (36.6) |  |  |
| Ethnicity White | 1,001 (88.5) | 3928 (94.1) | <0.0001 | 298 (78.0) | | 1729 (87.1) | <0.0001 |  |
| Non-White | 130 (11.5) | 247 (5.9) |  | 84 (22.0) | | 255 (12.9) |  |  |
| Marital status Married/Cohabiting | 960 (84.9) | 3418 (81.9) | 0.0203 | 250 (65.4) | | 1235 (62.2) | 0.2602 |  |
| Single/Divorced/Widowed | 171 (14.1) | 757 (18.1) |  | 132 (35.6) | | 749 (37.8) |  |  |
| Use of lipid-lowering drugs | 11 (1.0) | 26 (0.6) | 0.2925 | 3 (0.8) | | 10 (0.5) | 0.7618 |  |
| **Health behaviours** |  |  |  |  | |  |  |  |
| Smoking Never smoker | 483 (42.7) | 2083 (49.9) |  | 198 (51.8) | | 1101 (55.5) | 0.0337 |  |
| Former smoker | 467 (41.3) | 1627 (39.0) |  | 105 (27.5) | | 579 (29.2) |  |  |
| Current smoker | 181 (16.0) | 465 (11.1) |  | 79 (20.7) | | 304 (15.3) |  |  |
| Hours of MVPA per week, M (SD) | 4.08 (4.18) | 3.86 (3.82) | 0.1163 | 2.30 (3.86) | | 2.63 (4.09) | 0.1315 |  |
| Alcohol consumption 0 unit/week | 187 (16.5) | 577 (13.8) | 0.0506 | 163 (52.7) | | 553 (27.9) | <0.0001 |  |
| 1-14 units/week | 619 (54.7) | 2308 (55.3) |  | 199 (52.1) | | 1226 (61.8) |  |  |
| > 14 units/week | 325 (28.7) | 1290 (30.9) |  | 20 (5.2) | | 205 (10.3) |  |  |
| Fruit/vegetable consumption < Once/day | 471 (41.6) | 1695 (40.6) | 0.5481 | 136 (35.6) | | 665 (33.5) | 0.4659 |  |
| ≥ Once/day | 660 (58.4) | 2480 (59.4) |  | 246 (64.4) | | 1319 (66.5) |  |  |
| Obesity | 112 (9.9) | 247 (5.9) | <0.0001 | 82 (21.5) | | 300 (78.5) | 0.0003 |  |

Abbreviations: M, Mean; MVPA, Moderate and Vigorous Physical Activity; SD, Standard Deviation.

Data are N (%) unless stated otherwise.

Non-white group was composed of 244 South Asian, 98 Black, and 33 Others among men and 141 South Asian, 163 Black, and 25 Others among women.

**eTable 3. Lipid levels at baseline (1991-1993) according to mortality status at the end of the follow-up.**

|  | **Men** | | | | **Women** | | | |
| --- | --- | --- | --- | --- | --- | --- | --- | --- |
| **Participant characteristics** | **Mortality status (March 2019)** | | | | **Mortality status (March 2019)** | | | |
|  | **Deceased** | **Alive** | ***p* value** | **Deceased** | | **Alive** | ***p* value** |  |
| **N** | **N=952** | **N=4354** |  | **N=438** | | **N=1928** |  |  |
| **Lipids, M (SD)** |  |  |  |  | |  |  |  |
| Total Cholesterol, mmol/l | 6.60 (1.12) | 6.41 (1.10) | <0.0001 | 6.72 (1.58) | | 6.44 (1.18) | <0.0001 |  |
| LDL-Cholesterol, mmol/l | 4.55 (1.01) | 4.41 (0.99) | <0.0001 | 4.48 (1.16) | | 4.23 (1.09) | <0.0001 |  |
| HDL-Cholesterol, mmol/l | 1.30 (0.37) | 1.34 (0.34) | 0.0071 | 1.63 (0.42) | | 1.70 (0.43) | <0.0001 |  |
| Non-HDL-Cholesterol, mmol/l | 5.29 (1.14) | 5.07 (1.13) | <0.0001 | 5.10 (1.32) | | 4.74 (1.22) | 0.0013 |  |
| Total Cholesterol / HDL-Cholesterol | 5.44 (1.71) | 5.09 (1.53) | <0.0001 | 4.41 (1.44) | | 4.05 (1.46) | <0.0001 |  |
| LDL-Cholesterol / HDL-Cholesterol | 3.79 (1.38) | 3.52 (1.22) | <0.0001 | 2.97 (1.18) | | 2.70 (1.25) | <0.0001 |  |
| Apolipoprotein A1 (ApoA1), mmol/l | 2.05 (0.35)  2.06  .3) | 2.06 (0.31) | 0.3527 | 2.31 (0.37) | | 2.33 (0.38) | 0.2344 |  |
| Apolipoprotein B (ApoB), mmol/l | 1.35 (0.30) | 1.29 (0.29) | <0.0001 | 1.28 (0.32) | | 1.20 (0.30) | <0.0001 |  |
| ApoB / ApoA1 | 0.68 (0.21) | 0.64 (0.18) | <0.0001 | 0.57 (0.17) | | 0.53 (0.18) | <0.0001 |  |
| Lipoprotein(a) (log) | 3.15 (0.87) | 3.07 (0.89) | 0.0086 | 3.28 (0.94) | | 3.14 (0.90) | 0.0049 |  |
| Triglycerides (log) | 0.37 (0.49) | 0.24 (0.51) | <0.0001 | 0.18 (0.50) | | 0.01 (0.47) | <0.0001 |  |
| Atherogenic Index of Plasma (AIP) | 0.14 (0.67) | -0.02 (0.68) | <0.0001 | -0.27 (0.67) | | -0.49 (0.63) | <0.0001 |  |

Abbreviations: LDL, Low-Density Lipoprotein; HDL, High-Density Lipoprotein.

**eTable 4. Lipids levels at baseline (1991-1993) according to coronary heart disease status at the end of the follow-up.**

|  | **Men** | | | | **Women** | | | |
| --- | --- | --- | --- | --- | --- | --- | --- | --- |
| **Participant characteristics** | **Coronary heart disease status (March 2019)** | | | | **Coronary heart disease status (March 2019)** | | | |
|  | **CHD** | **No CHD** | ***p* value** | **CHD** | | **No CHD** | ***p* value** |  |
| **N** | **N=1131** | **N=4175** |  | **N=382** | | **N=1984** |  |  |
| **Lipids, M (SD)** |  |  |  |  | |  |  |  |
| Total Cholesterol, mmol/l | 6.70 (1.05) | 6.37 (1.11) | <0.0001 | 6.89 (1.30) | | 6.42 (1.17) | <0.0001 |  |
| LDL-Cholesterol, mmol/l | 4.67 (0.98) | 4.37 (0.99) | <0.0001 | 4.67 (1.19) | | 4.20 (1.08) | <0.0001 |  |
| HDL-Cholesterol, mmol/l | 1.26 (0.33) | 1.35 (0.35)  35 ( | <0.0001 | 1.58 (0.43) | | 1.70 (0.42) | <0.0001 |  |
| Non-HDL-Cholesterol, mmol/l | 5.44 (1.09) | 5.02 (1.13) | <0.0001 | 5.31 (1.32) | | 4.71 (1.21) | <0.0001 |  |
| Total Cholesterol / HDL-Cholesterol | 5.65 (1.68) | 5.01 (1.50) | <0.0001 | 4.65 (1.49) | | 4.01 (1.44) | <0.0001 |  |
| LDL-Cholesterol / HDL-Cholesterol | 3.96 (2.35) | 3.47 (1.21) | <0.0001 | 3.19 (1.26) | | 2.67 (1.22) | <0.0001 |  |
| Apolipoprotein A1 (ApoA1), mmol/l | 2.02 (0.32) | 2.07 (0.32)  0 | <0.0001 | 2.27 (0.37) | | 2.34 (0.38) | <0.0001 |  |
| Apolipoprotein B (ApoB), mmol/l | 1.39 (0.28) | 1.28 (0.29) | <0.0001 | 1.34 (0.32) | | 1.19 (0.29) | <0.0001 |  |
| ApoB / ApoA1 | 0.71 (0.19) | 0.63 (0.18) | <0.0001 | 0.61 (0.19) | | 0.52 (0.17) | <0.0001 |  |
| Lipoprotein(a) (log) | 3.21 (0.89) | 3.05 (0.88) | <0.0001 | 3.34 (0.94) | | 3.13 (0.90) | <0.0001 |  |
| Triglycerides (log) | 0.39 (0.50) | 0.23 (0.50) | <0.0001 | 0.22 (0.48) | | 0.00 (0.48) | <0.0001 |  |
| Atherogenic Index of Plasma (AIP) | 0.19 (0.67) | -0.04 (0.67) | <0.0001 | -0.20 (0.65) | | -0.50 (0.63) | <0.0001 |  |

Abbreviations: LDL, Low-Density Lipoprotein; HDL, High-Density Lipoprotein.

**eTable 5. Association of lipids (1991-1993) with incidence of dementia, mortality, and coronary heart disease stratified by the length of follow-up.^a,b^**

|  |  | **Dementia** | |  | **Mortality** | |  | **Coronary Heart Disease** | |
| --- | --- | --- | --- | --- | --- | --- | --- | --- | --- |
| **LIPIDS (per 1 SD increase)** | **Length of follow-up** | **Model 1**  **HR (95% CI)** | **Model 2**  **HR (95% CI)** |  | **Model 1**  **HR (95% CI)** | **Model 2**  **HR (95% CI)** |  | **Model 1**  **HR (95% CI)** | **Model 2**  **HR (95% CI)** |
| **TC** | < 20 years | 0.85 (0.71–1.02) | 0.85 (0.70–1.01) |  | *1.04 (0.97–1.13)* | *1.02 (0.95–1.10)* |  | *1.25 (1.18–1.32)** | *1.25 (1.18–1.32)** |
|  | ≥ 20 years | 1.06 (0.95–1.19) | 1.06 (0.94–1.18) |  | 0.99 (0.92–1.07) | 0.98 (0.90–1.06) |  | 1.20 (1.09–1.32)* | 1.19 (1.08–1.31)* |
| **LDL-C** | < 20 years | 0.85 (0.71–1.01) | 0.84 (0.71–1.01) |  | *1.04 (0.97–1.11)* | *1.02 (0.95–1.10)* |  | *1.27 (1.20–1.34)** | *1.26 (1.19–1.34)** |
|  | ≥ 20 years | 1.07 (0.96–1.19) | 1.06 (0.95–1.18) |  | 0.98 (0.91–1.06) | 0.97 (0.89–1.05) |  | 1.21 (1.10–1.33)* | 1.21 (1.10–1.33)* |
| **HDL-C** | < 20 years | 1.01 (0.85–1.21) | 1.04 (0.86–1.21) |  | 0.81 (0.74–0.88) | 0.84 (0.77–0.92)* |  | *0.74 (0.69–0.80)** | *0.77 (0.72–0.83)** |
|  | ≥ 20 years | 0.89 (0.79–1.01) | 0.91 (0.81–1.03) |  | 0.93 (0.86–1.02) | 0.97 (0.89–1.06) |  | *0.78 (0.69–0.87)** | *0.78 (0.69–0.88)** |
| **Non-HDL-C** | < 20 years | 0.85 (0.71–1.02) | 0.84 (0.70–1.01) |  | *1.11 (1.03–1.19)** | *1.07 (0.99–1.15)* |  | *1.34 (1.26–1.42)** | *1.32 (1.24–1.40)** |
|  | ≥ 20 years | 1.10 (0.99–1.23) | 1.08 (0.97–1.21) |  | 1.01 (0.94–1.10) | 0.99 (0.91–1.07) |  | 1.28 (1.17–1.41)* | 1.26 (1.15–1.39)* |
| **TC / HDL-C** | < 20 years | 0.86 (0.70–1.05) | 0.84 (0.68–1.03) |  | 1.21 (1.14–1.29)* | 1.17 (1.09–1.25)* |  | 1.32 (1.27–1.38)* | 1.30 (1.24–1.36)* |
|  | ≥ 20 years | 1.11 (0.996–1.24) | 1.09 (0.97–1.22) |  | 1.07 (1.09–1.16)* | 1.03 (0.95–1.12) |  | 1.25 (1.16–1.34)* | 1.23 (1.14–1.32)* |
| **LDL-C / HDL-C** | < 20 years | 0.85 (0.69–1.03) | 0.83 (0.67–1.01) |  | 1.19 (1.11–1.26)* | 1.15 (1.07–1.23)* |  | 1.30 (1.25–1.35)* | 1.27 (1.22–1.33)* |
|  | ≥ 20 years | 1.10 (0.98–1.23) | 1.08 (0.96–1.21) |  | 1.05 (0.97–1.13) | 1.01 (0.93–1.10) |  | 1.22 (1.14–1.31)* | 1.20 (1.12–1.29)* |
| **ApoA1** | < 20 years | 0.99 (0.82–1.19) | 1.00 (0.83–1.21) |  | 0.87 (0.80–0.94)* | 0.89 (0.81–0.96)* |  | 0.82 (0.76–0.87)* | 0.85 (0.79–0.91)* |
|  | ≥ 20 years | 0.92 (0.81–1.03) | 0.93 (0.82–1.05) |  | 0.96 (0.88–1.04) | 0.98 (0.89–1.06) |  | 0.86 (0.77–0.96)* | 0.86 (0.77–0.96)* |
| **ApoB** | < 20 years | 0.87 (0.73–1.05) | 0.86 (0.72–1.03) |  | *1.14 (1.06–1.22)** | 1.09 (1.01–1.17)* |  | *1.34 (1.26–1.42)** | *1.32 (1.24–1.40)** |
|  | ≥ 20 years | 1.14 (1.02–1.27)* | 1.12 (0.992–1.25) |  | 1.02 (0.94–1.11) | 0.92 (0.91–1.07) |  | 1.29 (1.18–1.42)* | 1.27 (1.16–1.40)* |
| **ApoB / ApoA1** | < 20 years | 0.89 (0.73–1.07) | 0.87 (0.71–1.06) |  | 1.19 (1.12–1.27)* | 1.15 (1.07–1.23)* |  | 1.32 (1.26–1.37)* | 1.29 (1.23–1.35)* |
|  | ≥ 20 years | 1.15 (1.03–1.29)* | 1.13 (1.01–1.27)* |  | 1.07 (0.99–1.17) | 1.04 (0.95–1.13) |  | 1.27 (1.18–1.37)* | 1.25 (1.16–1.35)* |
| **Lp(a)** | < 20 years | 1.06 (0.89–1.26) | 1.06 (0.88–1.26) |  | 1.07 (0.99–1.15) | 1.07 (0.99–1.15) |  | 1.17 (1.11–1.25)* | 1.18 (1.11–1.25)* |
|  | ≥ 20 years | 1.03 (0.92–1.15) | 1.02 (0.92–1.14) |  | 1.04 (0.97–1.13) | 1.04 (0.97–1.13) |  | 1.08 (0.98–1.19) | 1.08 (0.98–1.19) |
| **Triglycerides** | < 20 years | 0.99 (0.83–1.19) | 0.97 (0.81–1.17) |  | 1.29 (1.19–1.39)* | 1.20 (1.11–1.30)* |  | *1.34 (1.26–1.42)** | *1.29 (1.22–1.38)** |
|  | ≥ 20 years | *1.12 (1.005–1.26)** | *1.08 (0.96–1.22)* |  | 1.11 (1.03–1.21)* | 1.05 (0.97–1.14) |  | 1.34 (1.22–1.48)* | 1.30 (1.18–1.44)* |
| **AIP** | < 20 years | 0.99 (0.83–1.19) | 0.97 (0.80–1.17) |  | 1.31 (1.22–1.42)* | 1.23 (1.14–1.33)* |  | *1.39 (1.30–1.48)** | *1.34 (1.25–1.43)** |
|  | ≥ 20 years | 1.14 (1.02–1.28)* | 1.10 (0.98–1.23) |  | 1.12 (1.03–1.21)* | 1.06 (0.97–1.15) |  | 1.36 (1.23–1.50)* | 1.33 (1.20–1.47)* |

^a^For follow-up < 20 years, dementia N cases/ N Total=131/7672, 726/7672 for mortality, 1087/7672 for CHD. In the follow-up ≥ 20 years the corresponding numbers for dementia were 331/6867, for mortality 664/6946, and for CHD 426/5979.

^b^Results in italics are from analyses where the proportionality hazards assumption did not hold.

Model 1: adjusted for age (as time scale), ethnicity, marital status, education, socioeconomic position, and use of lipids-lowering drugs, allowing the baseline hazard to differ by birth-cohort (5-years groups).

Model 2: Model 1 + health-related behaviours (smoking, alcohol consumption, physical activity, diet, and obesity).

Abbreviations: TC: Total Cholesterol, LDL-C: Low-Density Lipoprotein cholesterol, HDL-C: High-Density Lipoprotein cholesterol; ApoA1: Apolipoprotein A1; ApoB: Apolipoprotein B; Lp(a): Lipoprotein (a); AIP: Atherogenic Index of Plasma.

**p<0.05*

**eTable 6. The *P* value for interaction between sex and lipids in the association of lipids with dementia, mortality, and coronary heart disease in analysis stratified by the length of follow-up.^a^**

| **LIPIDS (per 1 SD increase)** | **Follow-up** |  | **Dementia**  ***p* for interaction with sex** |  | **Mortality**  ***p* for interaction with sex** |  | **Coronary Heart Disease**  ***p* for interaction with sex** |
| --- | --- | --- | --- | --- | --- | --- | --- |
|  |  |  |  |  |  |  |  |
| **TC** | < 20 years |  | 0.5365 |  | 0.0228* |  | 0.4479 |
|  | ≥ 20 years |  | 0.0037* |  | 0.8413 |  | 0.3680 |
| **LDL-C** | < 20 years |  | 0.5477 |  | 0.0948 |  | 0.5504 |
|  | ≥ 20 years |  | 0.0004* |  | 0.7202 |  | 0.2443 |
| **HDL-C** | < 20 years |  | 0.4230 |  | 0.88033 |  | 0.2559 |
|  | ≥ 20 years |  | 0.0962 |  | 0.3083 |  | 0.7119 |
| **Non-HDL-C** | < 20 years |  | 0.7279 |  | 0.0335* |  | 0.3166 |
|  | ≥ 20 years |  | 0.0008* |  | 0.6131 |  | 0.4090 |
| **TC / HDL-C** | < 20 years |  | 0.5374 |  | 0.0231* |  | 0.0031* |
|  | ≥ 20 years |  | 0.0065* |  | 0.2929 |  | 0.2600 |
| **LDL-C / HDL-C** | < 20 years |  | 0.6575 |  | 0.0220* |  | 0.0008* |
|  | ≥ 20 years |  | 0.0021* |  | 0.2626 |  | 0.2332 |
| **ApoA1** | < 20 years |  | 0.2742 |  | 0.6319 |  | 0.5361 |
|  | ≥ 20 years |  | 0.1360 |  | 0.1643 |  | 0.5930 |
| **ApoB** | < 20 years |  | 0.8479 |  | 0.0097* |  | 0.2959 |
|  | ≥ 20 years |  | 0.0027* |  | 0.6597 |  | 0.2128 |
| **ApoB / ApoA1** | < 20 years |  | 0.6035 |  | 0.0282* |  | 0.0572 |
|  | ≥ 20 years |  | 0.0014* |  | 0.1554 |  | 0.8913 |
| **Lp(a)** | < 20 years |  | 0.9890 |  | 0.9875 |  | 0.7336 |
|  | ≥ 20 years |  | 0.5557 |  | 0.7837 |  | 0.4402 |
| **Triglycerides** | < 20 years |  | 0.3632 |  | 0.0623 |  | 0.8739 |
|  | ≥ 20 years |  | 0.3146 |  | 0.5029 |  | 0.8629 |
| **AIP** | < 20 years |  | 0.3043 |  | 0.1355 |  | 0.8590 |
|  | ≥ 20 years |  | 0.1507 |  | 0.3935 |  | 0.8480 |

^a^From a model with age as time scale, and adjusted for ethnicity, marital status, education, socioeconomic position, use of lipids-lowering drugs, health-related behaviours (smoking, alcohol consumption, physical activity, diet and obesity), and birth-cohort (5-year groups).

TC: Total Cholesterol, LDL-C: Low-Density Lipoprotein cholesterol, HDL-C: High-Density Lipoprotein cholesterol; ApoA1: Apolipoprotein A1; ApoB: Apolipoprotein B; Lp(a): Lipoprotein (a); AIP: Atherogenic Index of Plasma.

**p<0.05*

**eTable 7. Association of lipids (1991-1993) with dementia, mortality, and coronary heart disease over the follow-up (until March 2019) stratified by the length of follow-up in men.^a,b^**

|  |  |  | **Dementia** | |  | **Mortality** | |  | **Coronary Heart Disease** | |
| --- | --- | --- | --- | --- | --- | --- | --- | --- | --- | --- |
| **LIPIDS (per 1 SD increase)** | **Follow-up** |  | **Model 1**  **HR (95% CI)** | **Model 2**  **HR (95% CI)** |  | **Model 1**  **HR (95% CI)** | **Model 2**  **HR (95% CI)** |  | **Model 1**  **HR (95% CI)** | **Model 2**  **HR (95% CI)** |
| **TC** | < 20 years |  | 0.81 (0.63–1.04) | 0.80 (0.63–1.03) |  | *1.10 (1.004–1.21)** | *1.08 (0.98–1.18)* |  | *1.26 (1.18–1.35)** | *1.26 (1.17–1.35)** |
|  | ≥ 20 years |  | 0.91 (0.78–1.05) | *0.88 (0.76–1.03)* |  | 1.00 (0.90–1.10) | 0.98 (0.88–1.08) |  | 1.18 (1.05–1.32)* | 1.16 (1.03–1.30)* |
| **LDL-C** | < 20 years |  | 0.80 (0.63–1.02) | 0.80 (0.63–1.03) |  | *1.07 (0.98–1.17)* | *1.06 (0.96–1.16)* |  | *1.28 (1.19–1.37)** | *1.27 (1.19–1.36)** |
|  | ≥ 20 years |  | 0.87 (0.75–1.01) | 0.86 (0.74–1.003) |  | 0.98 (0.89–1.09) | 0.97 (0.88–1.08) |  | 1.17 (1.04–1.32)* | 1.16 (1.04–1.31)* |
| **HDL-C** | < 20 years |  | 0.96 (0.73–1.25) | 0.96 (0.73–1.28) |  | 0.83 (0.74–0.92)* | 0.86 (0.77–0.96)* |  | 0.73 (0.67–0.80)* | 0.75 (0.68–0.82)* |
|  | ≥ 20 years |  | 1.00 (0.85–1.18) | 1.00 (0.84–1.18) |  | 0.92 (0.82–1.03) | 0.93 (0.83–1.04) |  | 0.77 (0.67–0.89)* | 0.76 (0.66–0.88)* |
| **Non-HDL-C** | < 20 years |  | 0.82 (0.64–1.06) | 0.81 (0.63–1.05) |  | *1.16 (1.05–1.27)** | *1.12 (1.02–1.23)** |  | *1.36 (1.27–1.46)** | *1.34 (1.25–1.44)** |
|  | ≥ 20 years |  | 0.91 (0.78–1.06) | 0.89 (0.76–1.03) |  | 1.02 (0.93–1.13) | 1.00 (0.90–1.11) |  | 1.25 (1.12–1.41)* | 1.24 (1.10–1.39)* |
| **TC / HDL-C** | < 20 years |  | 0.89 (0.69–1.14) | 0.87 (0.67–1.13) |  | 1.27 (1.17–1.38)* | 1.23 (1.13–1.34)* |  | *1.42 (1.34–1.52)** | *1.40 (1.31–1.49)** |
|  | ≥ 20 years |  | 0.96 (0.83–1.12) | 0.95 (0.82–1.11) |  | 1.08 (0.98–1.19) | 1.06 (0.96–1.17) |  | 1.30 (1.16–1.45)* | 1.30 (1.16–1.46)* |
| **LDL-C / HDL-C** | < 20 years |  | 0.86 (0.67–1.11) | 0.85 (0.66–1.10) |  | 1.24 (1.14–1.36)* | 1.21 (1.11–1.32)* |  | *1.42 (1.33–1.51)** | *1.40 (1.31–1.49)** |
|  | ≥ 20 years |  | 0.93 (0.80–1.08) | 0.92 (0.79–1.07) |  | 1.06 (0.96–1.17) | 1.05 (0.94–1.16) |  | 1.28 (1.14–1.43)* | 1.29 (1.15–1.45)* |
| **ApoA1** | < 20 years |  | 0.90 (0.70–1.17) | 0.90 (0.68–1.19) |  | *0.90 (0.81–0.99)** | 0.92 (0.83–1.02) |  | 0.81 (0.75–0.88)* | 0.83 (0.76–0.90)* |
|  | ≥ 20 years |  | 1.01 (0.86–1.18) | 0.99 (0.84–1.16) |  | 0.93 (0.84–1.04) | 0.92 (0.83–1.03) |  | 0.89 (0.78–1.02) | 0.87 (0.76–1.001) |
| **ApoB** | < 20 years |  | 0.86 (0.68–1.09) | 0.85 (0.67–1.08) |  | *1.20 (1.09–1.31)** | *1.15 (1.05–1.26)** |  | *1.36 (1.27–1.45)** | *1.34 (1.25–1.43)** |
|  | ≥ 20 years |  | *0.97 (0.83–1.12)* | *0.94 (0.81–1.10)* |  | 1.03 (0.93–1.13) | 1.00 (0.90–1.10) |  | 1.25 (1.11–1.40)* | 1.23 (1.10–1.38)* |
| **ApoB / ApoA1** | < 20 years |  | 0.92 (0.72–1.17) | 0.90 (0.70–1.16) |  | 1.24 (1.14–1.34)* | 1.20 (1.10–1.31)* |  | *1.36 (1.28–1.43)** | *1.34 (1.27–1.42)** |
|  | ≥ 20 years |  | 0.97 (0.84–1.13) | 0.97 (0.83–1.13) |  | 1.10 (0.999–1.22) | 1.08 (0.98–1.20) |  | 1.27 (1.14–1.43)* | 1.27 (1.13–1.43)* |
| **Lp(a)** | < 20 years |  | 1.07 (0.85–1.35) | 1.06 (0.84–1.33) |  | 1.06 (0.97–1.16) | 1.07 (0.97–1.17) |  | 1.18 (1.10–1.26)* | 1.19 (1.11–1.27)* |
|  | ≥ 20 years |  | 0.99 (0.86–1.14) | 0.99 (0.86–1.14) |  | 1.03 (0.94–1.13) | 1.04 (0.94–1.14) |  | *1.07 (0.95–1.20)* | *1.06 (0.95–1.19)* |
| **Triglycerides** | < 20 years |  | 1.06 (0.84–1.34) | 1.03 (0.82–1.31) |  | 1.32 (1.21–1.44)* | 1.25 (1.14–1.37)* |  | 1.32 (1.23–1.42)* | 1.29 (1.20–1.39)* |
|  | ≥ 20 years |  | 1.06 (0.92–1.22) | 1.03 (0.89–1.19) |  | 1.12 (1.02–1.23)* | 1.07 (0.97–1.18) |  | 1.33 (1.19–1.49)* | 1.31 (1.16–1.47)* |
| **AIP** | < 20 years |  | 1.06 (0.84–1.34) | 1.04 (0.82–1.33) |  | 1.33 (1.21–1.46)* | 1.27 (1.15–1.39)* |  | *1.37 (1.28–1.47)** | 1.33 (1.24–1.44)* |
|  | ≥ 20 years |  | 1.05 (0.91–1.21) | 1.03 (0.89–1.19) |  | 1.12 (1.02–1.24)* | 1.08 (0.98–1.20) |  | 1.34 (1.20–1.51)* | 1.33 (1.18–1.50)* |

^a^For follow-up < 20 years, dementia N cases/ N Total=77/5306, 504/5306 for mortality, 824/5306 for CHD. In the follow-up ≥ 20 years the corresponding numbers for dementia were 205/4758, for mortality 448/4802, and for CHD 307/4088.

^b^Results in italics are from analyses where the proportionality hazards assumption did not hold.

Model 1: adjusted for age (as time scale), ethnicity, marital status, education, socioeconomic position, and use of lipids-lowering drugs, allowing the baseline hazard to differ by birth-cohort (5-years groups).

Model 2: Model 1 + health-related behaviours (smoking, alcohol consumption, physical activity, diet, and obesity).

Abbreviations: TC: Total Cholesterol, LDL-C: Low-Density Lipoprotein cholesterol, HDL-C: High-Density Lipoprotein cholesterol; ApoA1: Apolipoprotein A1; ApoB: Apolipoprotein B; Lp(a): Lipoprotein (a); AIP: Atherogenic Index of Plasma.

**p<0.05* (adjustment of the p-values after correction for multiple testing with False Discovery Rate analyses did not change the conclusions)

**eTable 8.** **Association of lipids (1991-1993) with dementia, mortality, and coronary heart disease over the follow-up (until March 2019) stratified by the length of follow-up in women.^a,b^**

|  |  |  | **Dementia** | |  | **Mortality** | |  | **Coronary Heart Disease** | |
| --- | --- | --- | --- | --- | --- | --- | --- | --- | --- | --- |
| **LIPIDS (per 1 SD increase)** | **Follow-up** |  | **Model 1**  **HR (95% CI)** | **Model 2**  **HR (95% CI)** |  | **Model 1**  **HR (95% CI)** | **Model 2**  **HR (95% CI)** |  | **Model 1**  **HR (95% CI)** | **Model 2**  **HR (95% CI)** |
| **TC** | < 20 years |  | 0.90 (0.69–1.18) | 0.90 (0.69–1.18) |  | 0.97 (0.85–1.11) | 0.94 (0.83–1.08) |  | 1.20 (1.07–1.34)* | 1.20 (1.07–1.35)* |
|  | ≥ 20 years |  | 1.28 (1.10–1.51)* | 1.29 (1.10–1.52)* |  | *0.98 (0.86–1.12)* | *0.98 (0.86–1.12)* |  | 1.25 (1.06–1.49)* | 1.27 (1.06–1.51)* |
| **LDL-C** | < 20 years |  | 0.90 (0.70–1.17) | 0.90 (0.69–1.17) |  | 1.01 (0.89–1.14) | 0.98 (0.86–1.12) |  | *1.22 (1.10–1.37)** | *1.21 (1.09–1.36)** |
|  | ≥ 20 years |  | 1.33 (1.14–1.54)* | 1.33 (1.14–1.55)* |  | 0.97 (0.86–1.11) | 0.96 (0.84–1.09) |  | 1.29 (1.10–1.52)* | 1.30 (1.10–1.53)* |
| **HDL-C** | < 20 years |  | 1.07 (0.84–1.37) | 1.11 (0.86–1.44) |  | 0.78 (0.68–0.89)* | 0.82 (0.71–0.94)* |  | *0.77 (0.68–0.88)** | *0.83 (0.73–0.95)** |
|  | ≥ 20 years |  | 0.79 (0.66–0.94)* | 0.84 (0.70–1.004) |  | *0.94 (0.83–1.07)* | *1.02 (0.90–1.17)* |  | 0.78 (0.65–0.93)* | 0.82 (0.68–0.99)* |
| **Non-HDL-C** | < 20 years |  | 0.88 (0.68–1.15) | 0.87 (0.66–1.14) |  | 1.06 (0.93–1.20) | 1.01 (0.89–1.15) |  | *1.28 (1.15–1.43)** | *1.26 (1.12–1.41)** |
|  | ≥ 20 years |  | 1.36 (1.16–1.58)* | 1.34 (1.15–1.57)* |  | 1.00 (0.88–1.14) | 0.97 (0.85–1.11) |  | 1.34 (1.13–1.58)* | 1.33 (1.12–1.57)* |
| **TC / HDL-C** | < 20 years |  | 0.81 (0.58–1.14) | 0.77 (0.54–1.09) |  | 1.14 (1.02–1.28)* | 1.08 (0.94–1.23) |  | 1.23 (1.14–1.34)* | 1.20 (1.09–1.31)* |
|  | ≥ 20 years |  | 1.29 (1.13–1.48)* | 1.25 (1.08–1.44)* |  | 1.05 (0.91–1.22) | 0.96 (0.82–1.13) |  | 1.21 (1.08–1.36)* | 1.16 (1.03–1.32)* |
| **LDL-C / HDL-C** | < 20 years |  | 0.82 (0.59–1.13) | 0.78 (0.56–1.10) |  | 1.12 (0.998–1.25) | 1.06 (0.93–1.20) |  | 1.21 (1.12–1.30)* | 1.18 (1.08–1.28)* |
|  | ≥ 20 years |  | 1.28 (1.12–1.46)* | 1.25 (1.09–1.43)* |  | 1.02 (0.88–1.18) | 0.94 (0.81–1.10) |  | 1.19 (1.07–1.33)* | 1.15 (1.03–1.29)* |
| **ApoA1** | < 20 years |  | 1.08 (0.83–1.41) | 1.11 (0.84–1.46) |  | 0.82 (0.72–0.94)* | 0.85 (0.74–0.97)* |  | 0.84 (0.74–0.95)* | 0.89 (0.79–1.01) |
|  | ≥ 20 years |  | 0.82 (0.68–0.98)* | 0.86 (0.71–1.04) |  | *0.99 (0.87–1.13)* | *1.06 (0.92–1.21)* |  | 0.80 (0.66–0.96)* | 0.84 (0.70–1.02) |
| **ApoB** | < 20 years |  | 0.89 (0.68–1.17) | 0.87 (0.66–1.16) |  | 1.05 (0.92–1.20) | 1.00 (0.87–1.14) |  | *1.28 (1.14–1.43)** | *1.25 (1.11–1.40)** |
|  | ≥ 20 years |  | 1.40 (1.19–1.64)* | 1.37 (1.16–1.61)* |  | 1.02 (0.89–1.16) | 0.98 (0.85–1.12) |  | 1.40 (1.18–1.66)* | 1.39 (1.17–1.66)* |
| **ApoB / ApoA1** | < 20 years |  | 0.84 (0.61–1.16) | 0.81 (0.58–1.13) |  | 1.12 (0.99–1.27) | 1.06 (0.92–1.21) |  | 1.25 (1.14–1.36)* | 1.21 (1.10–1.33)* |
|  | ≥ 20 years |  | 1.38 (1.19–1.59)* | 1.34 (1.15–1.56)* |  | 1.03 (0.88–1.19) | 0.95 (0.81–1.11) |  | 1.29 (1.15–1.45)* | 1.25 (1.10–1.41)* |
| **Lp(a)** | < 20 years |  | 1.04 (0.79–1.36) | 1.04 (0.79–1.37) |  | 1.10 (0.96–1.26) | 1.09 (0.96–1.25) |  | 1.16 (1.02–1.31)* | 1.14 (1.002–1.29)* |
|  | ≥ 20 years |  | 1.08 (0.90–1.29) | 1.06 (0.89–1.27) |  | 1.06 (0.93–1.22) | 1.05 (0.92–1.20) |  | 1.12 (0.93–1.34) | 1.11 (0.92–1.34) |
| **Triglycerides** | < 20 years |  | 0.91 (0.68–1.23) | 0.86 (0.63–1.18) |  | 1.25 (1.09–1.44)* | 1.14 (0.98–1.32) |  | 1.38 (1.21–1.57)* | 1.31 (1.14–1.50)* |
|  | ≥ 20 years |  | 1.25 (1.04–1.51)* | 1.17 (0.96–1.42) |  | 1.11 (0.96–1.28) | 1.02 (0.87–1.19) |  | 1.36 (1.13–1.65)* | 1.28 (1.05–1.57)* |
| **AIP** | < 20 years |  | 0.90 (0.67–1.21) | 0.85 (0.62–1.16) |  | *1.31 (1.13–1.51)** | 1.19 (1.02–1.39)* |  | *1.42 (1.25–1.62)** | *1.34 (1.17–1.53)** |
|  | ≥ 20 years |  | 1.31 (1.09–1.58)* | 1.21 (0.999–1.48) |  | 1.11 (0.96–1.29) | 1.01 (0.86–1.18) |  | 1.39 (1.16–1.68)* | 1.31 (1.07–1.60)* |

^a^For follow-up < 20 years, dementia N cases/ N Total=54/2366, 222/2366 for mortality, 263/2366 for CHD. In the follow-up ≥ 20 years the corresponding numbers for dementia were 126/2109, for mortality 216/2144, and for CHD 119/1891.

^b^Results in italics are from analyses where the proportionality hazards assumption did not hold.

Model 1: adjusted for age (as time scale), ethnicity, marital status, education, socioeconomic position, and use of lipids-lowering drugs, allowing the baseline hazard to differ by birth-cohort (5-years groups).

Model 2: Model 1 + health-related behaviours (smoking, alcohol consumption, physical activity, diet, and obesity).

Abbreviations: TC: Total Cholesterol, LDL-C: Low-Density Lipoprotein cholesterol, HDL-C: High-Density Lipoprotein cholesterol; ApoA1: Apolipoprotein A1; ApoB: Apolipoprotein B; Lp(a): Lipoprotein (a); AIP: Atherogenic Index of Plasma.

* *p<0.05* (adjustment of the p-values after correction for multiple testing with False Discovery Rate analyses did not change the conclusions)

**eTable 9. Association of lipids (1991-1993) with dementia, mortality, and coronary heart disease over the follow-up (until March 2019) stratified by the length of follow-up in men and adjusting for cardiovascular risk factors.^a,b^**

|  |  |  | **Dementia** |  | **Mortality** |  | **Coronary Heart Disease** |
| --- | --- | --- | --- | --- | --- | --- | --- |
| **LIPIDS (per 1 SD increase)** | **Follow-up** |  | **HR (95% CI)** |  | **HR (95% CI)** |  | **HR (95% CI)** |
| **TC** | < 20 years |  | 0.81 (0.63–1.03) |  | *1.07 (0.98–1.17)* |  | *1.25 (1.17–1.34)** |
|  | ≥ 20 years |  | *0.88 (0.76–1.03)* |  | 0.98 (0.88–1.08) |  | 1.15 (1.03–1.30)* |
| **LDL-C** | < 20 years |  | 0.80 (0.63–1.03) |  | *1.05 (0.96–1.15)* |  | *1.26 (1.18–1.36)** |
|  | ≥ 20 years |  | 0.86 (0.74–1.003) |  | 0.97 (0.88–1.08) |  | 1.16 (1.03–1.30)* |
| **HDL-C** | < 20 years |  | 0.96 (0.73–1.28) |  | 0.87 (0.78–0.97)* |  | 0.76 (0.70–0.84)* |
|  | ≥ 20 years |  | 1.01 (0.86–1.20) |  | 0.94 (0.84–1.05) |  | 0.77 (0.67–0.90)* |
| **Non-HDL-C** | < 20 years |  | 0.82 (0.64–1.05) |  | *1.11 (1.01–1.22)** |  | *1.33 (1.24–1.43)** |
|  | ≥ 20 years |  | 0.88 (0.75–1.03) |  | 0.99 (0.90–1.10) |  | 1.23 (1.09–1.38)* |
| **TC / HDL-C** | < 20 years |  | 0.87 (0.67–1.12) |  | 1.21 (1.11–1.32)* |  | *1.38 (1.29–1.47)** |
|  | ≥ 20 years |  | 0.93 (0.80–1.09) |  | 1.05 (0.95–1.16) |  | 1.29 (1.15–1.44)* |
| **LDL-C / HDL-C** | < 20 years |  | 0.85 (0.66–1.10) |  | 1.20 (1.09–1.31)* |  | *1.38 (1.29–1.47)** |
|  | ≥ 20 years |  | 0.90 (0.77–1.06) |  | 1.04 (0.94–1.15) |  | 1.27 (1.13–1.43)* |
| **ApoA1** | < 20 years |  | 0.90 (0.69–1.19) |  | 0.92 (0.83–1.02) |  | 0.84 (0.77–0.91)* |
|  | ≥ 20 years |  | 1.00 (0.85–1.17) |  | 0.93 (0.83–1.04) |  | 0.88 (0.76–1.01) |
| **ApoB** | < 20 years |  | 0.85 (0.67–1.08) |  | *1.14 (1.04–1.25)** |  | *1.32 (1.23–1.41)** |
|  | ≥ 20 years |  | 0.94 (0.81–1.09) |  | 0.99 (0.90–1.10) |  | 1.22 (1.09–1.37)* |
| **ApoB / ApoA1** | < 20 years |  | 0.90 (0.70–1.16) |  | 1.19 (1.09–1.29)* |  | *1.33 (1.25–1.41)** |
|  | ≥ 20 years |  | 0.95 (0.82–1.11) |  | 1.07 (0.97–1.19) |  | 1.26 (1.12–1.42)* |
| **Lp(a)** | < 20 years |  | 1.05 (0.83–1.32) |  | 1.07 (0.98–1.17) |  | 1.19 (1.11–1.28)* |
|  | ≥ 20 years |  | 0.99 (0.86–1.14) |  | 1.04 (0.94–1.14) |  | *1.07 (0.95–1.20)* |
| **Triglycerides** | < 20 years |  | 1.04 (0.82–1.32) |  | 1.23 (1.12–1.35)* |  | 1.27 (1.18–1.36)* |
|  | ≥ 20 years |  | 1.00 (0.87–1.16) |  | 1.06 (0.96–1.17) |  | 1.29 (1.15–1.45)* |
| **AIP** | < 20 years |  | 1.04 (0.81–1.33) |  | 1.24 (1.13–1.37)* |  | 1.31 (1.22–1.41)* |
|  | ≥ 20 years |  | 1.00 (0.87–1.17) |  | 1.32 (1.17–1.48)* |  | 1.32 (1.17–1.48)* |

^a^For follow-up < 20 years, dementia N cases/ N Total=77/5306, 504/5306 for mortality, 824/5306 for CHD. In the follow-up ≥ 20 years the corresponding numbers for dementia were 205/4758, for mortality 448/4802, and for CHD 307/4088.

^b^Results in italics are from analyses where the proportionality hazards assumption did not hold.

Model adjusted for age (as time scale), ethnicity, marital status, education, socioeconomic position, and use of lipids-lowering drugs, health-related behaviours (smoking, alcohol consumption, physical activity, diet, and obesity), hypertension and diabetes, allowing the baseline hazard to differ by birth-cohort (5-years groups).

Abbreviations: TC: Total Cholesterol, LDL-C: Low-Density Lipoprotein cholesterol, HDL-C: High-Density Lipoprotein cholesterol; ApoA1: Apolipoprotein A1; ApoB: Apolipoprotein B; Lp(a): Lipoprotein (a); AIP: Atherogenic Index of Plasma.

**p<0.05*

**eTable 10. Association of lipids (1991-1993) with dementia, mortality, and coronary heart disease over the follow-up (until March 2019) stratified by the length of follow-up in women and adjusting for cardiovascular risk factors.^a,b^**

|  |  |  | **Dementia** |  | **Mortality** |  | **Coronary Heart Disease** |
| --- | --- | --- | --- | --- | --- | --- | --- |
| **LIPIDS (per 1 SD increase)** | **Follow-up** |  | **HR (95% CI)** |  | **HR (95% CI)** |  | **HR (95% CI)** |
| **TC** | < 20 years |  | 0.89 (0.68–1.18) |  | 0.94 (0.82–1.08) |  | 1.20 (1.07–1.34)* |
|  | ≥ 20 years |  | 1.29 (1.10–1.52)* |  | *0.97 (0.85–1.11)* |  | 1.26 (1.05–1.50)* |
| **LDL-C** | < 20 years |  | 0.90 (0.69–1.17) |  | 0.98 (0.86–1.12) |  | *1.21 (1.08–1.35)** |
|  | ≥ 20 years |  | 1.32 (1.14–1.54)* |  | 0.96 (0.84–1.09) |  | 1.29 (1.09–1.53)* |
| **HDL-C** | < 20 years |  | 1.11 (0.86–1.43) |  | 0.82 (0.71–0.94)* |  | *0.83 (0.73–0.95)** |
|  | ≥ 20 years |  | 0.84 (0.70–1.003) |  | *1.02 (0.90–1.17)* |  | 0.82 (0.68–0.996)* |
| **Non-HDL-C** | < 20 years |  | 0.86 (0.66–1.14) |  | 1.01 (0.88–1.15) |  | *1.25 (1.12–1.40)** |
|  | ≥ 20 years |  | 1.34 (1.14–1.57)* |  | 0.97 (0.85–1.11) |  | 1.32 (1.11–1.56)* |
| **TC / HDL-C** | < 20 years |  | 0.76 (0.53–1.08) |  | 1.07 (0.94–1.22) |  | 1.18 (1.08–1.30)* |
|  | ≥ 20 years |  | 1.25 (1.08–1.45)* |  | 0.95 (0.81–1.11) |  | 1.15 (1.01–1.30)* |
| **LDL-C / HDL-C** | < 20 years |  | 0.78 (0.56–1.09) |  | 1.06 (0.93–1.20) |  | 1.17 (1.07–1.27)* |
|  | ≥ 20 years |  | 1.25 (1.09–1.44)* |  | 0.94 (0.80–1.09) |  | 1.14 (1.01–1.28)* |
| **ApoA1** | < 20 years |  | 1.10 (0.84–1.44) |  | 0.85 (0.74–0.97)* |  | 0.89 (0.78–1.004) |
|  | ≥ 20 years |  | 0.86 (0.71–1.04) |  | *1.05 (0.92–1.21)* |  | 0.84 (0.70–1.01) |
| **ApoB** | < 20 years |  | 0.87 (0.66–1.15) |  | 0.99 (0.87–1.14) |  | *1.24 (1.11–1.39)** |
|  | ≥ 20 years |  | 1.36 (1.15–1.60)* |  | 0.97 (0.85–1.11) |  | 1.38 (1.16–1.65)* |
| **ApoB / ApoA1** | < 20 years |  | 0.81 (0.58–1.12) |  | 1.05 (0.91–1.20) |  | 1.20 (1.09–1.32)* |
|  | ≥ 20 years |  | 1.34 (1.15–1.56)* |  | 0.94 (0.81–1.10) |  | 1.23 (1.09–1.39)* |
| **Lp(a)** | < 20 years |  | 1.04 (0.79–1.37) |  | 1.09 (0.96–1.25) |  | 1.14 (1.01–1.29)* |
|  | ≥ 20 years |  | 1.06 (0.88–1.27) |  | 1.05 (0.92–1.21) |  | 1.12 (0.93–1.36) |
| **Triglycerides** | < 20 years |  | 0.84 (0.61–1.14) |  | 1.13 (0.97–1.31) |  | 1.30 (1.14–1.49)* |
|  | ≥ 20 years |  | 1.16 (0.96–1.42) |  | 1.00 (0.86–1.17) |  | 1.26 (1.03–1.55)* |
| **AIP** | < 20 years |  | 0.83 (0.61–1.13) |  | 1.18 (1.02–1.38)* |  | *1.33 (1.16–1.52)** |
|  | ≥ 20 years |  | 1.21 (0.99–1.47) |  | 0.99 (0.85–1.16) |  | 1.29 (1.05–1.58)* |

^a^For follow-up < 20 years, dementia N cases/ N Total=54/2366, 222/2366 for mortality, 263/2366 for CHD. In the follow-up ≥ 20 years the corresponding numbers for dementia were 126/2109, for mortality 216/2144, and for CHD 119/1891.

^b^Results in italics are from analyses where the proportionality hazards assumption did not hold.

Model adjusted for age (as time scale), ethnicity, marital status, education, socioeconomic position, and use of lipids-lowering drugs, health-related behaviours (smoking, alcohol consumption, physical activity, diet, and obesity), hypertension and diabetes, allowing the baseline hazard to differ by birth-cohort (5-years groups).

Abbreviations: TC: Total Cholesterol, LDL-C: Low-Density Lipoprotein cholesterol, HDL-C: High-Density Lipoprotein cholesterol; ApoA1: Apolipoprotein A1; ApoB: Apolipoprotein B; Lp(a): Lipoprotein (a); AIP: Atherogenic Index of Plasma.

* *p<0.05*

**eTable 11. Association of lipids (1991-1993) with dementia, mortality, and coronary heart disease over the follow-up (until March 2019) in men without APOE ε4 allele(s).^a,b^**

|  |  | **Dementia** | |  | **Mortality** | |  | **Coronary Heart Disease** | | |
| --- | --- | --- | --- | --- | --- | --- | --- | --- | --- | --- |
| **LIPIDS (per 1 SD increase)** | **Length of follow-up** | **Model 1**  **HR (95% CI)** | **Model 2**  **HR (95% CI)** |  | **Model 1**  **HR (95% CI)** | **Model 2**  **HR (95% CI)** |  | **Model 1**  **HR (95% CI)** | **Model 2**  **HR (95% CI)** |  |
| **TC** | < 20 years | 0.84 (0.62–1.15) | 0.82 (0.60–1.11) |  | *1.13 (1.03–1.25)** | *1.10 (1.001–1.22)** |  | *1.25 (1.15–1.35)** | *1.24 (1.15–1.34)** |  |
|  | ≥ 20 years | 0.84 (0.70–1.02) | 0.82 (0.68–0.99)* |  | 0.98 (0.88–1.10) | 0.96 (0.85–1.08) |  | 1.18 (1.04–1.35)* | 1.17 (1.03–1.34)* |  |
| **LDL-C** | < 20 years | 0.81 (0.60–1.11) | 0.81 (0.59–1.10) |  | *1.10 (0.998–1.21)* | *1.08 (0.98–1.19)* |  | *1.26 (1.17–1.37)** | *1.26 (1.16–1.36)** |  |
|  | ≥ 20 years | 0.82 (0.68–0.99)* | 0.81 (0.67–0.98)* |  | 0.96 (0.86–1.08) | 0.95 (0.85–1.07) |  | 1.17 (1.03–1.34)* | 1.17 (1.03–1.34)* |  |
| **HDL-C** | < 20 years | 0.98 (0.69–1.37) | 0.94 (0.65–1.34) |  | 0.84 (0.74–0.94)* | 0.86 (0.76–0.97)* |  | 0.74 (0.67–0.82)* | 0.76 (0.69–0.84)* |  |
|  | ≥ 20 years | 1.00 (0.82–1.21) | 1.01 (0.82–1.23) |  | 0.89 (0.78–1.01) | 0.90 (0.79–1.03) |  | 0.77 (0.66–0.91)* | 0.77 (0.65–0.92)* |  |
| **Non-HDL-C** | < 20 years | 0.85 (0.62–1.16) | 0.83 (0.61–1.13) |  | *1.18 (1.07–1.31)** | *1.14 (1.04–1.26)** |  | *1.34 (1.24–1.44)** | *1.32 (1.22–1.42)** |  |
|  | ≥ 20 years | 0.85 (0.70–1.02) | 0.83 (0.68–0.998)* |  | 1.01 (0.90–1.14) | 0.99 (0.88–1.11) |  | 1.26 (1.10–1.43)* | 1.25 (1.09–1.42)* |  |
| **TC / HDL-C** | < 20 years | 0.88 (0.64–1.22) | 0.88 (0.63–1.23) |  | 1.27 (1.16–1.39)* | 1.234 (1.13–1.36)* |  | *1.41 (1.31–1.51)** | *1.39 (1.29–1.49)** |  |
|  | ≥ 20 years | 0.93 (0.78–1.12) | 0.91 (0.75–1.10) |  | 1.10 (0.99–1.23) | 1.08 (0.96–1.21) |  | 1.29 (1.14–1.47)* | 1.29 (1.13–1.48)* |  |
| **LDL-C / HDL-C** | < 20 years | 0.84 (0.61–1.16) | 0.85 (0.60–1.19) |  | *1.25 (1.14–1.37)** | 1.22 (1.11–1.34)* |  | *1.40 (1.31–1.51)** | 1.38 (1.28–1.49)* |  |
|  | ≥ 20 years | 0.90 (0.75–1.09) | 0.88 (0.73–1.07) |  | 1.08 (0.96–1.20) | 1.06 (0.94–1.19) |  | 1.28 (1.12–1.45)* | 1.28 (1.12–1.46)* |  |
| **ApoA1** | < 20 years | 0.10 (0.75–1.41) | 0.97 (0.69–1.37) |  | 0.90 (0.81–1.01) | 0.91 (0.82–1.02) |  | 0.83 (0.75–0.90)* | 0.84 (0.77–0.92)* |  |
|  | ≥ 20 years | 0.97 (0.80–1.18) | 0.96 (0.79–1.18) |  | 0.93 (0.82–1.04) | 0.92 (0.81–1.05) |  | 0.91 (0.78–1.05) | 0.90 (0.77–1.05) |  |
| **ApoB** | < 20 years | 0.84 (0.62–1.14) | 0.81 (0.60–1.10) |  | *1.23 (1.12–1.35)** | *1.18 (1.07–1.30)** |  | *1.34 (1.24–1.44)** | *1.31 (1.22–1.42)** |  |
|  | ≥ 20 years | 0.92 (0.76–1.10) | 0.89 (0.74–1.07) |  | 1.01 (0.91–1.13) | 0.98 (0.88–1.10) |  | 1.27 (1.11–1.44)* | 1.25 (1.10–1.42)* |  |
| **ApoB / ApoA1** | < 20 years | 0.81 (0.59–1.12) | 0.80 (0.57–1.12) |  | 1.28 (1.16–1.40)* | 1.23 (1.12–1.36)* |  | *1.39 (1.29–1.49)** | *1.36 (1.26–1.47)** |  |
|  | ≥ 20 years | 0.97 (0.81–1.16) | 0.95 (0.79–1.15) |  | 1.07 (0.96–1.20) | 1.05 (0.93–1.18) |  | 1.29 (1.13–1.47)* | 1.28 (1.12–1.47)* |  |
| **Lp(a)** | < 20 years | 1.15 (0.86–1.54) | 1.11 (0.83–1.49) |  | 1.08 (0.98–1.19) | 1.08 (0.98–1.19) |  | 1.19 (1.10–1.28)* | 1.20 (1.11–1.29)* |  |
|  | ≥ 20 years | 1.04 (0.87–1.24) | 1.04 (0.88–1.24) |  | 1.001 (0.91–1.12) | 1.01 (0.91–1.13) |  | 1.08 (0.95–1.23) | 1.08 (0.95–1.23) |  |
| **Triglycerides** | < 20 years | 1.07 (0.80–1.43) | 1.01 (0.75–1.37) |  | 1.33 (1.21–1.47)* | 1.26 (1.14–1.39)* |  | *1.30 (1.20–1.40)** | *1.26 (1.16–1.37)** |  |
|  | ≥ 20 years | 1.03 (0.86–1.22) | 0.99 (0.82–1.18) |  | 1.16 (1.04–1.29)* | 1.11 (0.99–1.24) |  | 1.35 (1.19–1.54)* | 1.33 (1.16–1.52)* |  |
| **AIP** | < 20 years | 1.07 (0.79–1.44) | 1.04 (0.76–1.41) |  | 1.34 (1.21–1.47)* | 1.27 (1.15–1.41)* |  | *1.35 (1.24–1.46)** | *1.31 (1.21–1.42)** |  |
|  | ≥ 20 years | 1.03 (0.86–1.23) | 0.99 (0.83–1.20) |  | 1.17 (1.04–1.30)* | 1.13 (1.004–1.27)* |  | 1.36 (1.19–1.56)* | 1.35 (1.17–1.54)* |  |

^a^For follow-up < 20 years, dementia N cases/N Total=49/4217, 439/4217 for mortality, 676/4217 for CHD. In the follow-up ≥ 20 years the corresponding numbers for dementia were 137/3751, for mortality 351/3778, and for CHD 241/3207.

^b^Results in italics are from analyses where the proportionality hazards assumption did not hold.

Model 1: adjusted for age (as time scale), ethnicity, marital status, education, socioeconomic position, and use of lipids-lowering drugs, allowing the baseline hazard to differ by birth-cohort (5-years groups).

Model 2: Model 1 + health-related behaviours (smoking, alcohol consumption, physical activity, diet, and obesity).

Abbreviations: TC: Total Cholesterol, LDL-C: Low-Density Lipoprotein cholesterol, HDL-C: High-Density Lipoprotein cholesterol; ApoA1: Apolipoprotein A1; ApoB: Apolipoprotein B; Lp(a): Lipoprotein (a); AIP: Atherogenic Index of Plasma.

**p<0.05*

**eTable 12. Association of lipids (1991-1993) with dementia, mortality, and coronary heart disease over the follow-up (until March 2019) in women without APOE ε4 allele(s).^a^**

|  |  | **Dementia** | |  | **Mortality** | |  | **Coronary Heart Disease** | |
| --- | --- | --- | --- | --- | --- | --- | --- | --- | --- |
| **LIPIDS (per 1 SD increase)** | **Length of follow-up** | **Model 1**  **HR (95% CI)** | **Model 2**  **HR (95% CI)** |  | **Model 1**  **HR (95% CI)** | **Model 2**  **HR (95% CI)** |  | **Model 1**  **HR (95% CI)** | **Model 2**  **HR (95% CI)** |
| **TC** | < 20 years | 0.92 (0.67–1.27) | 0.91 (0.66–1.27) |  | 1.01 (0.88–1.16) | 0.99 (0.86–1.14) |  | 1.20 (1.06–1.36)* | 1.20 (1.06–1.36)* |
|  | ≥ 20 years | 1.33 (1.11–1.59)* | 1.36 (1.13–1.64)* |  | *1.00 (0.87–1.16)* | *1.00 (0.86–1.15)* |  | 1.25 (1.03–1.51)* | 1.26 (1.03–1.53)* |
| **LDL-C** | < 20 years | 0.89 (0.65–1.22) | 0.88 (0.64–1.22) |  | 1.04 (0.91–1.19) | 1.02 (0.89–1.16) |  | *1.24 (1.11–1.40)** | 1.23 (1.09–1.39)* |
|  | ≥ 20 years | 1.36 (1.15–1.62)* | 1.39 (1.17–1.66)* |  | 0.99 (0.86–1.14) | 0.98 (0.85–1.13) |  | 1.30 (1.08–1.56)* | 1.30 (1.08–1.58)* |
| **HDL-C** | < 20 years | 0.88 (0.64–1.21) | 1.21 (0.89–1.65) |  | 0.77 (0.66–0.88)* | 0.82 (0.70–0.95)* |  | 0.73 (0.63–0.84)* | 0.78 (0.67–0.90)* |
|  | ≥ 20 years | 0.77 (0.63–0.95)* | 0.82 (0.66–1.02) |  | *0.95 (0.83–1.10)* | *1.04 (0.89–1.20)* |  | *0.74 (0.60–0.92)** | *0.79 (0.63–0.98)** |
| **Non-HDL-C** | < 20 years | 1.15 (0.85–1.54) | 0.86 (0.62–1.19) |  | 1.10 (0.96–1.26) | 1.05 (0.92–1.21) |  | *1.30 (1.15–1.47)** | *1.27 (1.13–1.44)** |
|  | ≥ 20 years | 1.41 (1.18–1.69)* | 1.42 (1.18–1.70)* |  | 1.02 (0.88–1.17) | 0.98 (0.85–1.14) |  | 1.35 (1.12–1.62)* | 1.33 (1.10–1.61)* |
| **TC / HDL-C** | < 20 years | 0.79 (0.52–1.20) | 0.72 (0.47–1.12) |  | 1.16 (1.04–1.29)* | 1.09 (0.96–1.23) |  | 1.26 (1.15–1.38)* | 1.23 (1.12–1.36)* |
|  | ≥ 20 years | 1.29 (1.11–1.50)* | 1.26 (1.07–1.48)* |  | 1.07 (0.91–1.25) | 0.98 (0.82–1.16) |  | 1.23 (1.08–1.40)* | 1.19 (1.03–1.38)* |
| **LDL-C / HDL-C** | < 20 years | 0.79 (0.53–1.18) | 0.74 (0.48–1.13) |  | 1.13 (1.02–1.25)* | 1.07 (0.95–1.21) |  | 1.24 (1.14–1.35)* | 1.21 (1.11–1.33)* |
|  | ≥ 20 years | 1.27 (1.10–1.48)* | 1.26 (1.08–1.47)* |  | 1.04 (0.90–1.22) | 0.97 (0.82–1.14) |  | 1.21 (1.07–1.37)* | 1.18 (1.04–1.35)* |
| **ApoA1** | < 20 years | 1.12 (0.81–1.53) | 1.15 (0.83–1.59) |  | 0.82 (0.71–0.94)* | 0.85 (0.74–0.99)* |  | 0.80 (0.70–0.92)* | 0.85 (0.74–0.97)* |
|  | ≥ 20 years | 0.82 (0.67–1.01) | 0.87 (0.70–1.08) |  | *0.98 (0.84–1.13)* | *1.04 (0.90–1.21)* |  | *0.77 (0.63–0.95)** | *0.81 (0.66–1.003)* |
| **ApoB** | < 20 years | 0.91 (0.66–1.27) | 0.88 (0.63–1.24) |  | 1.11 (0.97–1.27) | 1.05 (0.91–1.21) |  | *1.31 (1.16–1.47)** | *1.27 (1.13–1.44)** |
|  | ≥ 20 years | 1.49 (1.24–1.78)* | 1.49 (1.23–1.79)* |  | 1.05 (0.91–1.21) | 1.00 (0.87–1.17) |  | 1.43 (1.18–1.73)* | 1.41 (1.16–1.72)* |
| **ApoB / ApoA1** | < 20 years | 0.86 (0.58–1.26) | 0.81 (0.54–1.21) |  | 1.14 (1.02–1.28)* | 1.08 (0.94–1.23) |  | 1.28 (1.17–1.41)* | 1.25 (1.13–1.39)* |
|  | ≥ 20 years | 1.38 (1.17–1.62)* | 1.38 (1.16–1.63)* |  | 1.06 (0.91–1.24) | 0.99 (0.84–1.16) |  | 1.33 (1.16–1.51)* | 1.29 (1.12–1.49)* |
| **Lp(a)** | < 20 years | *1.13 (0.81–1.57)* | *1.14 (0.82–1.58)* |  | 1.15 (0.996–1.32) | 1.14 (0.99–1.31) |  | 1.19 (1.04–1.37)* | 1.18 (1.03–1.35)* |
|  | ≥ 20 years | 1.06 (0.86–1.30) | 1.05 (0.85–1.28) |  | 1.14 (0.98–1.32) | 1.12 (0.96–1.30) |  | 1.11 (0.90–1.37) | 1.11 (0.89–1.37) |
| **Triglycerides** | < 20 years | 0.94 (0.66–1.34) | 0.87 (0.60–1.26) |  | 1.31 (1.13–1.51)* | 1.19 (1.02–1.39)* |  | 1.38 (1.21–1.58)* | 1.32 (1.15–1.53)* |
|  | ≥ 20 years | 1.32 (1.07–1.64)* | 1.23 (0.98–1.53) |  | 1.08 (0.92–1.27) | 0.99 (0.84–1.17) |  | 1.36 (1.10–1.67)* | 1.26 (1.01–1.58)* |
| **AIP** | < 20 years | 0.90 (0.63–1.29) | 0.82 (0.56–1.20) |  | 1.36 (1.17–1.57)* | 1.23 (1.05–1.44)* |  | 1.45 (1.27–1.66)* | 1.38 (1.19–1.59)* |
|  | ≥ 20 years | 1.37 (1.11–1.69)* | 1.27 (1.01–1.58)* |  | 1.09 (0.93–1.28) | 0.98 (0.83–1.17) |  | 1.41 (1.14–1.73)* | 1.31 (1.05–1.64)* |

^a^For follow-up < 20 years, dementia N cases/N Total=38/1961, 200/1961 for mortality, 224/1961 for CHD. In the follow-up ≥ 20 years the corresponding numbers for dementia were 96/1740, for mortality 180/1761, and for CHD 96/1557.

^b^Results in italics are from analyses where the proportionality hazards assumption did not hold.

Model 1: adjusted for age (as time scale), ethnicity, marital status, education, socioeoconomic position, and use of lipids-lowering drugs, allowing the baseline hazard to differ by birth-cohort (5-years groups).

Model 2: Model 1 + health-related behaviours (smoking, alcohol consumption, physical activity, diet, and obesity).

Abbreviations: TC: Total Cholesterol, LDL-C: Low-Density Lipoprotein cholesterol, HDL-C: High-Density Lipoprotein cholesterol; ApoA1: Apolipoprotein A1; ApoB: Apolipoprotein B; Lp(a): Lipoprotein (a); AIP: Atherogenic Index of Plasma.

**p<0.05*

**eTable 13. Association of lipids (1991-1993) with dementia, mortality, and coronary heart disease over the follow-up (until March 2019) in men without APOE ε2 allele(s).^a,b^**

|  |  | **Dementia** | |  | **Mortality** | |  | **Coronary Heart Disease** | | |
| --- | --- | --- | --- | --- | --- | --- | --- | --- | --- | --- |
| **LIPIDS (per 1 SD increase)** | **Length of follow-up** | **Model 1**  **HR (95% CI)** | **Model 2**  **HR (95% CI)** |  | **Model 1**  **HR (95% CI)** | **Model 2**  **HR (95% CI)** |  | **Model 1**  **HR (95% CI)** | **Model 2**  **HR (95% CI)** |  |
| **TC** | < 20 years | 0.74 (0.57–0.95)* | 0.74 (0.57–0.95)* |  | *1.08 (0.99–1.19)* | *1.06 (0.97–1.17)* |  | *1.25 (1.16–1.35)** | *1.25 (1.16–1.35)** |  |
|  | ≥ 20 years | 0.89 (0.76–1.05) | 0.87 (0.74–1.02) |  | 0.99 (0.89–1.10) | 0.97 (0.87–1.08) |  | 1.19 (1.06–1.35)* | 1.18 (1.05–1.34)* |  |
| **LDL-C** | < 20 years | 0.72 (0.56–0.93)* | 0.73 (0.56–0.93)* |  | *1.04 (0.94–1.14)* | *1.02 (0.93–1.13)* |  | *1.27 (1.18–1.37)** | *1.26 (1.17–1.36)** |  |
|  | ≥ 20 years | 0.85 (0.72–0.99)* | 0.84 (0.72–0.99)* |  | 0.97 (0.87–1.08) | 0.96 (0.86–1.07) |  | 1.18 (1.05–1.34)* | 1.18 (1.04–1.33)* |  |
| **HDL-C** | < 20 years | 0.97 (0.74–1.27) | 0.98 (0.74–1.30) |  | 0.87 (0.78–0.97)* | 0.90 (0.80–1.01) |  | 0.72 (0.65–0.79)* | 0.74 (0.68–0.82)* |  |
|  | ≥ 20 years | 1.01 (0.85–1.19) | 0.99 (0.84–1.19) |  | 0.91 (0.81–1.02) | 0.92 (0.81–1.04) |  | 0.78 (0.67–0.90)* | 0.77 (0.66–0.90)* |  |
| **Non-HDL-C** | < 20 years | 0.75 (0.58–0.97)* | 0.75 (0.58–0.97)* |  | *1.13 (1.02–1.24)** | *1.09 (0.99–1.20)* |  | *1.35 (1.26–1.45)** | *1.33 (1.24–1.44)** |  |
|  | ≥ 20 years | 0.89 (0.76–1.05) | 0.87 (0.74–1.03) |  | 1.01 (0.91–1.13) | 0.99 (0.89–1.11) |  | 1.27 (1.12–1.44)* | 1.26 (1.11–1.42)* |  |
| **TC / HDL-C** | < 20 years | 0.84 (0.65–1.09) | 0.82 (0.63–1.08) |  | 1.23 (1.13–1.34)* | 1.19 (1.09–1.30)* |  | 1.43 (1.34–1.53)* | 1.40 (1.31–1.50)* |  |
|  | ≥ 20 years | 0.95 (0.81–1.11) | 0.95 (0.81–1.11) |  | 1.08 (0.97–1.20) | 1.06 (0.95–1.18) |  | 1.31 (1.17–1.47)* | 1.32 (1.17–1.49)* |  |
| **LDL-C / HDL-C** | < 20 years | 0.80 (0.62–1.04) | 0.79 (0.60–1.04) |  | 1.20 (1.10–1.31)* | 1.16 (1.06–1.28)* |  | *1.43 (1.33–1.53)** | *1.40 (1.30–1.50)** |  |
|  | ≥ 20 years | 0.91 (0.77–1.07) | 0.91 (0.77–1.07) |  | 1.06 (0.95–1.17) | 1.04 (0.93–1.16) |  | 1.29 (1.15–1.46)* | 1.30 (1.15–1.47)* |  |
| **ApoA1** | < 20 years | 0.91 (0.70–1.19) | 0.91 (0.68–1.20) |  | 0.94 (0.85–1.04) | 0.97 (0.87–1.07) |  | 0.79 (0.73–0.87)* | 0.82 (0.75–0.90)* |  |
|  | ≥ 20 years | 1.00 (0.85–1.18) | 0.97 (0.82–1.16) |  | 0.93 (0.83–1.04) | 0.92 (0.82–1.04) |  | 0.90 (0.78–1.04) | 0.89 (0.77–1.03) |  |
| **ApoB** | < 20 years | 0.77 (0.60–0.99)* | 0.77 (0.60–0.99)* |  | *1.15 (1.05–1.26)** | *1.11 (1.01–1.22)** |  | *1.36 (1.26–1.46)** | *1.34 (1.24–1.44)** |  |
|  | ≥ 20 years | 0.93 (0.80–1.09) | 0.91 (0.78–1.07) |  | 1.01 (0.91–1.12) | 0.98 (0.88–1.10) |  | 1.26 (1.12–1.42)* | 1.25 (1.10–1.41)* |  |
| **ApoB / ApoA1** | < 20 years | 0.84 (0.65–1.08) | 0.823 (0.64–1.08) |  | 1.20 (1.09–1.31)* | 1.15 (1.05–1.26)* |  | *1.44 (1.34–1.54)** | *1.40 (1.30–1.51)** |  |
|  | ≥ 20 years | 0.95 (0.81–1.11) | 0.95 (0.81–1.12) |  | 1.07 (0.96–1.19) | 1.05 (0.94–1.17) |  | 1.28 (1.13–1.45)* | 1.28 (1.13–1.45)* |  |
| **Lp(a)** | < 20 years | 1.08 (0.86–1.37) | 1.08 (0.85–1.37) |  | 1.05 (0.96–1.15) | 1.06 (0.96–1.16) |  | 1.18 (1.10–1.28)* | 1.20 (1.11–1.29)* |  |
|  | ≥ 20 years | 0.98 (0.84–1.14) | 0.98 (0.84–1.14) |  | 1.03 (0.94–1.14) | 1.04 (0.94–1.15) |  | *1.10 (0.98–1.24)* | *1.10 (0.97–1.24)* |  |
| **Triglycerides** | < 20 years | 1.06 (0.84–1.35) | 1.04 (0.82–1.33) |  | 1.33 (1.21–1.46)* | 1.26 (1.14–1.39)* |  | *1.32 (1.23–1.42)** | 1.29 (1.19–1.39)* |  |
|  | ≥ 20 years | 1.08 (0.93–1.26) | 1.05 (0.90–1.23) |  | 1.14 (1.03–1.27)* | 1.09 (0.99–1.22) |  | 1.35 (1.19–1.52)* | 1.33 (1.17–1.51)* |  |
| **AIP** | < 20 years | 1.06 (0.83–1.35) | 1.04 (0.81–1.34) |  | 1.32 (1.20–1.45)* | 1.25 (1.14–1.38)* |  | 1.38 (1.28–1.48)* | 1.33 (1.23–1.44)* |  |
|  | ≥ 20 years | 1.06 (0.91–1.24) | 1.05 (0.90–1.22) |  | 1.14 (1.03–1.27)* | 1.10 (0.99–1.23) |  | 1.35 (1.20–1.53)* | 1.35 (1.19–1.53)* |  |

^a^For follow-up < 20 years, dementia N cases/N Total=74/4709, 477/4709 for mortality, 736/4709 for CHD. In the follow-up ≥ 20 years the corresponding numbers for dementia were 185/4190, for mortality 394/4232, and for CHD 272/3601.

^b^Results in italics are from analyses where the proportionality hazards assumption did not hold.

Model 1: adjusted for age (as time scale), ethnicity, marital status, education, socioeconomic position, and use of lipids-lowering drugs, allowing the baseline hazard to differ by birth-cohort (5-years groups).

Model 2: Model 1 + health-related behaviours (smoking, alcohol consumption, physical activity, diet, and obesity).

Abbreviations: TC: Total Cholesterol, LDL-C: Low-Density Lipoprotein cholesterol, HDL-C: High-Density Lipoprotein cholesterol; ApoA1: Apolipoprotein A1; ApoB: Apolipoprotein B; Lp(a): Lipoprotein (a); AIP: Atherogenic Index of Plasma.

**p<0.05*

**eTable 14. Association of lipids (1991-1993) with dementia, mortality, and coronary heart disease over the follow-up (until March 2019) in women without APOE ε2 allele(s).^a^**

|  |  | **Dementia** | |  | **Mortality** | |  | **Coronary Heart Disease** | |
| --- | --- | --- | --- | --- | --- | --- | --- | --- | --- |
| **LIPIDS (per 1 SD increase)** | **Length of follow-up** | **Model 1**  **HR (95% CI)** | **Model 2**  **HR (95% CI)** |  | **Model 1**  **HR (95% CI)** | **Model 2**  **HR (95% CI)** |  | **Model 1**  **HR (95% CI)** | **Model 2**  **HR (95% CI)** |
| **TC** | < 20 years | 0.94 (0.71–1.24) | 0.93 (0.70–1.24) |  | 1.00 (0.87–1.15) | 0.98 (0.85–1.13) |  | 1.22 (1.09–1.37)* | 1.23 (1.09–1.38)* |
|  | ≥ 20 years | 1.29 (1.09–1.52)* | 1.30 (1.10–1.55)* |  | 1.00 (0.87–1.15) | *0.99 (0.86–1.14)* |  | 1.21 (1.01–1.44)* | 1.22 (1.02–1.46)* |
| **LDL-C** | < 20 years | 0.96 (0.73–1.25) | 0.96 (0.73–1.25) |  | 1.04 (0.91–1.19) | 1.02 (0.89–1.16) |  | 1.25 (1.11–1.40)* | 1.23 (1.10–1.38)* |
|  | ≥ 20 years | 1.33 (1.14–1.56)* | 1.33 (1.14–1.56)* |  | 0.98 (0.86–1.12) | 0.96 (0.84–1.10) |  | 1.23 (1.04–1.46)* | 1.24 (1.05–1.47)* |
| **HDL-C** | < 20 years | 1.03 (0.79–1.35) | 1.04 (0.79–1.37) |  | 0.76 (0.66–0.88)* | 0.80 (0.69–0.93)* |  | 0.76 (0.67–0.87)* | 0.82 (0.72–0.94)* |
|  | ≥ 20 years | 0.82 (0.68–0.98)* | 0.87 (0.72–1.05) |  | *0.96 (0.84–1.10)* | *1.05 (0.91–1.21)* |  | 0.78 (0.65–0.95)* | 0.83 (0.68–1.002) |
| **Non-HDL-C** | < 20 years | 0.93 (0.71–1.22) | 0.93 (0.70–1.22) |  | 1.10 (0.96–1.25) | 1.05 (0.92–1.21) |  | 1.31 (1.17–1.47)* | 1.29 (1.14–1.44)* |
|  | ≥ 20 years | 1.35 (1.15–1.59)* | 1.34 (1.13–1.58)* |  | 1.01 (0.89–1.16) | 0.98 (0.85–1.12) |  | 1.29 (1.09–1.52)* | 1.28 (1.07–1.52)* |
| **TC / HDL-C** | < 20 years | 0.84 (0.60–1.20) | 0.82 (0.57–1.18) |  | 1.16 (1.04–1.30)* | 1.10 (0.97–1.25) |  | 1.24 (1.14–1.34)* | 1.20 (1.10–1.32)* |
|  | ≥ 20 years | 1.26 (1.09–1.46)* | 1.22 (1.05–1.42)* |  | 1.05 (0.90–1.23) | 0.95 (0.80–1.13) |  | 1.20 (1.06–1.36)* | 1.16 (1.02–1.33)* |
| **LDL-C / HDL-C** | < 20 years | 0.86 (0.61–1.20) | 0.84 (0.60–1.19) |  | 1.13 (1.02–1.26)* | 1.08 (0.96–1.22) |  | 1.21 (1.12–1.31)* | 1.18 (1.08–1.28)* |
|  | ≥ 20 years | 1.26 (1.10–1.44)* | 1.22 (1.06–1.42)* |  | 1.03 (0.88–1.20) | 0.93 (0.79–1.10) |  | 1.18 (1.05–1.33)* | 1.15 (1.01–1.30)* |
| **ApoA1** | < 20 years | *1.02 (0.77–1.35)* | 1.02 (0.77–1.37) |  | 0.81 (0.70–0.93)* | 0.83 (0.72–0.96)* |  | 0.83 (0.73–0.94)* | 0.89 (0.78–1.02) |
|  | ≥ 20 years | 0.82 (0.68–0.99)* | 0.86 (0.71–1.04) |  | *1.00 (0.87–1.15)* | *1.08 (0.93–1.25)* |  | 0.79 (0.66–0.95)* | 0.83 (0.68–1.005) |
| **ApoB** | < 20 years | 0.93 (0.70–1.23) | 0.92 (0.69–1.23) |  | 1.10 (0.96–1.26) | 1.04 (0.90–1.20) |  | *1.30 (1.16–1.46)** | *1.27 (1.13–1.43)** |
|  | ≥ 20 years | 1.39 (1.17–1.64)* | 1.36 (1.15–1.61)* |  | 1.03 (0.89–1.18) | 0.98 (0.85–1.13) |  | 1.34 (1.12–1.59)* | 1.33 (1.11–1.59)* |
| **ApoB / ApoA1** | < 20 years | 0.89 (0.64–1.23) | 0.87 (0.62–1.22) |  | 1.15 (1.02–1.29)* | 1.09 (0.95–1.24) |  | 1.25 (1.14–1.36)* | 1.21 (1.10–1.33)* |
|  | ≥ 20 years | 1.36 (1.17–1.58)* | 1.33 (1.14–1.55)* |  | 1.03 (0.88–1.20) | 0.94 (0.80–1.11) |  | 1.28 (1.13–1.45)* | 1.24 (1.09–1.41)* |
| **Lp(a)** | < 20 years | *1.12 (0.83–1.50)* | *1.12 (0.83–1.50)* |  | 1.13 (0.98–1.30) | 1.14 (0.99–1.31) |  | 1.13 (0.99–1.29) | 1.11 (0.98–1.27) |
|  | ≥ 20 years | 1.07 (0.89–1.29) | 1.06 (0.88–1.28) |  | 1.04 (0.90–1.20) | 1.03 (0.89–1.19) |  | 1.08 (0.89–1.30) | 1.07 (0.89–1.30) |
| **Triglycerides** | < 20 years | 0.88 (0.64–1.20) | 0.85 (0.61–1.18) |  | 1.27 (1.10–1.48)* | 1.16 (0.996–1.36) |  | 1.42 (1.24–1.62)* | 1.35 (1.17–1.55)* |
|  | ≥ 20 years | 1.19 (0.98–1.45) | 1.12 (0.92–1.38) |  | 1.12 (0.96–1.31) | 1.03 (0.87–1.21) |  | 1.37 (1.13–1.67)* | 1.30 (1.06–1.60)* |
| **AIP** | < 20 years | 0.89 (0.65–1.22) | 0.86 (0.62–1.20) |  | 1.34 (1.15–1.55)* | 1.23 (1.05–1.44)* |  | 1.47 (1.28–1.68)* | 1.38 (1.20–1.59)* |
|  | ≥ 20 years | 1.25 (1.02–1.51)* | 1.16 (0.95–1.43) |  | 1.12 (0.96–1.31) | 1.00 (0.85–1.18) |  | 1.40 (1.15–1.70)* | 1.32 (1.07–1.63)* |

^a^For follow-up < 20 years, dementia N cases/N Total=48/2142, 203/2142 for mortality, 245/2142 for CHD. In the follow-up ≥ 20 years the corresponding numbers for dementia were 116/1907, for mortality 180/1761, and for CHD 114/1703.

^b^Results in italics are from analyses where the proportionality hazards assumption did not hold.

Model 1: adjusted for age (as time scale), ethnicity, marital status, education, socioeconomic position, and use of lipids-lowering drugs, allowing the baseline hazard to differ by birth-cohort (5-years groups).

Model 2: Model 1 + health-related behaviours (smoking, alcohol consumption, physical activity, diet, and obesity).

Abbreviations: TC: Total Cholesterol, LDL-C: Low-Density Lipoprotein cholesterol, HDL-C: High-Density Lipoprotein cholesterol; ApoA1: Apolipoprotein A1; ApoB: Apolipoprotein B; Lp(a): Lipoprotein (a); AIP: Atherogenic Index of Plasma.

**p<0.05*

**eTable 15. Association of lipids (1991-1993) with dementia, mortality, and coronary heart disease over the follow-up (until March 2019) in men, adjusting for use of lipids-lowering drugs as a time-varying measure.^a,b^**

|  |  | **Dementia** | |  | **Mortality** | |  | **Coronary Heart Disease** | |
| --- | --- | --- | --- | --- | --- | --- | --- | --- | --- |
| **LIPIDS (per 1 SD increase)** | **Length of follow-up** | **Model 1**  **HR (95% CI)** | **Model 2**  **HR (95% CI)** |  | **Model 1**  **HR (95% CI)** | **Model 2**  **HR (95% CI)** |  | **Model 1**  **HR (95% CI)** | **Model 2**  **HR (95% CI)** |
| **TC** | < 20 years | 0.85 (0.66–1.09) | 0.84 (0.65–1.09) |  | *1.11 (1.01–1.22)** | *1.09 (0.99–1.21)* |  | *1.24 (1.16–1.33)** | *1.24 (1.16–1.33)** |
|  | ≥ 20 years | *0.94 (0.79–1.11)* | *0.92 (0.78–1.09)* |  | 1.03 (0.93–1.15) | 1.01 (0.91–1.13) |  | 1.14 (1.01–1.28)* | 1.13 (1.004–1.27)* |
| **LDL-C** | < 20 years | 0.84 (0.65–1.07) | 0.4 (0.66–1.07) |  | *1.08 (0.98–1.19)* | *1.07 (0.97–1.17)* |  | *1.26 (1.17–1.36)** | *1.26 (1.17–1.35)** |
|  | ≥ 20 years | 0.90 (0.76–1.06) | 0.89 (0.75–1.05) |  | 1.01 (0.91–1.13) | 1.01 (0.90–1.12) |  | 1.14 (1.01–1.28)* | 1.13 (1.01–1.28)* |
| **HDL-C** | < 20 years | 0.94 (0.71–1.24) | 0.94 (0.70–1.26) |  | 0.82 (0.73–0.93)* | 0.85 (0.75–0.97)* |  | 0.73 (0.67–0.80)* | 0.75 (0.69–0.83)* |
|  | ≥ 20 years | 0.99 (0.83–1.18) | 0.98 (0.82–1.18) |  | 0.91 (0.81–1.02) | 0.91 (0.81–1.03) |  | 0.78 (0.68–0.90)* | 0.77 (0.66–0.89)* |
| **Non-HDL-C** | < 20 years | 0.86 (0.68–1.10) | 0.86 (0.67–1.09) |  | *1.17 (1.07–1.29)** | *1.14 (1.04–1.26)** |  | *1.34 (1.25–1.44)** | *1.33 (1.24–1.43)** |
|  | ≥ 20 years | 0.94 (0.79–1.11) | *0.92 (0.78–1.10)* |  | 1.06 (0.95–1.18) | 1.04 (0.93–1.16) |  | 1.22 (1.09–1.37)* | 1.21 (1.08–1.36)* |
| **TC / HDL-C** | < 20 years | 0.93 (0.74–1.17) | 0.91 (0.72–1.16) |  | 1.29 (1.18–1.41)* | 1.25 (1.14–1.37)* |  | *1.41 (1.32–1.51)** | *1.39 (1.30–1.49)** |
|  | ≥ 20 years | 0.99 (0.84–1.16) | 0.98 (0.83–1.16) |  | 1.11 (1.01–1.23)* | 1.10 (0.99–1.21) |  | 1.27 (1.14–1.42)* | 1.28 (1.14–1.43)* |
| **LDL-C / HDL-C** | < 20 years | 0.90 (0.71–1.15) | 0.89 (0.69–1.14) |  | 1.26 (1.15–1.38)* | 1.23 (1.12–1.35)* |  | *1.41 (1.32–1.51)** | *1.39 (1.30–1.49)** |
|  | ≥ 20 years | 0.95 (0.81–1.12) | 0.95 (0.80–1.13) |  | 1.09 (0.99–1.21) | 1.08 (0.97–1.20) |  | 1.26 (1.12–1.41)* | 1.27 (1.13–1.42)* |
| **ApoA1** | < 20 years | 0.90 (0.68–1.19) | 0.89 (0.66–1.18) |  | *0.90 (0.80–1.01)* | 0.91 (0.81–1.03) |  | 0.81 (0.75–0.89)* | 0.83 (0.76–0.91)* |
|  | ≥ 20 years | 1.01 (0.85–1.19) | 0.99 (0.83–1.18) |  | 0.93 (0.83–1.04) | 0.92 (0.81–1.03) |  | 0.89 (0.77–1.02) | 0.87 (0.75–1.01) |
| **ApoB** | < 20 years | 0.90 (0.72–1.13) | 0.89 (0.71–1.13) |  | *1.21 (1.11–1.33)** | *1.17 (1.07–1.29)** |  | *1.34 (1.25–1.44)** | *1.33 (1.24–1.42)** |
|  | ≥ 20 years | *1.01 (0.86–1.18)* | *0.99 (0.84–1.16)* |  | 1.06 (0.96–1.18) | 1.04 (0.93–1.16) |  | 1.22 (1.08–1.36)* | 1.20 (1.07–1.35)* |
| **ApoB / ApoA1** | < 20 years | 0.96 (0.77–1.21) | 0.95 (0.75–1.20) |  | 1.25 (1.15–1.36)* | 1.22 (1.12–1.33)* |  | *1.35 (1.25–1.46)** | *1.33 (1.24–1.44)** |
|  | ≥ 20 years | 1.01 (0.85–1.19) | 1.00 (0.84–1.19) |  | 1.14 (1.01–1.29)* | 1.13 (0.99–1.28) |  | 1.24 (1.11–1.40)* | 1.25 (1.11–1.40)* |
| **Lp(a)** | < 20 years | 1.08 (0.86–1.36) | 1.07 (0.84–1.35) |  | 1.06 (0.97–1.16) | 1.07 (0.98–1.17) |  | 1.17 (1.09–1.26)* | 1.19 (1.10–1.27)* |
|  | ≥ 20 years | 1.01 (0.87–1.16) | 1.01 (0.87–1.16) |  | 1.04 (0.95–1.14) | 1.05 (0.95–1.15) |  | *1.06 (0.94–1.19)* | *1.05 (0.94–1.18)* |
| **Triglycerides** | < 20 years | 1.10 (0.88–1.37) | 1.08 (0.85–1.36) |  | 1.33 (1.22–1.46)* | 1.27 (1.15–1.39)* |  | *1.31 (1.22–1.41)** | 1.28 (1.19–1.38)* |
|  | ≥ 20 years | 1.09 (0.93–1.26) | 1.06 (0.91–1.23) |  | 1.15 (1.04–1.27)* | 1.10 (0.995–1.22) |  | 1.31 (1.17–1.47)* | 1.29 (1.15–1.45)* |
| **AIP** | < 20 years | 1.10 (0.88–1.38) | 1.08 (0.86–1.36) |  | 1.34 (1.23–1.47)* | 1.28 (1.16–1.41)* |  | *1.36 (1.26–1.46)** | 1.32 (1.23–1.43)* |
|  | ≥ 20 years | 1.07 (0.92–1.25) | 1.05 (0.90–1.24) |  | 1.15 (1.04–1.27)* | 1.11 (1.003–1.23)* |  | 1.32 (1.18–1.48)* | 1.32 (1.17–1.48)* |

^a^For follow-up < 20 years, dementia N cases/ N Total=77/5306, 504/5306 for mortality, 824/5306 for CHD. In the follow-up ≥ 20 years the corresponding numbers for dementia were 205/4758, for mortality 448/4802, and for CHD 307/4088.

^b^Results in italics are from analyses where the proportionality hazards assumption did not hold.

Model 1: adjusted for age (as time scale), ethnicity, marital status, education, socioeconomic position, and use of lipids-lowering drugs, allowing the baseline hazard to differ by birth-cohort (5-years groups).

Model 2: Model 1 + health-related behaviours (smoking, alcohol consumption, physical activity, diet, and obesity).

Abbreviations: TC: Total Cholesterol, LDL-C: Low-Density Lipoprotein cholesterol, HDL-C: High-Density Lipoprotein cholesterol; ApoA1: Apolipoprotein A1; ApoB: Apolipoprotein B; Lp(a): Lipoprotein (a); AIP: Atherogenic Index of Plasma.

**p<0.05*

**eTable 16. Association of lipids (1991-1993) with dementia, mortality, and coronary heart disease over the follow-up (until March 2019) in women, adjusting for use of lipids-lowering drugs as a time-varying measure.^a,b^**

|  |  | **Dementia** | |  | **Mortality** | |  | **Coronary Heart Disease** | |
| --- | --- | --- | --- | --- | --- | --- | --- | --- | --- |
| **LIPIDS (per 1 SD increase)** | **Length of follow-up** | **Model 1**  **HR (95% CI)** | **Model 2**  **HR (95% CI)** |  | **Model 1**  **HR (95% CI)** | **Model 2**  **HR (95% CI)** |  | **Model 1**  **HR (95% CI)** | **Model 2**  **HR (95% CI)** |
| **TC** | < 20 years | 0.90 (0.66–1.23) | 0.90 (0.65–1.24) |  | 0.97 (0.84–1.13) | 0.95 (0.82–1.11) |  | 1.18 (1.05–1.33)* | 1.19 (1.06–1.34)* |
|  | ≥ 20 years | 1.28 (1.08–1.50)* | 1.29 (1.09–1.52)* |  | *1.00 (0.88–1.15)* | *1.00 (0.88–1.14)* |  | 1.25 (1.05–1.48)* | 1.25 (1.05–1.50)* |
| **LDL-C** | < 20 years | 0.90 (0.68–1.20) | 0.90 (0.67–1.21) |  | 1.01 (0.88–1.17) | 0.99 (0.86–1.14) |  | *1.21 (1.08–1.35)** | *1.20 (1.07–1.34)** |
|  | ≥ 20 years | 1.32 (1.13–1.54)* | 1.33 (1.14–1.55)* |  | 0.99 (0.88–1.13) | 0.98 (0.86–1.11) |  | 1.29 (1.09–1.52)* | 1.29 (1.09–1.52)* |
| **HDL-C** | < 20 years | 1.07 (0.84–1.37) | 1.11 (0.86–1.43) |  | 0.77 (0.67–0.88)* | 0.81 (0.70–0.93)* |  | *0.78 (0.68–0.89)** | *0.84 (0.73–0.96)** |
|  | ≥ 20 years | 0.79 (0.66–0.95)* | 0.84 (0.70–1.01) |  | *0.93 (0.82–1.07)* | *1.01 (0.88–1.16)* |  | 0.78 (0.65–0.94)* | 0.83 (0.69–0.998)* |
| **Non-HDL-C** | < 20 years | 0.88 (0.66–1.18) | 0.87 (0.64–1.18) |  | 1.07 (0.93–1.23) | 1.02 (0.89–1.18) |  | *1.27 (1.14–1.42)** | *1.24 (1.11–1.39)** |
|  | ≥ 20 years | 1.36 (1.17–1.58)* | 1.35 (1.15–1.57)* |  | 1.03 (0.90–1.17) | 1.00 (0.87–1.14) |  | 1.34 (1.14–1.58)* | 1.32 (1.12–1.57)* |
| **TC / HDL-C** | < 20 years | 0.80 (0.58–1.11) | 0.76 (0.53–1.08) |  | 1.15 (1.04–1.28)* | 1.09 (0.97–1.22) |  | 1.23 (1.12–1.34)* | 1.19 (1.09–1.30)* |
|  | ≥ 20 years | 1.29 (1.14–1.45)* | 1.24 (1.10–1.40)* |  | 1.08 (0.94–1.24) | 0.98 (0.84–1.16) |  | 1.21 (1.09–1.34)* | 1.16 (1.03–1.30)* |
| **LDL-C / HDL-C** | < 20 years | 0.801 (0.59–1.11) | 0.78 (0.55–1.09) |  | 1.13 (1.02–1.24)* | 1.07 (0.96–1.19) |  | 1.20 (1.10–1.31)* | 1.17 (1.08–1.28)* |
|  | ≥ 20 years | 1.27 (1.13–1.44)* | 1.24 (1.10–1.40)* |  | 1.05 (0.91–1.20) | 0.97 (0.83–1.13) |  | 1.19 (1.08–1.32)* | 1.15 (1.03–1.28)* |
| **ApoA1** | < 20 years | 1.08 (0.82–1.42) | 1.11 (0.84–1.47) |  | 0.82 (0.72–0.93)* | 0.84 (0.74–0.96)* |  | 0.84 (0.74–0.95)* | 0.90 (0.79–1.02) |
|  | ≥ 20 years | 0.82 (0.68–0.99)* | 0.87 (0.72–1.05) |  | *0.99 (0.86–1.14)* | *1.05 (0.91–1.21)* |  | 0.80 (0.67–0.96)* | 0.84 (0.70–1.02) |
| **ApoB** | < 20 years | 0.89 (0.66–1.20) | 0.87 (0.64–1.19) |  | 1.07 (0.93–1.23) | 1.01 (0.88–1.17) |  | *1.26 (1.13–1.42)** | *1.24 (1.10–1.39)** |
|  | ≥ 20 years | 1.40 (1.19–1.64)* | 1.37 (1.16–1.61)* |  | 1.04 (0.91–1.19) | 1.00 (0.87–1.15) |  | 1.41 (1.17–1.69)* | 1.39 (1.15–1.68)* |
| **ApoB / ApoA1** | < 20 years | 0.84 (0.60–1.16) | 0.80 (0.56–1.14) |  | 1.13 (1.01–1.27)* | 1.07 (0.94–1.21) |  | 1.24 (1.13–1.37)* | 1.20 (1.09–1.33)* |
|  | ≥ 20 years | 1.37 (1.18–1.59)* | 1.34 (1.16–1.55)* |  | 1.05 (0.91–1.22) | 0.97 (0.83–1.14) |  | 1.29 (1.13–1.47)* | 1.24 (1.08–1.42)* |
| **Lp(a)** | < 20 years | 1.04 (0.78–1.39) | 1.04 (0.78–1.39) |  | 1.10 (0.96–1.26) | 1.09 (0.96–1.25) |  | 1.15 (1.01–1.30)* | 1.13 (0.99–1.28) |
|  | ≥ 20 years | 1.07 (0.90–1.28) | 1.05 (0.88–1.26) |  | 1.07 (0.93–1.23) | 1.06 (0.92–1.22) |  | 1.11 (0.91–1.37) | 1.11 (0.90–1.37) |
| **Triglycerides** | < 20 years | 0.91 (0.68–1.22) | 0.86 (0.63–1.18) |  | 1.27 (1.09–1.48)* | 1.15 (0.98–1.36) |  | *1.36 (1.20–1.55)** | *1.29 (1.13–1.48)** |
|  | ≥ 20 years | 1.24 (1.03–1.49)* | 1.15 (0.95–1.40) |  | 1.14 (0.98–1.33) | 1.05 (0.89–1.24) |  | 1.36 (1.13–1.64)* | 1.27 (1.05–1.54)* |
| **AIP** | < 20 years | 0.90 (0.67–1.21) | 0.85 (0.62–1.17) |  | 1.33 (1.15–1.54)* | 1.21 (1.03–1.43)* |  | *1.41 (1.24–1.61)** | *1.32 (1.15–1.52)** |
|  | ≥ 20 years | 1.30 (1.08–1.56)* | 1.20 (0.99–1.46) |  | 1.14 (0.98–1.34) | 1.03 (0.87–1.22) |  | 1.39 (1.16–1.66)* | 1.30 (1.07–1.57)* |

^a^For follow-up < 20 years, dementia N cases/ N Total=54/2366, 222/2366 for mortality, 263/2366 for CHD. In the follow-up ≥ 20 years the corresponding numbers for dementia were 126/2109, for mortality 216/2144, and for CHD 119/1891.

^b^Results in italics are from analyses where the proportionality hazards assumption did not hold.

Model 1: adjusted for age (as time scale), ethnicity, marital status, education, socioeconomic position, and use of lipids-lowering drugs, allowing the baseline hazard to differ by birth-cohort (5-years groups).

Model 2: Model 1 + health-related behaviours (smoking, alcohol consumption, physical activity, diet, and obesity).

Abbreviations: TC: Total Cholesterol, LDL-C: Low-Density Lipoprotein cholesterol, HDL-C: High-Density Lipoprotein cholesterol; ApoA1: Apolipoprotein A1; ApoB: Apolipoprotein B; Lp(a): Lipoprotein (a); AIP: Atherogenic Index of Plasma.

**p<0.05*

**eTable 17. Association of lipids (1991-1993) with dementia, mortality, and coronary heart disease over the follow-up (until March 2019) stratified by the length of follow-up in women in models further adjusted for menopausal status.^a,b^**

|  |  |  | **Dementia** | |  | **Mortality** | |  | **Coronary Heart Disease** | |
| --- | --- | --- | --- | --- | --- | --- | --- | --- | --- | --- |
| **LIPIDS (per 1 SD increase)** | **Follow-up** |  | **Model 1**  **HR (95% CI)** | **Model 2**  **HR (95% CI)** |  | **Model 1**  **HR (95% CI)** | **Model 2**  **HR (95% CI)** |  | **Model 1**  **HR (95% CI)** | **Model 2**  **HR (95% CI)** |
| **TC** | < 20 years |  | 0.88 (0.67–1.16) | 0.89 (0.67–1.18) |  | 0.95 (0.83–1.09) | 0.95 (0.83–1.09) |  | 1.20 (1.07–1.35)* | 1.18 (1.05–1.33)* |
|  | ≥ 20 years |  | 1.30 (1.11–1.52)* | 1.29 (1.09–1.51)* |  | *0.98 (0.86–1.12)* | *0.97 (0.85–1.12)* |  | 1.28 (1.08–1.52)* | 1.25 (1.05–1.49)* |
| **LDL-C** | < 20 years |  | 0.88 (0.67–1.15) | 0.89 (0.68–1.16) |  | 0.99 (0.87–1.13) | 0.99 (0.87–1.12) |  | *1.21 (1.08–1.35)** | 1.20 (1.07–1.34)* |
|  | ≥ 20 years |  | 1.33 (1.14–1.55)* | 1.32 (1.13–1.54)* |  | 0.96 (0.85–1.10) | 0.96 (0.84–1.09) |  | 1.31 (1.11–1.54)* | 1.28 (1.09–1.52)* |
| **HDL-C** | < 20 years |  | 1.10 (0.85–1.43) | 1.10 (0.85–1.43) |  | 0.81 (0.71–0.94)* | 0.81 (0.71–0.94)* |  | *0.83 (0.73–0.95)** | *0.83 (0.73–0.94)** |
|  | ≥ 20 years |  | 0.84 (0.70–1.004) | 0.84 (0.70–1.002) |  | *1.01 (0.89–1.16)* | *1.01 (0.88–1.16)* |  | 0.82 (0.68–0.999)* | 0.82 (0.68–0.99)* |
| **Non-HDL-C** | < 20 years |  | 0.85 (0.65–1.12) | 0.86 (0.66–1.14) |  | 1.02 (0.89–1.16) | 1.02 (0.89–1.16) |  | *1.25 (1.12–1.41)** | *1.24 (1.11–1.39)** |
|  | ≥ 20 years |  | 1.34 (1.15–1.57)* | 1.33 (1.14–1.56)* |  | 0.98 (0.86–1.12) | 0.97 (0.85–1.11) |  | 1.34 (1.13–1.58)* | 1.31 (1.10–1.55)* |
| **TC / HDL-C** | < 20 years |  | 0.76 (0.53–1.09) | 0.77 (0.54–1.10) |  | 1.08 (0.95–1.23) | 1.08 (0.95–1.23) |  | 1.20 (1.09–1.31)* | 1.19 (1.09–1.31)* |
|  | ≥ 20 years |  | 1.25 (1.08–1.44)* | 1.24 (1.08–1.44)* |  | 0.97 (0.83–1.14) | 0.97 (0.82–1.13) |  | 1.17 (1.03–1.32)* | 1.16 (1.02–1.32)* |
| **LDL-C / HDL-C** | < 20 years |  | 0.77 (0.55–1.09) | 0.78 (0.55–1.10) |  | 1.06 (0.94–1.21) | 1.06 (0.93–1.21) |  | 1.18 (1.08–1.28)* | 1.17 (1.08–1.28)* |
|  | ≥ 20 years |  | 1.25 (1.09–1.43)* | 1.24 (1.08–1.43)* |  | 0.95 (0.81–1.11) | 0.95 (0.81–1.11) |  | 1.15 (1.03–1.29)* | 1.15 (1.02–1.29)* |
| **ApoA1** | < 20 years |  | 1.09 (0.83–1.44) | 1.09 (0.83–1.44) |  | 0.85 (0.74–0.97)* | 0.85 (0.73–0.97)* |  | 0.89 (0.79–1.01) | 0.89 (0.78–1.01) |
|  | ≥ 20 years |  | 0.86 (0.71–1.03) | 0.86 (0.71–1.03) |  | *1.04 (0.91–1.20)* | *1.04 (0.91–1.20)* |  | 0.85 (0.70–1.03) | 0.84 (0.69–1.01) |
| **ApoB** | < 20 years |  | 0.86 (0.65–1.14) | 0.87 (0.66–1.15) |  | 1.01 (0.88–1.15) | 1.00 (0.88–1.15) |  | *1.25 (1.11–1.40)** | *1.24 (1.10–1.39)** |
|  | ≥ 20 years |  | 1.37 (1.16–1.61)* | 1.36 (1.15–1.60)* |  | 0.98 (0.85–1.12) | 0.97 (0.85–1.12) |  | 1.40 (1.18–1.68)* | 1.38 (1.15–1.65)* |
| **ApoB / ApoA1** | < 20 years |  | 0.81 (0.58–1.12) | 0.81 (0.58–1.13) |  | 1.06 (0.93–1.22) | 1.06 (0.92–1.22) |  | 1.21 (1.10–1.33)* | 1.21 (1.10–1.33)* |
|  | ≥ 20 years |  | 1.34 (1.15–1.56)* | 1.34 (1.15–1.56)* |  | 0.96 (0.82–1.12) | 0.96 (0.82–1.12) |  | 1.25 (1.10–1.41)* | 1.25 (1.10–1.41)* |
| **Lp(a)** | < 20 years |  | 1.05 (0.80–1.39) | 1.07 (0.81–1.41) |  | 1.10 (0.96–1.26) | 1.10 (0.96–1.26) |  | 1.13 (0.996–1.28) | 1.12 (0.99–1.27) |
|  | ≥ 20 years |  | 1.06 (0.89–1.27) | 1.05 (0.88–1.26) |  | 1.05 (0.92–1.21) | 1.05 (0.91–1.21) |  | 1.10 (0.91–1.33) | 1.09 (0.90–1.32) |
| **Triglycerides** | < 20 years |  | 0.86 (0.63–1.19) | 0.87 (0.64–1.20) |  | 1.14 (0.98–1.33) | 1.14 (0.98–1.33) |  | 1.32 (1.15–1.51)* | 1.33 (1.16–1.51)* |
|  | ≥ 20 years |  | 1.17 (0.96–1.42) | 1.16 (0.96–1.41) |  | 1.03 (0.88–1.20) | 1.02 (0.88–1.20) |  | 1.29 (1.05–1.58)* | 1.27 (1.03–1.55)* |
| **AIP** | < 20 years |  | 0.85 (0.62–1.17) | 0.86 (0.62–1.18) |  | 1.20 (1.03–1.40)* | 1.20 (1.02–1.40)* |  | *1.34 (1.17–1.54)** | *1.33 (1.16–1.53)** |
|  | ≥ 20 years |  | 1.22 (0.9998–1.48) | 1.21 (0.99–1.47) |  | 1.02 (0.87–1.19) | 1.01 (0.86–1.19) |  | 1.31 (1.07–1.61)* | 1.29 (1.06–1.59)* |

^a^For follow-up < 20 years, dementia N cases/ N Total=53/2348, 218/2348 for mortality, 262/2348 for CHD. In the follow-up ≥ 20 years the corresponding numbers for dementia were 126/2096, for mortality 212/2130, and for CHD 118/1879.

^b^Results in italics are from analyses where the proportionality hazards assumption did not hold.

Model 1: adjusted for age (as time scale), ethnicity, marital status, education, socioeconomic position, use of lipids-lowering drugs, and health-related behaviours (smoking, alcohol consumption, physical activity, diet, and obesity), allowing the baseline hazard to differ by birth-cohort (5-years groups).

Model 2: Model 1 + menopausal status.

Abbreviations: TC: Total Cholesterol, LDL-C: Low-Density Lipoprotein cholesterol, HDL-C: High-Density Lipoprotein cholesterol; ApoA1: Apolipoprotein A1; ApoB: Apolipoprotein B; Lp(a): Lipoprotein (a); AIP: Atherogenic Index of Plasma.

* *p<0.05*

**eTable 18. Estimated marginal mean (cases – non-cases) trajectories of blood lipids over 28 years before dementia, mortality, and coronary heart disease in men and women using a backward timescale.^a^**

|  | | **DEMENTIA** | | | | **MORTALITY** | | | | **CORONARY HEART DISEASE** | | | |
| --- | --- | --- | --- | --- | --- | --- | --- | --- | --- | --- | --- | --- | --- |
|  | | **MEN** | | **WOMEN** | | **MEN** | | **WOMEN** | | **MEN** | | **WOMEN** | |
| **LIPIDS** | **Time**  **(years)** | **Difference**  **(95% CI)** | ***p* value** | **Difference**  **(95% CI)** | ***p* value** | **Difference**  **(95% CI)** | ***p* value** | **Difference**  **(95% CI)** | ***p* value** | **Difference**  **(95% CI)** | ***p* value** | **Difference**  **(95% CI)** | ***p* value** |
| **TC** | -25 | 0.02 (-0.11–0.15) | 0.3227 | 0.41 (0.22–0.60) | <0.0001 | 0.23 (0.14–0.32) | <0.0001 | 0.17 (0.01–0.32) | 0.0372 | 0.33 (0.23–0.43) | <0.0001 | 0.43 (0.24–0.61) | <0.0001 |
|  | -20 | 0.03 (-0.07–0.13) | 0.5365 | 0.28 (0.13–0.44) | 0.0002 | 0.20 (0.13–0.27) | <0.0001 | 0.19 (0.06–0.32) | 0.0039 | 0.32 (0.24–0.39) | <0.0001 | 0.32 (0.19–0.46) | <0.0001 |
|  | -15 | 0.01 (-0.08–0.11) | 0.8116 | 0.18 (0.04–0.31) | 0.0099 | 0.16 (0.10–0.23) | <0.0001 | 0.15 (0.04–0.26) | 0.0053 | 0.30 (0.24–0.37) | <0.0001 | 0.25 (0.13–0.36) | <0.0001 |
|  | -10 | 0.00 (-0.09–0.09) | 0.9390 | 0.11 (-0.03–0.24) | 0.1303 | 0.13 (0.07–0.18) | <0.0001 | 0.09 (-0.01–0.19) | 0.0826 | 0.29 (0.23–0.35) | <0.0001 | 0.21 (0.10–0.32) | 0.0001 |
|  | -5 | 0.01 (-0.08–0.10) | 0.8685 | 0.09 (-0.05–0.24) | 0.2199 | 0.08 (0.03–0.14) | 0.0047 | 0.04 (-0.06–0.15) | 0.4188 | 0.28 (0.22–0.33) | <0.0001 | 0.23 (0.12–0.34) | <0.0001 |
|  | 0 | 0.06 (-0.06–0.19) | 0.7116 | 0.15 (-0.13–0.44) | 0.3006 | 0.04 (-0.05–0.12) | 0.3773 | 0.06 (-0.16–0.27) | 0.6027 | 0.27 (0.19–0.34) | <0.0001 | 0.31 (0.12–0.51) | 0.0020 |
| **LDL-C** | -25 | 0.05 (-0.08–0.18) | 0.4537 | 0.49 (0.30–0.68) | <0.0001 | 0.30 (0.20–0.40) | <0.0001 | 0.30 (0.14–0.46) | 0.0002 | 0.36 (0.25–0.46) | <0.0001 | 0.52 (0.33–0.70) | <0.0001 |
|  | -20 | 0.05 (-0.06–0.16) | 0.4070 | 0.41 (0.26–0.57) | <0.0001 | 0.30 (0.22–0.38) | <0.0001 | 0.33 (0.19–0.46) | <0.0001 | 0.42 (0.33–0.50) | <0.0001 | 0.43 (0.28–0.57) | <0.0001 |
|  | -15 | 0.03 (-0.06–0.13) | 0.4890 | 0.31 (0.17–0.45) | <0.0001 | 0.24 (0.17–0.31) | <0.0001 | 0.28 (0.17–0.39) | <0.0001 | 0.40 (0.33–0.47) | <0.0001 | 0.35 (0.23–0.48) | <0.0001 |
|  | -10 | 0.01 (-0.08–0.11) | 0.7872 | 0.20 (0.06–0.34) | 0.0046 | 0.16 (0.10–0.22) | <0.0001 | 0.20 (0.09–0.30) | 0.0002 | 0.35 (0.29–0.42) | <0.0001 | 0.32 (0.21–0.43) | <0.0001 |
|  | -5 | -0.02 (-0.11–0.08) | 0.6932 | 0.14 (-0.01–0.29) | 0.0660 | 0.010 (0.04–0.16) | 0.0013 | 0.10 (-0.002–0.21) | 0.0551 | 0.32 (0.26–0.38) | <0.0001 | 0.33 (0.22–0.45) | <0.0001 |
|  | 0 | -0.06 (-0.23–0.10) | 0.4421 | 0.14 (-0.14–0.43) | 0.3256 | 0.09 (-0.02–0.20) | 0.1050 | 0.04 (-0.18–0.25) | 0.7238 | 0.33 (0.23–0.43) | <0.0001 | 0.41 (0.21–0.61) | 0.0001 |
| **HDL-C** | -25 | -0.02 (-0.14–0.09) | 0.6658 | -0.24 (-0.43–-0.06) | 0.0102 | -0.18 (-0.26–-0.09) | <0.0001 | -0.31 (-0.47–-0.16) | 0.0001 | -0.25 (-0.34–-0.16) | 0.0024 | -0.25 (-0.43–-0.08) | 0.0049 |
|  | -20 | -0.08 (-0.18–0.03) | 0.1570 | -0.24 (-0.41–-0.08) | 0.0034 | -0.25 (-0.32–-0.17) | <0.0001 | -0.40 (-0.54–-0.26) | <0.0001 | -0.26 (-0.33–-0.18) | <0.0001 | -0.31 (-0.45–-0.17) | <0.0001 |
|  | -15 | -0.05 (-0.16–0.05) | 0.3388 | -0.25 (-0.41–-0.09) | 0.0022 | -0.22 (-0.29–-0.15) | <0.0001 | -0.9 (-0.51–-0.26) | <0.0001 | -0.25 (-0.32–-0.19) | <0.0001 | -0.35 (-0.49–-0.22) | <0.0001 |
|  | -10 | 0.04 (-0.08–0.15) | 0.5384 | -0.24 (-0.41–-0.06) | 0.0077 | -0.15 (-0.22–-0.08) | <0.0001 | -0.32 (-0.45–-0.20) | <0.0001 | -0.24 (-0.31–-0.17) | <0.0001 | -0.37 (-0.50–-0.23) | <0.0001 |
|  | -5 | 0.17 (0.05–0.29) | 0.0056 | -0.18 (-0.37–0.01) | 0.0648 | -0.09 (-0.17–-0.01) | 0.0193 | -0.26 (-0.40–-0.13) | 0.0001 | -0.21 (-0.28–-0.14) | <0.0001 | -0.33 (-0.47–-0.19) | <0.0001 |
|  | 0 | 0.35 (0.17–0.52) | 0.0001 | -0.06 (-0.38–0.26) | 0.7253 | -0.11 (-0.23–0.001) | 0.0670 | -0.25 (-0.49–-0.02) | 0.0365 | -0.16 (-0.27–-0.06) | 0.0024 | -0.22 (-0.45–0.005) | 0.0549 |
| **Non-HDL-C** | -25 | 0.05 (-0.09–0.18) | 0.4902 | 0.48 (0.30–0.67) | <0.0001 | 0.29 (0.19–0.39) | <0.0001 | 0.28 (0.13–0.44) | 0.0003 | 0.39 (0.28–0.49) | <0.0001 | 0.51 (0.33–0.69) | <0.0001 |
|  | -20 | 0.05 (-0.06–0.16) | 0.3571 | 0.36 (0.21–0.52) | <0.0001 | 0.28 (0.20–0.37) | <0.0001 | 0.31 (0.18–0.44) | <0.0001 | 0.41 (0.33–0.50) | <0.0001 | 0.43 (0.29–0.56) | <0.0001 |
|  | -15 | 0.03 (-0.06–0.13) | 0.4979 | 0.26 (0.12–0.40) | 0.0002 | 0.23 (0.17–0.30) | <0.0001 | 0.27 (0.17–0.38) | <0.0001 | 0.39 (0.32–0.46) | <0.0001 | 0.37 (0.25–0.48) | <0.0001 |
|  | -10 | 0.00 (-0.10–0.09) | 0.9639 | 0.19 (0.05–0.32) | 0.0077 | 0.16 (0.11–0.22) | <0.0001 | 0.20 (0.10–0.30) | 0.0001 | 0.35 (0.29–0.41) | <0.0001 | 0.34 (0.23–0.45) | <0.0001 |
|  | -5 | -0.05 (-0.15–0.04) | 0.2873 | 0.15 (0.01–0.30) | 0.0348 | 0.11 (0.05–0.17) | 0.0003 | 0.14 (0.04–0.24) | 0.0084 | 0.32 (0.26–0.38) | <0.0001 | 0.35 (0.24–0.46) | <0.0001 |
|  | 0 | -0.11 (-0.27–0.05) | 0.1793 | 0.18 (0.10–0.45) | 0.2027 | 0.09 (-0.02–0.20) | 0.0941 | 0.12 (-0.09–0.32) | 0.2659 | 0.35 (0.25–0.45) | <0.0001 | 0.40 (0.21–0.59) | <0.0001 |
| **TC / HDL-C** | -25 | 0.03 (-0.09–0.15) | 0.6373 | 0.37 (0.20–0.55) | <0.0001 | 0.38 (0.29–0.47) | <0.0001 | 0.24 (0.10–0.38) | 0.0009 | 0.24 (0.16–0.32) | <0.0001 | 0.30 (0.11–0.48) | 0.0018 |
|  | -20 | 0.05 (-0.05–0.15) | 0.3439 | 0.25 (0.12–0.38) | 0.0001 | 0.37 (0.29–0.44) | <0.0001 | 0.25 (0.15–0.35) | <0.0001 | 0.45 (0.38–0.53) | <0.0001 | 0.25 (0.11–0.39) | 0.0003 |
|  | -15 | 0.03 (-0.06–0.12) | 0.4975 | 0.17 (0.05–0.29) | 0.0065 | 0.30 (0.24–0.36) | <0.0001 | 0.24 (0.14–0.34) | <0.0001 | 0.40 (0.34–0.47) | <0.0001 | 0.27 (0.16–0.38) | <0.0001 |
|  | -10 | -0.02 (-0.10–0.07) | 0.6956 | 0.13 (0.02–0.24) | 0.0181 | 0.21 (0.16–0.26) | <0.0001 | 0.21 (0.13–0.30) | <0.0001 | 0.35 (0.29–0.41) | <0.0001 | 0.30 (0.19–0.40) | <0.0001 |
|  | -5 | -0.08 (-0.17–-0.003) | *0.0420* | 0.14 (0.03–0.26) | 0.0156 | 0.14 (0.09–0.19) | <0.0001 | 0.16 (0.08–0.24) | 0.0002 | 0.29 (0.24–0.35) | <0.0001 | 0.28 (0.18–0.37) | <0.0001 |
|  | 0 | -0.16 (-0.30–-0.03) | 0.0156 | 0.20 (-0.01–0.41) | 0.0681 | 0.13 (0.04–0.22) | 0.0047 | 0.09 (-0.07–0.25) | 0.2629 | 0.49 (0.39–0.59) | <0.0001 | 0.16 (-0.05–0.38) | 0.1394 |

|  | | **DEMENTIA** | | | | **MORTALITY** | | | | **CORONARY HEART DISEASE** | | | | |
| --- | --- | --- | --- | --- | --- | --- | --- | --- | --- | --- | --- | --- | --- | --- |
|  | | **MEN** | | **WOMEN** | | **MEN** | | **WOMEN** | | **MEN** | | | **WOMEN** | |
| **LIPIDS** | **Time**  **(years)** | **Difference**  **(95% CI)** | ***p* value** | **Difference**  **(95% CI)** | ***p* value** | **Difference**  **(95% CI)** | ***p* value** | **Difference**  **(95% CI)** | ***p* value** | **Difference**  **(95% CI)** | ***p* value** | **Difference**  **(95% CI)** | | ***p* value** |
| **LDL-C / HDL-C** | -25 | 0.03 (-0.09–0.15) | 0.6327 | 0.29 (0.09–0.48) | 0.0043 | 0.41 (0.31–0.50) | <0.0001 | 0.12 (-0.04–0.29) | 0.1324 | 0.49 (0.39–0.59) | <0.0001 | 0.18 (-0.05–0.40) | | 0.0020 |
|  | -20 | 0.04 (-0.06–0.14) | 0.4540 | 0.21 (0.06–0.37) | 0.0075 | 0.40 (0.32–0.47) | <0.0001 | 0.17 (0.03–0.31) | 0.0151 | 0.47 (0.40–0.55) | <0.0001 | 0.26 (0.12–0.41) | | 0.0003 |
|  | -15 | 0.03 (-0.06–0.12) | 0.5625 | 0.18 (0.05–0.31) | 0.0072 | 0.32 (0.26–0.38) | <0.0001 | 0.20 (0.09–0.31) | 0.0003 | 0.43 (0.37–0.50) | <0.0001 | 0.28 (0.16–0.40) | | <0.0001 |
|  | -10 | -0.01 (-0.09–0.08) | 0.8489 | 0.16 (0.03–0.29) | 0.0144 | 0.22 (0.17–0.27) | <0.0001 | 0.20 (0.10–0.29) | 0.0001 | 0.38 (0.32–0.44) | <0.0001 | 0.31 (0.20–0.42) | | <0.0001 |
|  | -5 | -0.06 (-0.14–0.02) | 0.1191 | 0.13 (0.01–0.26) | *0.0386* | 0.14 (0.09–0.19) | <0.0001 | 0.13 (0.04–0.22) | 0.0040 | 0.31 (0.26–0.36) | <0.0001 | 0.29 (0.19–0.39) | | <0.0001 |
|  | 0 | -0.14 (-0.27–-0.01*)* | *0.0388* | 0.07 (-0.25–0.39) | 0.6573 | 0.13 (0.04–0.22) | 0.0048 | -0.01 (-0.25–0.24) | 0.9618 | 0.23 (0.14–0.31) | <0.0001 | 0.31 (-0.11–0.51) | | 0.1338 |
| **Triglycerides** | -25 | 0.00 (-0.15–0.14) | 0.9659 | 0.16 (0.01–0.31) | *0.0399* | 0.05 (-0.06–0.16) | 0.3688 | 0.12 (0.01–0.24) | 0.0302 | 0.24 (0.12–0.36) | 0.0001 | 0.21 (0.08–0.34) | | 0.0021 |
|  | -20 | 0.02 (-0.10–0.14) | 0.7684 | 0.04 (-0.09–0.16) | 0.5774 | 0.04 (-0.05–0.13) | 0.3647 | 0.09 (0.00–0.18) | 0.0510 | 0.16 (0.07–0.24) | 0.0006 | 0.20 (0.10–0.30) | | 0.0001 |
|  | -15 | 0.02 (-0.09–0.13) | 0.7108 | 0.00 (-0.11–0.12) | 0.9784 | 0.06 (-0.01–0.13) | 0.1159 | 0.08 (0.00–0.17) | 0.0589 | 0.12 (0.04–0.19) | 0.0020 | 0.20 (0.10–0.29) | | 0.0001 |
|  | -10 | -0.01 (-0.12–0.10) | 0.8393 | 0.03 (-0.09–0.15) | 0.6345 | 0.08 (0.01–0.15) | 0.0186 | 0.11 (0.02–0.19) | 0.0111 | 0.12 (0.05–0.19) | 0.0006 | 0.18 (0.09–0.28) | | 0.0001 |
|  | -5 | -0.09 (-0.20–0.02) | 0.0933 | 0.09 (-0.03–0.22) | 0.1512 | 0.08 (0.02–0.15) | 0.0152 | 0.16 (0.07–0.25) | 0.0003 | 0.15 (0.08–0.22) | <0.0001 | 0.17 (0.08–0.27) | | 0.0004 |
|  | 0 | -0.23 (-0.42–-0.05) | 0.0148 | 0.17 (-0.08–0.41) | 0.1828 | 0.04 (-0.09–0.17) | 0.5276 | 0.25 (0.11–0.38) | 0.0003 | 0.20 (0.09–0.31) | 0.0006 | 0.16 (0.02–0.20) | | 0.0223 |
| **AIP** | -25 | 0.00 (-0.13–0.13) | 0.9962 | 0.19 (0.03–0.34) | 0.0190 | 0.11 (0.02–0.21) | 0.0218 | 0.11 (-0.02–0.24) | 0.0948 | 0.28 (0.17–0.38) | <0.0001 | 0.24 (0.10–0.39) | | 0.0012 |
|  | -20 | 0.03 (-0.08–0.15) | 0.5498 | 0.10 (-0.03–0.24) | 0.1406 | 0.15 (0.06–0.23) | 0.0004 | 0.15 (0.03–0.26) | 0.0129 | 0.23 (0.15–0.31) | <0.0001 | 0.27 (0.15–0.38) | | <0.0001 |
|  | -15 | 0.02 (-0.09–0.12) | 0.7324 | 0.10 (-0.03–0.23) | 0.1344 | 0.14 (0.07–0.21) | 0.0001 | 0.17 (0.07–0.27) | 0.0009 | 0.20 (0.13–0.27) | <0.0001 | 0.28 (0.18–0.39) | | <0.0001 |
|  | -10 | -0.04 (-0.15–0.06) | 0.4081 | 0.13 (-0.01–0.26) | 0.0617 | 0.11 (0.05–0.18) | 0.0010 | 0.18 (0.09–0.28) | 0.0002 | 0.18 (0.12–0.25) | <0.0001 | 0.28 (0.18–0.39) | | <0.0001 |
|  | -5 | -0.15 (-0.26–-0.04) | 0.0064 | 0.14 (0.001–0.29) | *0.0491* | 0.08 (0.02–0.15) | 0.0141 | 0.20 (0.10–0.30) | 0.0001 | 0.19 (0.12–0.25) | <0.0001 | 0.25 (0.14–0.36) | | <0.0001 |
|  | 0 | -0.30 (-0.47–-0.13) | 0.0005 | 0.10 (-0.15–0.35) | 0.4478 | 0.08 (-0.03–0.19) | 0.1710 | 0.22 (0.04–0.41) | 0.0194 | 0.20 (0.10–0.31) | 0.0001 | 0.17 (-0.002–0.35) | | 0.0533 |

^a^Estimated marginal means based on linear mixed models with a backward scale of time (time before occurrence of the event, in years, with t =0 defined as the time of the event or 31^st^ of March 2019, whichever came first). The analyses were adjusted for sex, use of lipids-lowering drugs (time-varying), ethnicity, marital status (time-varying), education, socioeconomic position (time-varying), health-related behaviours (smoking status, alcohol consumption, fruit and vegetable consumption, physical activity, and obesity, all time-varying), a binary indicator of data from 1991-1993 (pre-statin era), age at t =0, and event status at t = 0. These models included an interaction term between time and event status at t = 0 as well as between time and age at t = 0. Estimations are reported for an average individual aged 75 years old at time t = 0.

Italics indicate that p-values were no longer significant after correction for multiple testing with False Discovery Rate analyses.

Abbreviations: TC: Total cholesterol, LDL-C: Low-Density Lipoprotein cholesterol, HDL-C: High-Density Lipoprotein cholesterol; AIP: Atherogenic Index of Plasma.

**eFigure 1. Flowchart ^a^**

**^
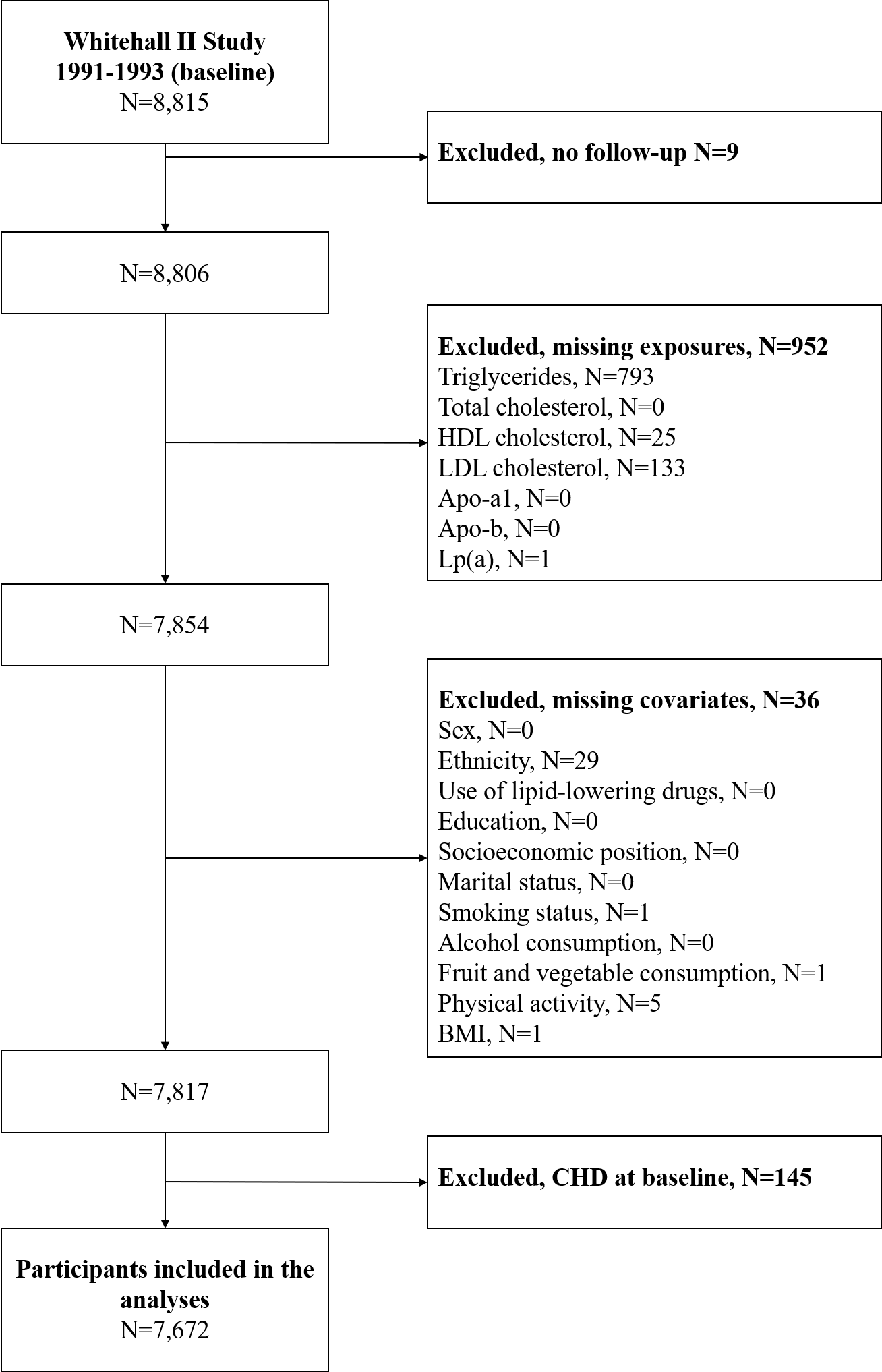
^**

^a^Abbreviations: ApoA1: Apolipoprotein A1; ApoB: Apolipoprotein B; BMI, Body Mass Index; CHD: Coronary Heart Disease; HDL: High-Density Lipoprotein cholesterol; LDL: Low-Density Lipoprotein cholesterol; Lp(a): Lipoprotein (a).

**eFigure 2. Trajectories of lipids over 28 years before dementia, mortality, and coronary heart disease women using a backward timescale and adjusting for menopausal status.^a^**
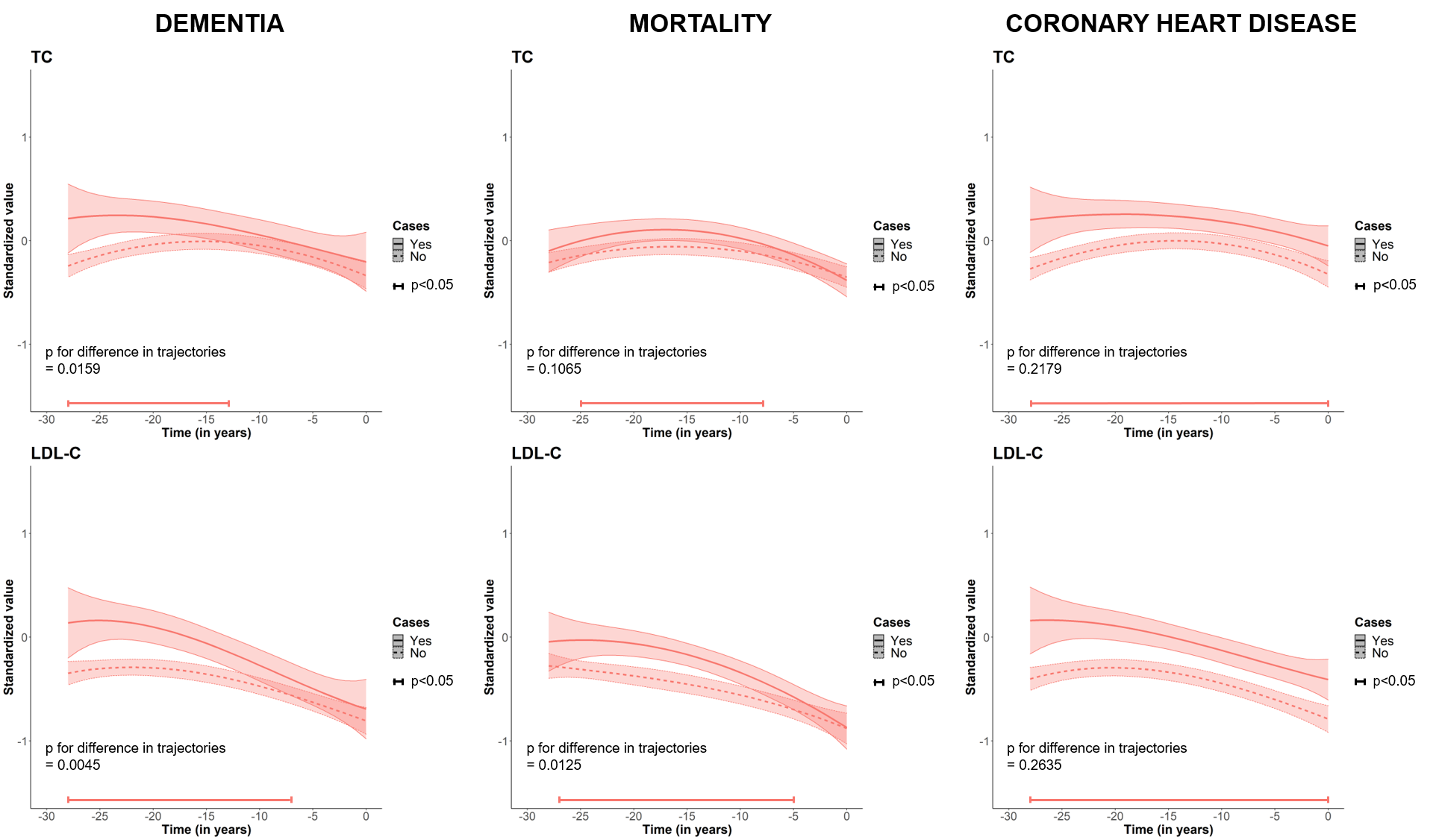


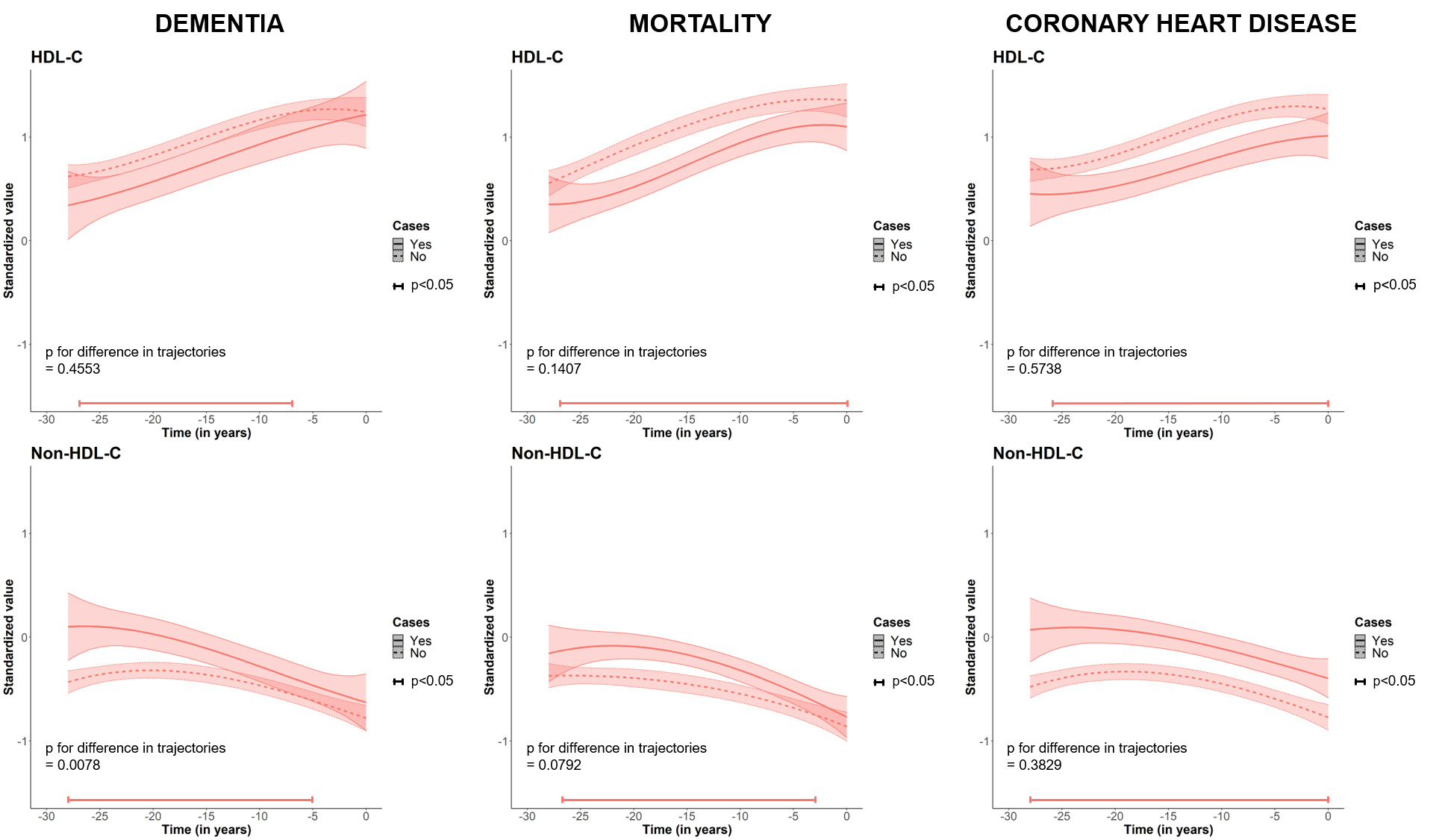

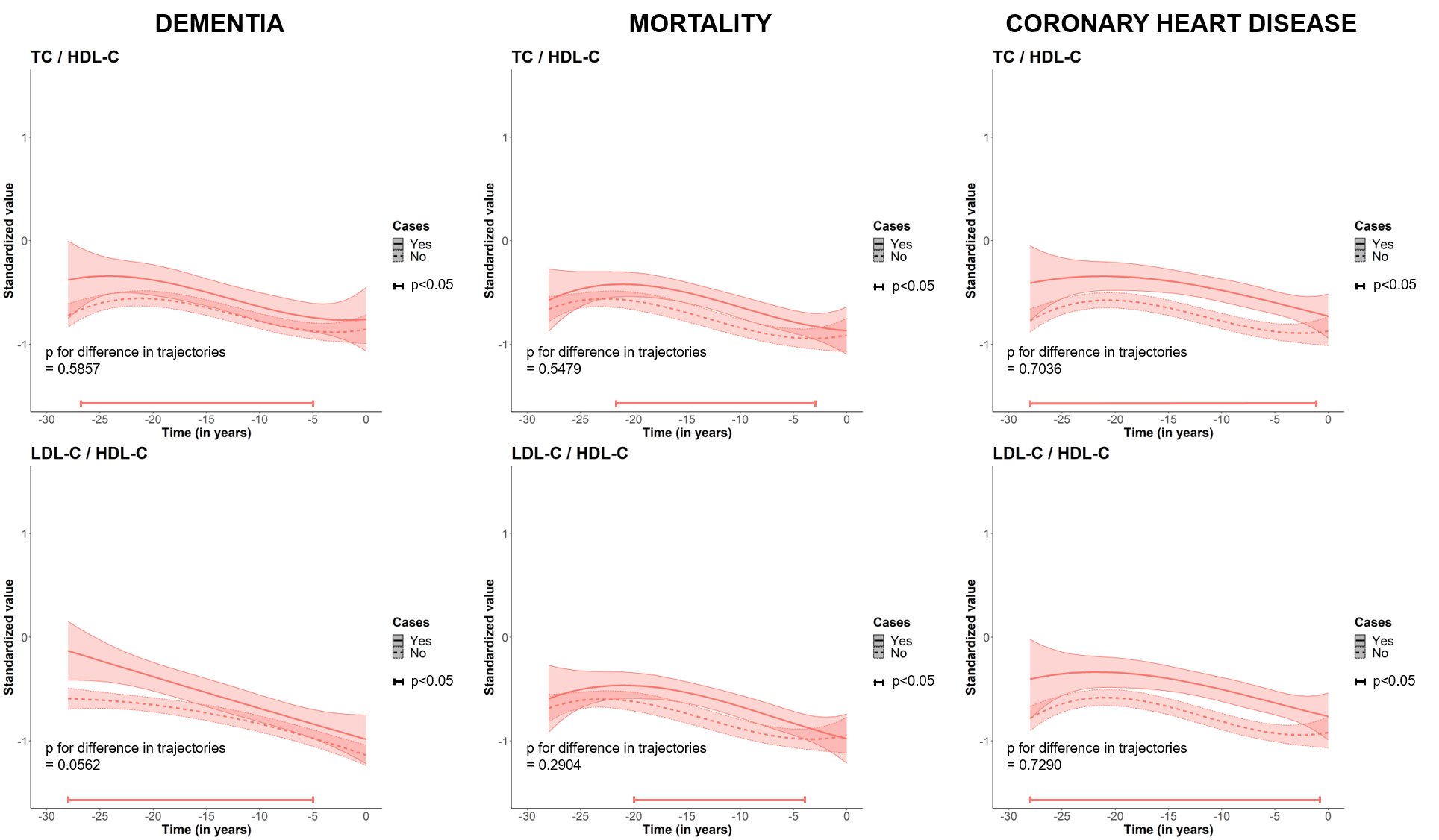

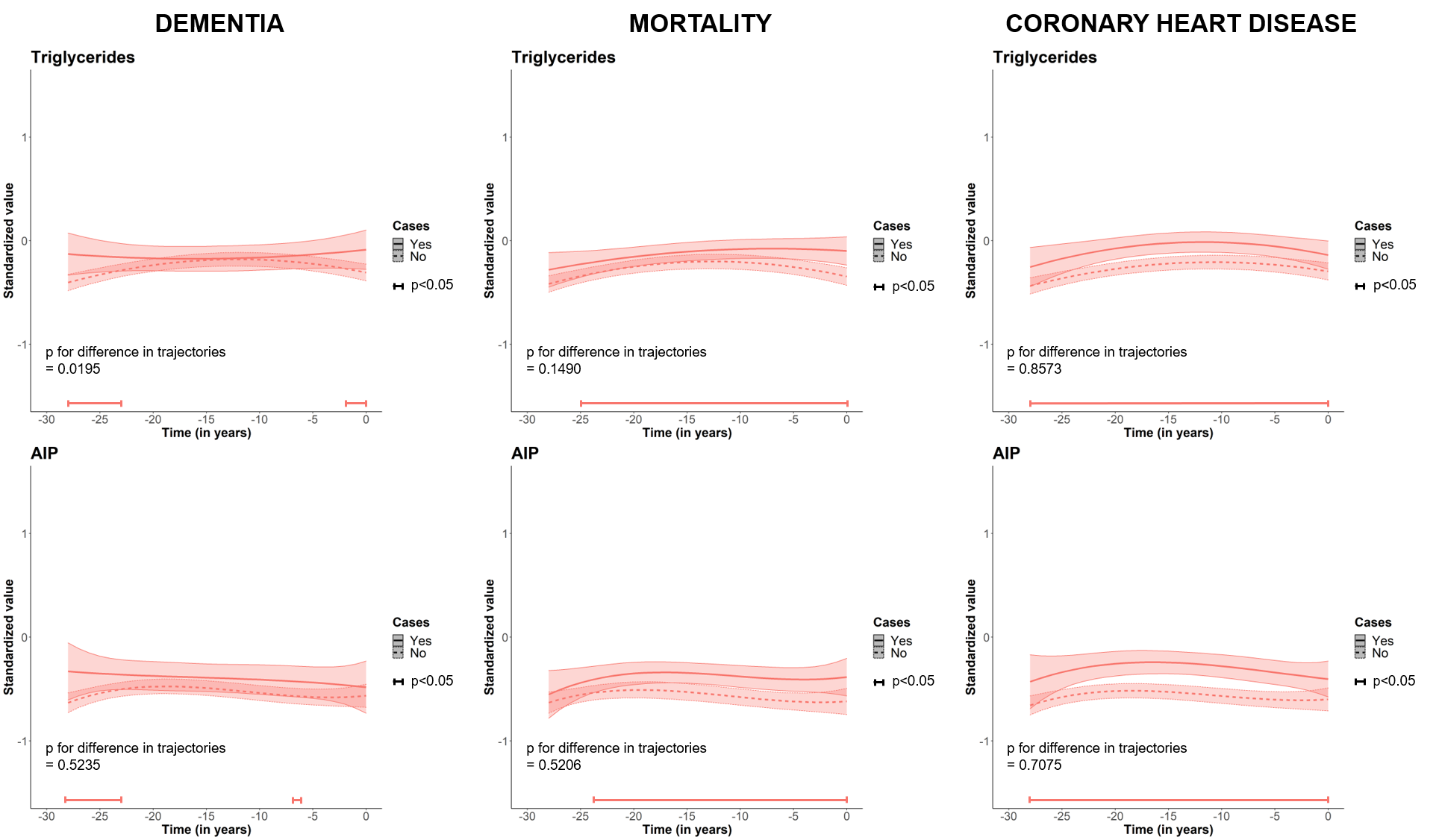


^a^Estimated marginal means based on linear mixed models with a backward scale of time (time before occurrence of the event, in years, with t = 0 defined as the time of the event or 31^st^ of March 2019, whichever came first). The analyses were adjusted for menopausal status (time-varying), use of lipids-lowering drugs (time-varying), ethnicity, marital status (time-varying), education, socioeconomic position (time-varying), health-related behaviours (smoking status, alcohol consumption, fruit and vegetable consumption, physical activity, and obesity, all time-varying), a binary indicator of data from 1991-1993 (pre-statin era), age at t = 0, and event status at t = 0. These models included an interaction term between time and event status at t=0 as well as between time and age at t = 0. Estimations are reported for an average individual aged 75 years old at time t = 0.

Abbreviations: TC: Total cholesterol, LDL-C: Low-Density Lipoprotein cholesterol, HDL-C: High-Density Lipoprotein cholesterol; AIP: Atherogenic Index of Plasma.

**STROBE Statement—Checklist of items that should be included in reports of *cohort studies***

|  | Item No | Recommendation | Page No |
| --- | --- | --- | --- |
| **Title and abstract** | 1 | (*a*) Indicate the study’s design with a commonly used term in the title or the abstract | 1, 2 |
|  |  | (*b*) Provide in the abstract an informative and balanced summary of what was done and what was found | 2 |
| Introduction | | | |
| Background/rationale | 2 | Explain the scientific background and rationale for the investigation being reported | 3 |
| Objectives | 3 | State specific objectives, including any prespecified hypotheses | 4 |
| Methods | | | |
| Study design | 4 | Present key elements of study design early in the paper | 4-5 |
| Setting | 5 | Describe the setting, locations, and relevant dates, including periods of recruitment, exposure, follow-up, and data collection | 4-5 |
| Participants | 6 | (*a*) Give the eligibility criteria, and the sources and methods of selection of participants. Describe methods of follow-up | 4-5 |
|  |  | (*b*) For matched studies, give matching criteria and number of exposed and unexposed |  |
| Variables | 7 | Clearly define all outcomes, exposures, predictors, potential confounders, and effect modifiers. Give diagnostic criteria, if applicable | 4-6 |
| Data sources/ measurement | 8* | For each variable of interest, give sources of data and details of methods of assessment (measurement). Describe comparability of assessment methods if there is more than one group | 4-6 |
| Bias | 9 | Describe any efforts to address potential sources of bias | 8 |
| Study size | 10 | Explain how the study size was arrived at |  |
| Quantitative variables | 11 | Explain how quantitative variables were handled in the analyses. If applicable, describe which groupings were chosen and why | 6-8 |
| Statistical methods | 12 | (*a*) Describe all statistical methods, including those used to control for confounding | 6-8 |
|  |  | (*b*) Describe any methods used to examine subgroups and interactions |  |
|  |  | (*c*) Explain how missing data were addressed |  |
|  |  | (*d*) If applicable, explain how loss to follow-up was addressed |  |
|  |  | (*e*) Describe any sensitivity analyses |  |
| Results | | |  |
| Participants | 13* | (a) Report numbers of individuals at each stage of study—eg numbers potentially eligible, examined for eligibility, confirmed eligible, included in the study, completing follow-up, and analysed | 9 |
|  |  | (b) Give reasons for non-participation at each stage |  |
|  |  | (c) Consider use of a flow diagram |  |
| Descriptive data | 14* | (a) Give characteristics of study participants (eg demographic, clinical, social) and information on exposures and potential confounders | 9 + Table 1, Table2, eTable 1, eTable 2, eTable 3, eTable 4 |
|  |  | (b) Indicate number of participants with missing data for each variable of interest |  |
|  |  | (c) Summarise follow-up time (eg, average and total amount) |  |
| Outcome data | 15* | Report numbers of outcome events or summary measures over time | 9-10 |

| Main results | 16 | (*a*) Give unadjusted estimates and, if applicable, confounder-adjusted estimates and their precision (eg, 95% confidence interval). Make clear which confounders were adjusted for and why they were included | 9-11 |
| --- | --- | --- | --- |
|  |  | (*b*) Report category boundaries when continuous variables were categorized |  |
|  |  | (*c*) If relevant, consider translating estimates of relative risk into absolute risk for a meaningful time period |  |
| Other analyses | 17 | Report other analyses done—eg analyses of subgroups and interactions, and sensitivity analyses | 11 |
| Discussion | | | |
| Key results | 18 | Summarise key results with reference to study objectives | 12 |
| Limitations | 19 | Discuss limitations of the study, taking into account sources of potential bias or imprecision. Discuss both direction and magnitude of any potential bias | 15 |
| Interpretation | 20 | Give a cautious overall interpretation of results considering objectives, limitations, multiplicity of analyses, results from similar studies, and other relevant evidence | 12, 15 |
| Generalisability | 21 | Discuss the generalisability (external validity) of the study results | 15 |
| Other information | | | |
| Funding | 22 | Give the source of funding and the role of the funders for the present study and, if applicable, for the original study on which the present article is based | 20 |
